# Synthesis, Characterization, and Biological Evaluation of New Derivatives Targeting MbtI as Antitubercular Agents

**DOI:** 10.3390/ph14020155

**Published:** 2021-02-13

**Authors:** Matteo Mori, Giovanni Stelitano, Laurent R. Chiarelli, Giulia Cazzaniga, Arianna Gelain, Daniela Barlocco, Elena Pini, Fiorella Meneghetti, Stefania Villa

**Affiliations:** 1 Department of Pharmaceutical Sciences, University of Milan, Via L. Mangiagalli 25, 20133 Milano, Italy; matteo.mori@unimi.it (M.M.); giulia.cazzaniga@unimi.it (G.C.); arianna.gelain@unimi.it (A.G.); daniela.barlocco@unimi.it (D.B.); elena.pini@unimi.it (E.P.); stefania.villa@unimi.it (S.V.); 2 Department of Biology and Biotechnology “Lazzaro Spallanzani”, University of Pavia, via A. Ferrata 9, 27100 Pavia, Italy; giovanni.stelitano01@universitadipavia.it (G.S.); laurent.chiarelli@unipv.it (L.R.C.)

**Keywords:** tuberculosis, mycobactins, furan, siderophores, drug design, bioisosterism, drug resistance

## Abstract

Tuberculosis (TB) causes millions of deaths every year, ranking as one of the most dangerous infectious diseases worldwide. Because several pathogenic strains of *Mycobacterium tuberculosis* (Mtb) have developed resistance against most of the established anti-TB drugs, new therapeutic options are urgently needed. An attractive target for the development of new antitubercular agents is the salicylate synthase MbtI, an essential enzyme for the mycobacterial siderophore biochemical machinery, absent in human cells. A set of analogues of **I** and **II**, two of the most potent MbtI inhibitors identified to date, was synthesized, characterized, and tested to elucidate the structural requirements for achieving an efficient MbtI inhibition and a potent antitubercular activity with this class of compounds. The structure-activity relationships (SAR) here discussed evidenced the importance of the furan as part of the pharmacophore and led to the preparation of six new compounds (**IV**–**IX**), which gave us the opportunity to examine a hitherto unexplored position of the phenyl ring. Among them emerged 5-(3-cyano-5-(trifluoromethyl)phenyl)furan-2-carboxylic acid (**IV**), endowed with comparable inhibitory properties to the previous leads, but a better antitubercular activity, which is a key issue in MbtI inhibitor research. Therefore, compound **IV** offers promising prospects for future studies on the development of novel agents against mycobacterial infections.

## 1. Introduction

Tuberculosis (TB), the infectious disease caused by *Mycobacterium tuberculosis* (Mtb), represents a global emergency requiring new therapeutic options, mainly because of the rapid spread of drug-resistant strains, which are causing an alarming rise in clinical cases.

According to the 2020 World Health Organization (WHO) report [1], TB was responsible for around 1.4 million deaths and over 10 million new infections worldwide in 2019; these numbers are expected to rise significantly in 2020 as a consequence of the coronavirus disease 2019 (COVID-19) pandemic. Additionally, it is estimated that Mtb exists in its latent form in approximately one-quarter of the global population [1].

Although the investigation of new pharmaceutical forms for the delivery of current antitubercular drugs may contribute to enhance patient compliance and limit the spread of the disease [2,3], the development of new therapeutic options represents an even more pressing need. While drug-susceptible TB can be cured within 6–8 months with the current standard treatment regimen [4], multi- and extensively drug-resistant (MDR/XDR) infections are treated for at least 20 months with poor outcomes [5], posing a serious threat to human health. The continuous genetic adaptation and rapid propagation of drug-resistant pathogens have led to an expected drop in the therapeutic efficacy of the current anti-TB drugs, forcing the scientists to face new challenges in the discovery of novel molecular entities to address this issue. Hence, the development of innovative compounds targeting both replicating and dormant Mtb bacilli is critical for the design of more effective and shorter therapies.

To address the urgent need of selective antitubercular drugs with novel mechanisms of action, new drug targets have been recently explored and validated [6,7,8]. Among them, the mycobactin biosynthetic pathway, which leads to the synthesis of siderophores capable of sequestering host iron, has been identified as a source of promising candidates [9,10]. Indeed, the siderophore biochemical machinery is significantly upregulated under iron-deficient conditions, common in infected macrophages, constituting one of the major pathogenic determinants of TB. Moreover, it is absent in humans, thus minimizing the risk of off-target effects.

**Figure 1:**
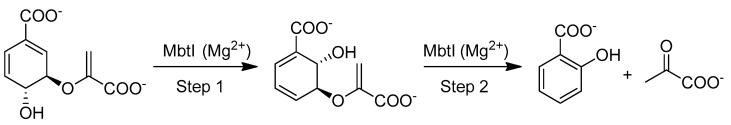
Reactions catalyzed by MbtI.

Among the four enzymes involved in mycobactin biosynthesis and currently under investigation as potential drug targets (i.e., MbtI, MbtA, MbtM, and PPTase), we focused our attention on the Mg^2+^-dependent bifunctional salicylate synthase MbtI, which catalyzes the first two steps in the production of all mycobacterial siderophores. This enzyme belongs to the family of the chorismate-utilizing enzymes (CUEs) [11] and it catalyzes the reactions shown in Figure 1.

**Figure 2:**
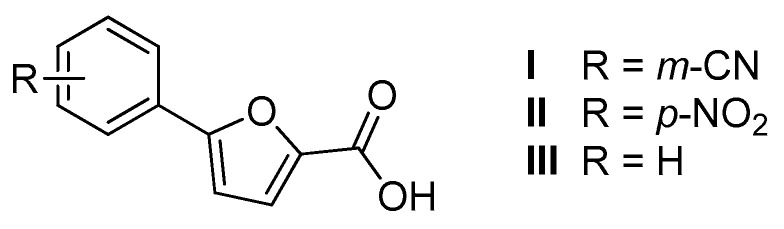
Chemical structure of the lead compounds **I**, **II**, and **III**.

In this context, we developed in recent years a series of furan-based carboxylic acids as MbtI inhibitors [12,13,14,15]. Among this class of compounds, **I** and **II** (Figure 2) emerged as the best leads, exhibiting a strong MbtI inhibition, conceivably related to their antitubercular activity, and a negligible cytotoxicity towards eukaryotic cells. When analyzing the structure-activity relationships (SAR) of these compounds, we observed that the activity of the substances was closely related to the presence of an electron withdrawing moiety in a suitable position of the phenyl ring. The removal of the substituent from the phenyl of our furan-based leads (**III**, Figure 2) resulted in a complete loss of activity [14].

Encouraged by these studies, and with the aim of enriching our arsenal of MbtI inhibitors with compounds exhibiting enhanced antitubercular activities, we enlarged our set of derivatives to include compounds bearing different heterocyclic scaffolds.

**Figure 3:**
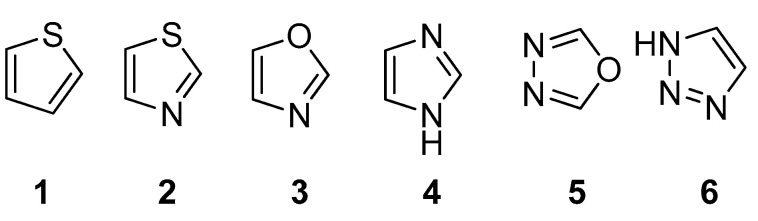
Chemical structure of the heterocyclic cores tested in this study: **1** (thiophene), **2** (thiazole), **3** (oxazole), **4** (imidazole), **5** (1,3,4-oxadiazole), **6** (1,2,3-triazole).

In some literature cases, the furan core was successfully replaced by other heterocycles to improve the cellular activity; indeed, according to Hinsberg’s “ring equivalence” theory, the concept of isosterism and bioisosterism can be extended to heterocycles [16]. Here, we investigated whether the furan moiety could be successfully replaced by any of the heterocycles shown in Figure 3, also considering that extensive research efforts have been devoted to the exploration of heterocyclic compounds as antimycobacterial agents [17].

We considered the introduction of a thiophene (**1**), because, in several literature examples, the use of this ring has resulted in an improvement of the antimycobacterial activity [18,19,20]. The furan was then substituted by a thiazole (**2**); aside from being the most common heterocycle in drug design [21], this ring is part of the chemical structure of many compounds endowed with antitubercular activity [22]. To expand our investigations, we synthesized two derivatives bearing an oxazole (**3**), where the sulfur atom of the thiazole ring is substituted by an oxygen, a sulfur isostere [23]. The imidazole was then selected as an attractive isostere of thiazole and oxazole; notably, nitroimidazopyran PA-824 [24] has recently moved to advanced-stage clinical trials, inspiring the development of anti-TB agents featuring this moiety [25]. Finally, we explored the 1,3,4-oxadiazole (**5**), as it was reported to interact with some of the newest anti-TB targets [26], and the 1,2,3-triazole (**6**), whose importance is demonstrated by the antitubercular agent I-A09, which is under preclinical trials [27].

In the first part of this work, we designed, synthesized, and evaluated the biological activity of novel heterocyclic compounds belonging to two homologous series (Table 1), bearing the *m*-CN (series A, compounds **1a**–**6a**) and *p*-NO_2_ (series B, **1b**–**6b**) substituent, respectively.

The modest biological activity of the new derivatives prompted us to reconsider the furan as the best heterocyclic core, suggesting the critical nature of an appropriately substituted furan moiety to maintain a significant enzymatic inhibition and to achieve antitubercular activity.

**Figure 4:**
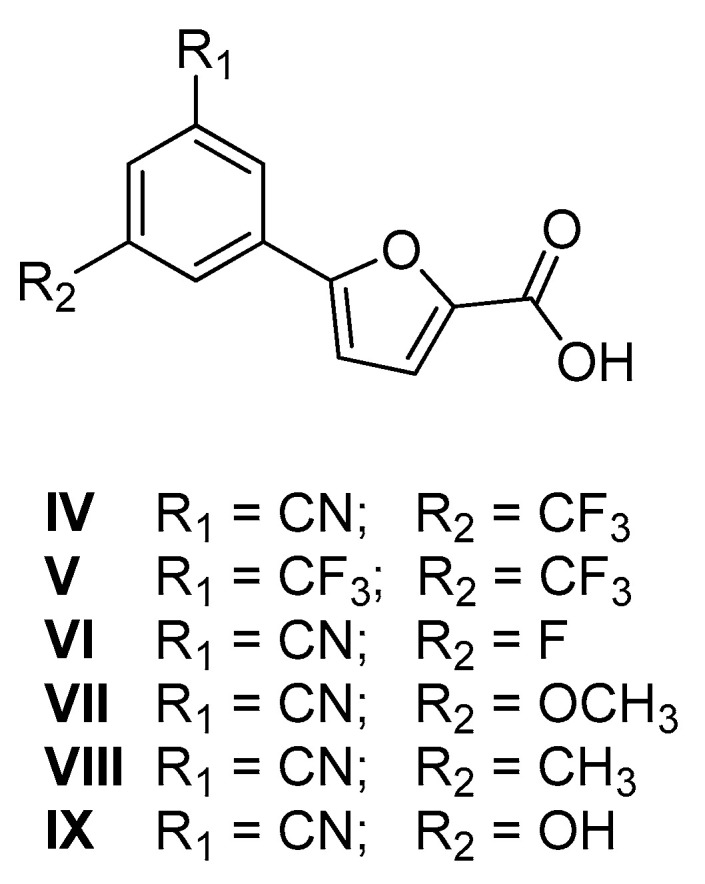
Chemical structure of compounds **IV**–**IX**.

On this basis, and considering our previous results on disubstituted derivatives, we decided to design and synthesize six new furan-based analogues (**IV**–**IX**, Figure 4). In particular, **IV** was investigated as the isomer of 5-(2-cyano-4-(trifluoromethyl)phenyl)furan-2-carboxylic acid, which exhibited an interesting inhibitory effect (IC_50_ of about 18 µM) [13]. The new analogue **IV** bears the CN and CF_3_ moieties in the relative *meta* positions to avoid the steric interactions between the two adjacent groups, which had proven to be detrimental for the activity [13]. Compound **V** was synthesized to evaluate the role of the 3-CN moiety, as our past works had shown that this group was superior to other substituents in terms of enzymatic activity [12,13].

Finally, compounds **VI**–**IX** were prepared to examine the influence of the substituent at position 5 of the phenyl ring on the biological activity of this class of compounds.

This strategy gave us the opportunity to examine a hitherto unexplored position of the phenyl ring (**IV**–**IX**), leading to the discovery of novel MbtI inhibitors endowed with antimycobacterial activity.

## 2. Results

### 2.1. Chemistry

**Scheme 1:**
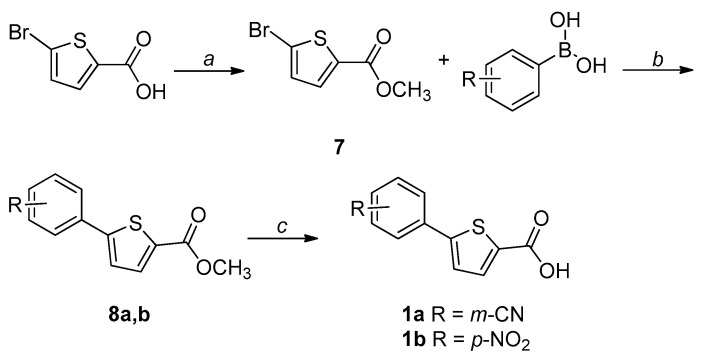
Synthetic procedure for the preparation of **1a,b**. Reagents and conditions: (**a**) MeOH, conc. H_2_SO_4_, reflux, overnight; (**b**) Pd(PPh_3_)_2_Cl_2_, 2 M Na_2_CO_3_, dry 1,4-dioxane, 90 °C, overnight, N_2_ atm; (**c**) *1.* LiOH, THF-H_2_O 2:1, r.t., 2 h for **1a**; 1 M NaOH, EtOH-THF 1:1, reflux, 5 h for **1b**; *2.* 1 M HCl, 0 °C.

**Scheme 2:**
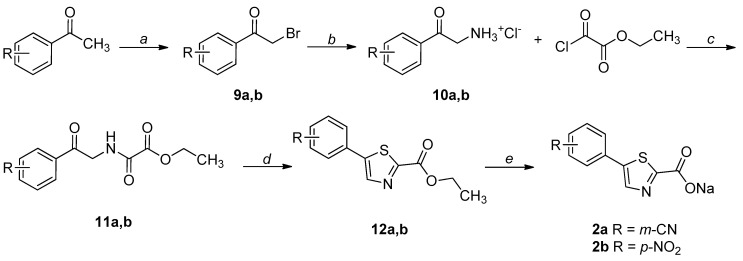
Synthetic procedure for the preparation of **2a,b**. Reagents and conditions: (**a**) NBS, *p*-TsOH, DCM, overnight, r.t, N_2_ atm.; (**b**) *1*. hexamine, DCM, 8 h, r.t.; *2*. conc. HCl, EtOH, overnight, r.t.; (**c**) TEA, EtOAc, 3 h, reflux; (**d**) Lawesson’s reagent, 1,4-dioxane, 2 h, reflux; (**e**) NaOH, THF-H_2_O 1:1, 1.5 h, r.t.

**Scheme 3:**
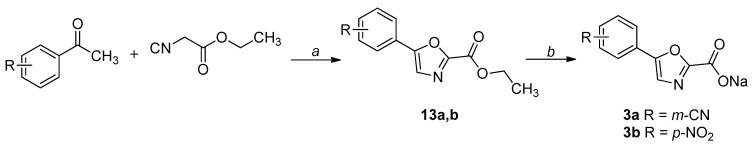
Synthetic procedure for the preparation of **3a,b**. Reagents and conditions: (**a**) I_2_, DMSO, 3 h, 130 °C; (**b**) NaOH, THF-H_2_O 1:1, 1.5 h, r.t.

**Scheme 4:**
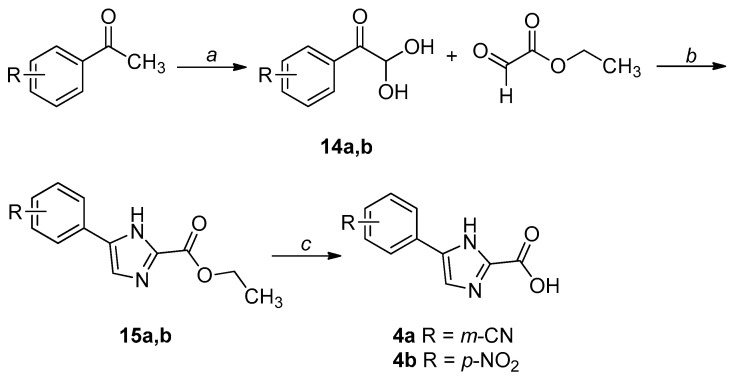
Synthetic procedure for the preparation of **4a,b**. Reagents and conditions: (**a**) SeO_2_, 1,4-dioxane/H_2_O, reflux, 7 h, N_2_ atm; (**b**) NH_4_OAc, CH_3_CN, H_2_O, r.t., 2 h; (**c**) *1.* LiOH, THF-H_2_O 2:1, r.t., overnight for **4a**; NaOH, THF-H_2_O 1:1, reflux, 6 h for **4b**; *2.* 3 M HCl, 0 °C.

**Scheme 5:**
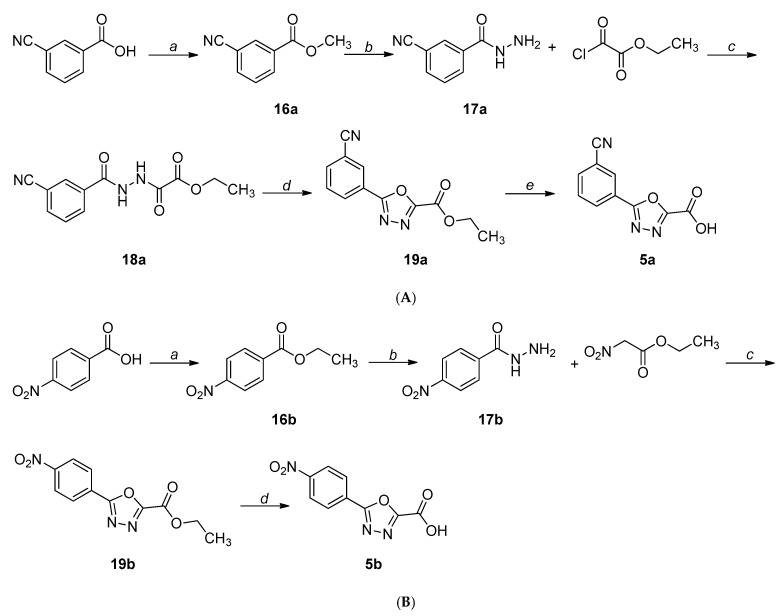
(**A**) Synthetic procedure for the preparation of **5a**. Reagents and conditions: (**a**) dry MeOH, conc. H_2_SO_4_, reflux, 3 h, N_2_ atm; (**b**) NH_2_NH_2_∙H_2_O, MeOH, r.t., overnight; (**c**) TEA, DCM, r.t., 2 h; (**d**) TEA, DCM, TsCl, r.t., 2 h; (**e**) *1.* NaOH, THF-H_2_O 1:1, r.t., 1 h; *2.* Amberlite IR120, 0 °C. (**B**) Synthetic procedure for the preparation of **5b**. Reagents and conditions: (**a**) EtOH, conc. H_2_SO_4_, reflux, overnight; (**b**) NH_2_NH_2_∙H_2_O_,_ EtOH, reflux, overnight; (**c**) 86% PPA, 120-130 °C, 1.5 h; (**d**) *1.* LiOH, THF-H_2_O 1:1, r.t., 1 h; *2.* Amberlite IR120, 0 °C.

**Scheme 6:**
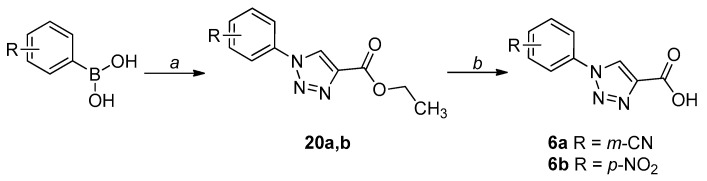
Synthetic procedure for the preparation of **6a,b**. Reagents and conditions: (**a**) *1*. NaN_3_, Cu(OAc)_2_, MeOH, 55 °C, 1.5-4 h, N_2_ atm; *2.* ethyl propiolate, (+)-sodium l-ascorbate, r.t., overnight-24 h; (**b**) *1*. LiOH, THF-H_2_O 2:1, r.t., 1 h for **6a**; NaOH, THF-H_2_O 1:1, reflux, 5 h for **6b**; *2.* 3 M HCl, 0 °C for **6a**; 1 M HCl, 0 °C for **6b**.

The synthetic procedures adopted for the preparation of the compounds are heterogeneous and reflect the diverse approaches needed for the obtainment of the various heterocyclic derivatives. Where possible, the synthetic strategies were designed and optimized to afford the desired compounds, starting from the same commercially available reagents. All the compounds were characterized by means of mono- and bi-dimensional NMR techniques, FT-IR, ESI-MS, and elemental analysis. The procedures for the synthesis of series A and B (compounds **1a,b**–**6a,b**) are depicted in Scheme 1, Scheme 2, Scheme 3, Scheme 4, Scheme 5 and Scheme 6; all details regarding procedures and analytical data are reported in the Supplementary Materials.

The key intermediate (**7**) for the synthesis of **1a**,**b** was obtained through a Fischer–Speier esterification of the commercially available 5-bromo-2-thiophenecarboxylic acid. Then, a palladium-catalyzed Suzuki-Miyaura coupling led to **8a,b**, which were hydrolyzed to the corresponding acids (**1a,b**) under basic conditions (Scheme 1).

The synthesis of the thiazole-based derivatives (**2a,b**) started from the bromination of the appropriate acetophenone, leading to **9a,b**. The hydrochloride salts of the corresponding amines (**10a,b**), obtained through the Delépine reaction, were *N*-acylated with ethyl chlorooxoacetate to afford the corresponding amides (**11a,b**). The formation of the thiazole ring was performed using the Lawesson’s reagent, leading to the esters **12a,b**, which were hydrolyzed under basic conditions and isolated as sodium salts (Scheme 2) [28].

Oxazole-based derivatives were obtained through an iodine-promoted formal [3+2] cycloaddition of methyl ketones to α-methylenyl isocyanides: in particular, ethyl isocyanoacetate was reacted with the suitable acetophenone to afford 2,5-disubstituted oxazole esters (**13a,b**). The intermediates were then hydrolyzed under basic conditions and isolated as sodium salts (Scheme 3) [29].

For the synthesis of **4a,b**, the suitably substituted geminal diols **14a,b** were obtained from the commercially available acetophenone in the presence of selenium dioxide. Then, these intermediates were reacted with ethyl 2-oxoacetate and ammonium acetate, affording the imidazole esters **15a,b**, which were finally hydrolyzed to the corresponding acids (**4a,b**) under basic conditions (Scheme 4) [30].

For the synthesis of the *m*-CN-substituted 1,3,4-oxadiazole derivative (**5a**), the commercially available 3-cyanobenzoic acid was converted to the corresponding methyl ester (**16a**) and reacted with hydrazine hydrate to afford the hydrazide **17a**. This intermediate was acylated with ethyl-chlorooxoacetate to **18a**, which was cyclized to **19a** using *p*-toluensulfonyl chloride in the presence of triethylamine. The oxadiazole ester was then hydrolyzed under basic conditions to give **5a** (Scheme 5A) [31].

Concerning the *p*-NO_2_-substituted 1,3,4-oxadiazole analogue (**5b**), the hydrazide **17b**, obtained as described above, was reacted with ethyl 2-nitroacetate in polyphosphoric acid to afford the ester **19b**, which was hydrolyzed to the corresponding acid (**5b**) under basic conditions (Scheme 5B) [32].

The 1,4-substituted triazole esters **20a,b** were obtained through a one-pot Huisgen cycloaddition, starting from the appropriate phenylboronic acid: the synthesis of the azide was followed by the addition of ethyl propiolate, leading to the desired intermediates. The final acids (**6a,b**) were obtained through a base-catalyzed hydrolysis of the ester function (Scheme 6).

The new furan derivatives **IV**–**IX** were synthesized according to previously published procedures [13]. **V** was obtained by the same approach adopted for **1a**,**b**, employing (3,5-bis(trifluoromethyl)phenyl)boronic acid and methyl 5-bromofuran-2-carboxylate in a traditional Suzuki–Miyaura reaction, followed by a hydrolysis of the ester function [13]. **IV** was obtained by reacting 3-bromo-5-(trifluoromethyl)benzonitrile with (5-(methoxycarbonyl)furan-2-yl)boronic acid in a microwave-assisted Suzuki-Miyaura coupling, followed by a base-catalyzed hydrolysis of the ester function [13]. Similarly, 3-bromo-5-fluorobenzonitrile, 3-bromo-5-methoxybenzonitrile, 3-bromo-5-methylbenzonitrile, and 3-bromo-5-hydroxybenzonitrile were used as starting compounds for **VI**, **VII**, **VIII**, and **IX**, respectively.

### 2.2. Biological Studies

To pursue our aim of investigating the role of the heterocyclic core in MbtI inhibition, we decided to compare two sets of data, derived from our previous leads: **I**, characterized by the presence of the *m*-CN group (series A), and **II**, bearing the less druggable *p*-NO_2_ group, but capable of potently inhibiting MbtI (series B) [12]. Therefore, keeping the cyano and the nitro group in their original positions, we explored the effects of the variation of the five-membered ring on the activity against the enzyme. The results of the in vitro assays on compounds **1a,b–6a,b**, calculated as previously reported [12], are listed in Table 1.

As for the thiophene analogues, the biological tests showed that **1a** and **1b** are approximately equipotent, with 23% residual enzymatic activity at 100 µM (%RA). The corresponding thiazole derivatives **2a** and **2b** are weaker inhibitors, especially in the presence of the *p*-NO_2_ substitution. In some cases, thiazoles have been identified as pan-assay interference compounds (PAINS) [33]. To exclude this possibility, we tested **2a** and **2b**, along with all the other compounds published herein, against the PAINS filters of four online-based services, namely FAF-Drugs4 [34], SmartsFilter (https://chiltepin.health.unm.edu/tomcat/biocomp/smartsfilter accessed on 12 January 2021), SwissADME [35], and Zinc Patterns (http://zinc15.docking.org/patterns/home/ accessed on 12 January 2021). Notably, none of the molecules were identified as potential PAINS. As for the oxazole derivatives, **3a** showed a negligible activity, while **3b** evidenced a modest activity. Although we had envisioned that the structural features of the imidazole ring could be beneficial to form interactions within the MbtI active site, derivatives **4a** and **4b** displayed only a weak activity. Finally, the replacement of the furan with the oxadiazole and triazole cores in **5a**–**b** and **6a**–**b**, respectively, afforded compounds devoid of any significant effect against MbtI.

The minimal inhibitory concentration (MIC^99^) of the derivatives exhibiting an IC_50_ lower than 30 μM was determined against the nonpathogenic *M. bovis* BCG, in iron-limiting conditions (chelated Sauton’s medium), using the resazurin reduction assay method (REMA). All of them displayed MIC^99^ values greater than 250 μM, which did not represent a significant improvement with respect to the previous leads.

In light of these findings, and considering previous SAR data, we designed the new derivatives **IV**–**IX**. The furan ring was chosen as the central core of these compounds, because it proved to be the best option and an important portion of our pharmacophore model. This additional investigation was undertaken to explore a new position of the phenyl ring, with the final goal of identifying the structural requirements needed to improve the antitubercular potency of these compounds.

The disubstituted derivatives **IV**–**IX** were tested for their effects against the recombinant MbtI, prepared, and assayed as previously reported [12]; their in vitro activities are shown in Table 2.

Compound **IV** was designed with the cyano group in the *meta* position because our past works had shown that this group was superior to other options in terms of enzymatic activity; moreover, it features a trifluoromethyl moiety in 5, which allowed us to explore a hitherto unconsidered substitution pattern on the phenyl ring. **IV** was found to be a potent MbtI inhibitor (≈ 1% RA at 100 μM), with an IC_50_ of ≈ 15 μM. Compound **V**, carrying two trifluoromethyl moieties, was also effective against MbtI, though displaying a slightly higher IC_50_ with respect to **IV** (≈ 19 μM vs. 15 µM). Then, we tested compounds **VI**–**IX**, maintaining the original cyano group in 3 and featuring different moieties in 5. Firstly, we assayed the fluorine-substituted compound **VI**, which showed an IC_50_ value similar to that of **V** (IC_50_ ≈17 μM vs. 15 µM). Compound **VII**, bearing a methoxy moiety in 5, displayed a comparable IC_50_ with respect to **IV**. Derivatives **VIII** and **IX**, carrying the CH_3_ and the OH groups respectively, showed weaker inhibitory properties compared to compound **IV** (IC_50_ ≈ 29 μM and 33 µM) (see Table 2).

The four candidates exhibiting promising inhibitory properties (IC_50_ < 30 μM) were tested for their antimycobacterial activities against the nonpathogenic *M. bovis* BCG, in iron-limiting conditions (chelated Sauton’s medium), using the REMA method. In this assay, compound **IV** showed the best antimycobacterial activity, with a MIC^99^ value of 125 µM.

Due to its better bactericidal activity, **IV** emerged as the best inhibitor out of this furan series: its halved MIC^99^ compared to **I** (125 µM vs. 250 μM) highlighted the better drugability of this compound with respect to our previous candidates.

**Figure 5:**
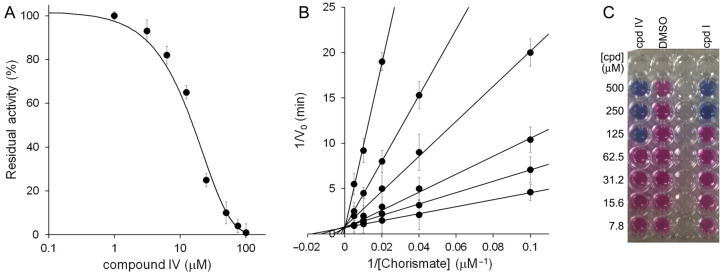
Biological characterization of **IV**. (**A**) IC_50_ determination of **IV** against MbtI activity. (**B**) Global reciprocal plot of data from MbtI steady-state kinetics analysis towards chorismic acid, in the presence of different concentrations of **IV** (50, 20, 10, 5, 1, and 0 μM). (**C**) MIC^99^ determination of **IV** against *M. bovis* BCG growth.

Therefore, we submitted compound **IV** to a kinetic analysis, which demonstrated the competitive nature of its inhibition against MbtI, with a K_i_ value of 9.2 ± 0.7 μM (Figure 5).

## 3. Discussion

In this work, we first applied a bioisosteric replacement strategy introducing in our leads **I** and **II** structural modifications in the five-membered core to alter the compound’s electronic distribution and lipophilicity, with the aim of improving the target engagement and the antimycobacterial activity.

Contrary to traditional bioisosteric principles, the biological profiles of the thiophene analogues were not comparable to those of the furan derivatives. Conversely, their inhibitory effect followed the general trend exhibited by the thiazole, oxazole, and imidazole derivatives, suggesting that other factors prevail over the bioisosterism of the two nuclei. Interestingly, a significant decline in the activity was observed in the oxadiazole- and triazole-based compounds. Regarding the substitution of the phenyl ring, the presence of the *m*-cyano or *p*-nitro groups did not seem to impact significantly on the variations of the activity.

In previous work, we reported the cocrystal structure of MbtI in complex with **I** and described the key interactions of the compound within the active site of the enzyme. Briefly, **I** forms H-bonds through its carboxylic group with Tyr385, Arg405, and an ordered water molecule; the oxygen of the furan interacts with Arg405, while the phenyl ring forms a cation-π interaction with Lys438 and a Van der Waals contact with Thr361. Finally, the cyano group interacts with Lys205, a key amino acid involved in the first step of the catalytic reaction. While the diminished activity of the triazole ring may be justified by the absence of a heteroatom capable of accepting a H bond from Arg405, the formulation of a hypothesis to explain the superiority of the furan with respect to the remaining cores is more arduous. A computational analysis of the binding modes of the tested compounds did not reveal significant disparities with respect to that of the lead molecule (unpublished data), suggesting that other influencing elements must be involved. Similarly, an in-silico comparison of the physicochemical characteristics of the compounds did not lead to meaningful results. Despite the different heterocyclic nuclei impart modifications to the overall properties of the molecules, a correspondence between the alteration of a parameter and the biological activity could not be unequivocally established. Therefore, it is reasonable to assume that the superior activity of the furan derivatives cannot be merely linked to the variation of one single parameter, but rather it is the result of a much more complex intertwinement of unrelated minimal modifications. The inherent multifactorial nature of these processes makes it hard, and potentially misleading, to seek a simplistic, univocal interpretation of these results. Hence, it is our opinion that the biological activity, empirically detected with our assays, is the only meaningful parameter that should be considered while determining the best heterocyclic core for this class of compounds.

In addition, when working with mycobacterial enzymes, in vitro activity may not necessarily correlate with the efficacy against the mycobacteria; therefore, compounds showing a weaker inhibitory effect against the purified target could display a better activity against bacterial growth, for instance due to an improved membrane permeability. On these bases, the MIC of the most potent molecules (IC_50_ < 30 μM) was determined against *M. bovis* BCG. Although, in some literature cases, the furan core was successfully replaced by other heterocycles to improve the cellular activity [36], this was not our case. None of the compounds belonging to series A and B exhibited improved antitubercular action, all of them having MIC^99^ values greater than 250 μM.

Overall, these biological results confirmed the furan as the best heterocyclic moiety among the options explored in this study and prompted us to reconsider this ring as the best scaffold to gain MbtI inhibition and antimycobacterial activity. In this regard, new modifications to the phenyl ring were attempted to improve the biological profile of our compounds. In our previous work, we discovered that the *m*-CN substitution offered the possibility to achieve better results in terms of enzyme inhibition compared to the other groups [12]. Moreover, in the context of a previously published disubstituted series, we took into account 5-(2-cyano-4-(trifluoromethyl)phenyl)furan-2-carboxylic acid, which proved to possess an interesting inhibitory effect (IC_50_ of about 18 µM). Therefore, we decided to explore the activity of its isomer 5-(3-cyano-5-(trifluoromethyl)phenyl)furan-2-carboxylic acid (**IV**, Figure 4), bearing the cyano group in the preferred position 3. Additionally, the relocation of the trifluoromethyl moiety to position 5 to avoid the steric interactions between adjacent groups allowed us to examine a hitherto unexplored substitution site on the phenyl ring. The new isomer revealed an interesting IC_50_ value of about 15 µM. Following the same strategy, we synthesized compound **V**, an isomer of 5-(2,4-bis(trifluoromethyl)phenyl)furan-2-carboxylic acid (IC_50_ of about 13 µM) [13]. The new analogue exhibited slightly lower inhibitory properties (IC_50_ ≈ 19 µM) with respect to the parent compound, while maintaining a promising activity. Subsequently, to further explore the SAR of the 5-substituent, we synthesized and tested compounds **VI**–**IX**, bearing the CN group in 3.

The presence of different substituents at position 5 of compounds **IV**–**IX** did not seem to significantly affect their inhibitory activity against MbtI, with IC_50_ ranging from 15 µM to 33 µM. By contrast, the 5-CF_3_ group of **IV** was able to significantly ameliorate its antimycobacterial properties with respect to the lead **I**. Indeed, **IV** displayed a MIC^99^ value of 125 µM, far better than that of **I** (250 µM).

Further biological studies demonstrated that **IV** was a competitive inhibitor of MbtI, exhibiting a K*i* of about 9 µM, roughly comparable to that of **I**.

Overall, these SAR observations demonstrated the essentiality of the furan core and the advantages of a 3,5-disubstitution of the phenyl ring to achieve a potent in vitro activity against MbtI and a significant antimycobacterial effect.

The improvement of the MIC of new compounds is a common goal in antitubercular drug discovery, as reported in a very recent review, which also supported the importance of the mycobactin biosynthetic pathway for the development of anti-TB agents [37]. The increased antitubercular activity of **IV** with respect to our previous leads opens new avenues for structural modifications towards improved candidates.

## 4. Material and Methods

### 4.1. Chemistry

All starting materials, chemicals, and solvents were purchased from commercial suppliers (Sigma-Aldrich, St. Louis, MI, USA; FluoroChem, Hadfield, UK; Carlo Erba, Cornaredo, Italy) and used as received. Anhydrous solvents were utilized without further drying. Aluminum-backed Silica Gel 60 plates (0.2 mm; Merck, Darmstadt, Germany) were used for analytical thin-layer chromatography (TLC), to follow the course of the reactions. Microwave-assisted reactions were carried out with a Biotage^®^ Initiator Classic (Biotage, Uppsala, Sweden). Silica gel 60 (40–63 μm; Merck) was used for the purification of intermediates and final compounds, through flash column chromatography. Melting points were determined in open capillary tubes with a Stuart SMP30 Melting Point Apparatus (Cole-Parmer Stuart, Stone, UK). All tested compounds were characterized by means of mono- and bi-dimensional NMR techniques, FT-IR, and ESI-MS. ^1^H and ^13^C NMR spectra were acquired at ambient temperature with a Varian Oxford 300 MHz instrument (Varian, Palo Alto, CA, USA) or a Bruker Avance 300 MHz instrument (Bruker, Billerica, MA, USA), operating at 300 MHz for ^1^H and 75 MHz for ^13^C. Chemical shifts are expressed in ppm (δ), while *J*-couplings are given in Hertz. The full decoupling mode was employed for ^13^C spectra when the relaxation times of the carbons did not allow for a sufficient resolution using the APT sequence. The 2D-NOESY sequence was employed to unambiguously assign the hydrogen signals, when appropriate. HMBC and HSQC analyses were performed to aid the assignment of ^13^C NMR signals, when necessary. ATR-FT-IR spectra were acquired with a Perkin Elmer Spectrum One FT-IR (Perkin Elmer, Waltham, MA, USA), equipped with a Perkin Elmer Universal ATR sampling accessory consisting of a diamond crystal. Analyses were performed in a spectral region between 4000 and 650 cm^−1^ and analyzed by transmittance technique with 28 scansions and 4 cm^−1^ resolution. MS analyses were carried out with a Thermo Fisher (Waltham, MA, USA) LCQ Fleet system, equipped with an ESI electrospray ionization source and an Ion Trap mass analyzer; ionization: ESI positive or ESI negative; capillary temperature: 250 °C; source voltage: 5.50 kV; source current: 4.00 μA; multipole 1 and 2 offset: −5.50 V and −7.50 V, respectively; intermultipole lens voltage: −16.00 V; trap DC offset voltage: −10.00 V. The purity of the tested compounds was assessed by means of elemental analysis using a EuroVector EA 3000 CHNS-O analyzer (EuroVector, Pavia, Italy). All experimental values are within ± 0.40% of the theoretical predictions, indicating a ≥ 95% purity.

All synthetic procedures are reported in the Supplementary Materials (SM).

***5-(3-Cyanophenyl)thiophene-2-carboxylic acid (1a).*** The compound was synthesized by a specific procedure, reported in SM. Aspect: white solid. Mp: 207 °C. TLC (DCM-MeOH 9:1): R_f_ = 0.20. The following analytical data are referred to the sodium salt. ^1^H NMR (300 MHz, DMSO-*d_6_*) δ 8.09 (t, *J* = 1.7 Hz, 1H, H_6_), 7.91 (ddd, *J* = 7.9, 2.0, 1.2 Hz, 1H, H_8_), 7.70 (dt, *J* = 7.7, 1.4 Hz, 1H, H_10_), 7.57 (t, *J* = 7.8 Hz, 1H, H_9_), 7.48 (d, *J* = 3.7 Hz, 1H, H_3_), 7.21 (d, *J* = 3.7 Hz, 1H, H_4_) ppm. ^13^C NMR (75 MHz, DMSO-*d_6_*) δ 164.80 (COO^−^), 149.77 (C_2_), 141.67 (C_5_), 136.20 (C_5’_), 131.04 (C_8_), 130.74 (C_9_), 130.17 (C_10_), 128.71 (C_6_, C_3_), 125.45 (C_4_), 119.01 (CN), 112.70 (C_7_) ppm. FT-IR (ATR) ν = 2235, 1578, 1537, 1450, 1397, 1335, 811, 789, 767, 682, 675 cm^−1^. Anal. calcd. for C_12_H_6_NNaO_2_S: C, 57.37; H, 2.41; N, 5.58; S, 12.76. Found: C, 57.48; H, 2.45; N, 5.61; S, 12.83.

***5-(4-Nitrophenyl)thiophene-2-carboxylic acid (1b).*** The compound was obtained according to Procedure A (SM). Starting compound: methyl 5-(4-nitrophenyl)thiophene-2-carboxylate. Yield: 98%. Aspect: yellow solid. Mp: 189 °C. TLC (DCM-MeOH 9:1): R_f_ = 0.20. The following analytical data are referred to the sodium salt. ^1^H NMR (300 MHz, DMSO-*d_6_*) δ 8.21 (d, *J* = 9.0 Hz, 2H, H_7,7’_), 7.97 (d, *J* = 9.0 Hz, 2H, H_6,6’_), 7.57 (d, *J* = 3.7 Hz, 1H, H_3_), 7.22 (d, *J* = 3.7 Hz, 1H, H_4_) ppm. ^13^C NMR (75 MHz, DMSO-*d_6_*) δ 164.05 (COO^−^), 152.14 (C_2_), 146.32 (C_8_), 141.46 (C_5_), 141.24 (C_5’_), 128.78 (C_3_), 126.96 (C_4_), 126.15 (C_7,7’_), 124.86 (C_6,6’_) ppm. FT-IR (KBr) ν = 3435, 2920, 2550, 1927, 1664, 1622, 1514, 1450, 1365, 995, 833, 704 cm^−1^. ESI-MS (m/z) calcd for C_11_H_6_NNaO_4_S 270.99, found 204.71 [M-CO_2_Na]^−^. Anal. calcd. for C_11_H_6_NNaO_4_S: C, 48.71; H, 2.23; N, 5.16; S, 11.82. Found: C, 48.75; H, 2.27; N, 5.14; S, 11.71.

***Sodium 5-(3-cyanophenyl)thiazole-2-carboxylate (2a).*** The compound was obtained according to Procedure B (SM). Starting compound: ethyl 5-(3-cyanophenyl)thiazole-2-carboxylate. Yield: 86%. Aspect: white solid. Mp: >300 °C (dec.). ^1^H NMR (300 MHz, DMSO-*d_6_*) δ 8.20 (s, 1H, H_4_), 8.19 (t, *J* = 1.4 Hz, 1H, H_6_), 7.95 (ddd, *J* = 7.9, 1.9, 1.2, Hz, 1H, H_10_), 7.77 (dt, *J* = 7.7, 1.2, H_8_), 7.60 (dt, *J* = 7.9, 0.5 Hz, 1H, H_9_) ppm. ^13^C NMR (75 MHz, DMSO-*d_6_*) δ 173.60 (C_2_), 161.61 (COO^−^), 140.73 (C_4_), 138.39 (C_5_), 133.51 (C_5’_), 131.81 (C_8_), 131.51 (C_10_), 130.85 (C_9_), 129.86 (C_6_), 118.85 (CN), 112.81 (C_7_) ppm. FT-IR (ATR) ν = 3354, 2235, 1663, 1641, 1578, 1440, 1407, 1366, 1110, 866, 806, 796 cm^−1^. Anal. calcd. for C_11_H_5_N_2_NaO_2_S: C, 52.38; H, 2.00; N, 11.11; S, 12.71. Found: C, 52.51; H, 2.02; N, 11.09; S, 12.75.

***Sodium 5-(4-nitrophenyl)thiazole-2-carboxylate (2b).*** The compound was obtained according to Procedure A (SM). Starting compound: ethyl 5-(4-nitrophenyl)thiazole-2-carboxylate. Yield: 85%. Aspect: dark green solid. Mp: >300 °C (dec.). ^1^H NMR (300 MHz, DMSO-*d_6_*) δ 8.29 (s, 1H, H_4_), 8.23 (d, *J* = 6.0 Hz, 2H, H_7,7’_), 7.93 (d, *J* = 6.0 Hz, 2H, H_6,6’_) ppm. ^13^C NMR (75 MHz, DMSO-*d_6_*) δ 174.68 (C_2_), 161.40 (COO^−^), 146.99 (C_8_), 141.97 (C_4_), 138.75 (C_5’_), 138.37 (C_5_), 127.62 (C_7,7’_), 124.86 (C_6,6’_) ppm. FT-IR (ATR) ν = 3648, 3297, 3100, 2963, 1675, 1645, 1621, 1595, 1514, 1424, 1408, 1365, 1343, 1108, 847 cm^−1^. ESI-MS (m/z) calcd for C_10_H_5_N_2_NaO_4_S 272.21, found 205.78 [M-CO_2_Na]^−^. Anal. calcd. for C_10_H_5_N_2_NaO_4_S: C, 44.12; H, 1.85; N, 10.29; S, 11.78. Found: C, 44.31; H, 1.87; N, 10.34; S, 11.67.

***Sodium 5-(3-cyanophenyl)oxazole-2-carboxylate (3a).*** The compound was obtained according to Procedure B (SM). Starting compound: ethyl 5-(3-cyanophenyl)oxazole-2-carboxylate. Yield: 89%. Aspect: grey solid. Mp: >300 °C (dec.). ^1^H NMR (300 MHz, DMSO-*d_6_*) δ 8.17 (t, *J* = 1.6 Hz, H_6_), 7.99 (dt, *J* = 8.0, 1.6 Hz, 1H, H_10_), 7.74 (dt, *J* = 8.0, 1.6 Hz, 1H, H_8_), 7.66 (t, *J* = 8.0 Hz, H_9_) ppm. ^13^C NMR (75 MHz, DMSO-*d_6_*) δ 161.87 (COO^−^), 157.88 (C_2_), 148.06 (C_5_), 131.96 (C_9_), 130.85 (C_8_), 129.69 (C_5’_), 128.69 (C_10_), 127.90 (C_6)_, 125.07 (C_4_), 118.78 (CN), 112.74 (C_7_) ppm. FT-IR (ATR) ν = 3522, 3383, 2234, 1650, 1616, 1520, 1422, 1389, 1318, 1273, 1216, 965, 825, 817, 795 cm^−1^. ESI-MS (*m*/*z*) calcd. for C_11_H_5_N_2_NaO_3_ 236.16, found 169.67 [M-CO_2_Na]^−^. Anal. calcd. for C_11_H_5_N_2_NaO_3_: C, 55.94; H, 2.13; N, 11.86. Found: C, 56.03; H, 2.15; N, 11.93.

***Sodium 5-(4-nitrophenyl)oxazole-2-carboxylate (3b).*** The compound was obtained according to Procedure B (SM). Starting compound: ethyl 5-(4-nitrophenyl)oxazole-2-carboxylate. Yield: 80%. Aspect: pale yellow solid. Mp: >300 °C (dec.). ^1^H NMR (300 MHz, DMSO-*d_6_*) δ 8.30 (d, *J* = 8.9 Hz, 2H, H_7,7’_), 7.96 (d, *J* = 6.0 Hz, 2H, H_6,6’_), 7.87 (s, 1H, H_4_) ppm. ^13^C APT NMR (75 MHz, DMSO-*d_6_*) δ 162.28 (COO^−^), 157.95 (C_2_), 148.37 (C_5_), 147.06 (C_8_), 134.32 (C_5’_), 126.99 (C_4_), 125.31 (C_7,7’_), 124.92 (C_6,6’_) ppm. FT-IR (ATR) ν = 3436, 2964, 1645, 1607, 1512, 1388, 1346, 1261, 1108, 854, 818 cm^−1^. ESI-MS (*m*/*z*) calcd. for C_10_H_5_N_2_NaO_5_ 256.15, found 189.94 [M-CO_2_Na]^−^. Anal. calcd. for C_10_H_5_N_2_NaO_5_: C, 46.89; H, 1.97; N, 10.94. Found: C, 46.59; H, 1.98; N, 10.92.

***5-(3-Cyanophenyl)-1H-imidazole-2-carboxylic acid (4a).*** The compound was synthesized through a specific procedure, reported in SM. Aspect: white solid. Mp: 165 °C. TLC (DCM-MeOH 7:3): R_f_ = 0.42. ^1^H NMR (300 MHz, DMSO-*d_6_*) δ 12.0-9.0 (broad s exch. D_2_O, 2H, NH_2_^+^), 8.25 (s, 1H, H_6_), 8.16 (d, *J* = 7.8 Hz, 1H, H_8_), 7.99 (s, 1H, H_4_), 7.68 (d, *J* = 7.8 Hz, 1H, H_10_), 7.58 (t, *J* = 7.8 Hz, 1H, H_9_) ppm. The compound degrades in solution at room temperature, during the acquisition of the ^13^C NMR spectrum. FT-IR (ATR) ν = 3205, 2228, 1666, 1601, 1514, 1473, 1426, 1403, 1334, 1130, 1089, 811, 780, 680 cm^−1^. ESI-MS (m/z) calcd. for C_11_H_7_N_3_O_2_ 213.19, found 212.42 [M-H]^−^. Anal. calcd. for C_11_H_7_N_3_O_2_: C, 61.97; H, 3.31; N, 19.71. Found: C, 62.03; H, 3.35; N, 19.82.

***5-(4-Nitrophenyl)-1H-imidazole-2-carboxylic acid (4b).*** The compound was obtained according to Procedure C (SM). Starting compound: ethyl 5-(4-nitrophenyl)-1*H*-imidazole-2-carboxylate. Yield: 66%. Aspect: red solid. Mp: 137 °C. TLC (DCM-MeOH 7:3): R_f_ = 0.40. The following analytical data are referred to the sodium salt. ^1^H NMR (300 MHz, DMSO-*d_6_*) δ 8.15-8.09 (m, 4H, H_6,6’_, H_7,7’_), 7.76 (s, 1H, H_4_) ppm. ^13^C NMR (75 MHz, DMSO-*d_6_*) δ 162.09 (COO^−^), 149.48 (C_2_), 145.46 (C_8_), 142.17 (C_5_), 137.58 (C_5’_), 125.45 (C_7,7’_), 124.19 (C_6,6’_), 117.67 (C_4_) ppm. FT-IR (ATR) ν = 3607, 3156, 1652, 1600, 1494, 1472, 1415, 1342, 1135, 1112, 992, 849, 751 cm^−1^. ESI-MS (m/z) calcd for C_10_H_7_N_3_O_4_ 233.18, found 232.32 [M-H]^−^. Anal. calcd. for C_10_H_6_N_3_NaO_4_: C, 47.07; H, 2.37; N, 16.47. Found: C, 47.31; H, 2.39; N, 16.36.

***5-(3-Cyanophenyl)-1,3,4-oxadiazole-2-carboxylic acid (5a).*** The compound was obtained according to Procedure D (SM). Starting compound: ethyl 5-(3-cyanophenyl)-1,3,4-oxadiazole-2-carboxylate. Yield: quantitative. Aspect: white solid. Mp: 222 °C (dec.). TLC (DCM-MeOH 7:3): R_f_ = 0.42. The following analytical data are referred to the sodium salt. ^1^H NMR (300 MHz, DMSO-*d_6_*) δ 8.35 (t, *J* = 1.8 Hz, 1H, H_6_), 8.28 (dt, *J* = 8.0, 1.4 Hz, 1H, H_8_), 8.06 (dt, *J* = 7.8, 1.4 Hz, 1H, H_10_), 7.79 (dt, *J* = 7.9, 0.7 Hz, 1H, H_9_) ppm. The compound degrades in solution at room temperature, during the acquisition of the ^13^C NMR spectrum. FT-IR (ATR) ν = 3543, 3384, 2232, 1651, 1614, 1549, 1400, 1343, 1228, 1183, 1086, 812, 807, 679 cm^−1^. Anal. calcd. for C_10_H_4_N_3_NaO_3_: C, 50.65; H, 1.70; N, 17.72. Found: C, 50.71; H, 1.81; N, 17.87.

***5-(4-Nitrophenyl)-1,3,4-oxadiazole-2-carboxylic acid (5b).*** The compound was obtained according to Procedure D (SM). Starting compound: ethyl 5-(4-nitrophenyl)-1,3,4-oxadiazole-2-carboxylate. Yield: quantitative. Aspect: pale yellow solid. Mp: 220 °C (dec.). TLC (DCM-MeOH 7:3): R_f_ = 0.44. ^1^H NMR (300 MHz, DMSO-*d_6_*) δ 13.7 (broad s exch. D_2_O, 1H, COOH), 8.30 (d, *J* = 8.9 Hz, 2H, H_7,7’_), 8.15 (d, *J* = 8.9 Hz, 2H, H_6,6’_) ppm. ^13^C NMR (75 MHz, DMSO-*d_6_*) δ 166.24 (COOH), 150.51 (C_5_), 136.98 (C_5’_), 131.14 (C_7,7’_), 124.15 (C_6,6’_) ppm. FT-IR (ATR) ν = 2962, 2924, 2853, 1691, 1603, 1520, 1258, 1080, 1013, 789 cm^−1^. Anal. calcd. for C_9_H_5_N_3_O_5_: C, 45.97; H, 2.14; N, 17.87. Found: C, 46.02; H, 2.17; N, 17.96.

***1-(3-Cyanophenyl)-1H-1,2,3-triazole-4-carboxylic acid (6a).*** The compound was synthesized through a specific procedure, reported in SM. Aspect: white solid. TLC (DCM-MeOH 9:1): R_f_ = 0.14. ^1^H NMR (300 MHz, DMSO-*d_6_*) δ 8.90 (s, 1H, H_1_), 8.44 (t, *J* = 2.0 Hz, 1H, H_6_), 8.30 (ddd, *J* = 1.2, 2.0, 8.1 Hz, 1H, H_8_), 7.91 (dt, *J* = 1.2, 8.1 Hz, 1H, H_10_), 7.77 (t, *J* = 8.1 Hz, 1H, H_9_) ppm. ^13^C NMR (75 MHz, DMSO-*d_6_*) δ 177.11 (C_2_), 163.73 (COOH), 137.81 (C_5’_), 132.35 (C_8_), 131.66 (C_9_), 124.99 (C_10_), 124.28 (C_6_), 123.68 (C_1_), 118.30 (CN), 113.25 (C_7_) ppm. FT-IR (ATR) ν = 3389, 3091, 2235, 1589, 1557, 1536, 1403, 1343, 1312, 1021, 794 cm^−1^. Anal. calcd. for C_10_H_6_N_4_O_2_: C, 56.08; H, 2.82; N, 26.16. Found: C, 56.27; H, 2.91; N, 26.35.

***1-(4-Nitrophenyl)-1H-1,2,3-triazole-4-carboxylic acid (6b).*** The compound was obtained according to Procedure A (SM). Starting compound: ethyl 1-(4-nitrophenyl)-1*H*-1,2,3-triazole-4-carboxylate. Yield: 91%. Aspect: white solid. Mp: 175 °C. TLC (DCM-MeOH 8:2): R_f_ = 0.12. ^1^H NMR (300 MHz, DMSO-*d_6_*) δ 13.4 (broad s exch. D_2_O, 1 H, COOH), 9.59 (s, 1H, H_1_), 8.46 (d, *J* = 7.0 Hz, 2H, H_7,7’_), 8.30 (d, *J* = 7.0 Hz, 2H, H_6,6’_) ppm. ^13^C NMR (75 MHz, DMSO-*d_6_*) δ 161.73 (COOH), 147.60 (C_8_), 141.69 (C_2_), 140.96 (C_5’_), 128.02 (C_1_), 125.90 (C_7,7’_), 121.72 (C_6,6’_) ppm. FT-IR (ATR) ν = 3249, 3142, 3102, 3061, 2962, 2913, 2866, 1729, 1704, 1596, 1516, 1342, 1268, 1219, 1151, 1032, 982, 869, 854 cm^−1^. ESI-MS (m/z) calcd for C_9_H_6_N_4_O_4_ 234.17, found 161.30 [M-CO_2_-N_2_]^−^. Anal. calcd. for C_9_H_6_N_4_O_4_: C, 46.16; H, 2.58; N, 23.93. Found: C, 46.25; H, 2.60; N, 23.97.

***5-(3-Cyano-5-(trifluoromethyl)phenyl)furan-2-carboxylic acid (IV).*** The compound was synthesized according to a previously published procedure [13]. Yield: 70%. Aspect: white solid. Mp: 210 °C. TLC (DCM-MeOH 7:3): R_f_ = 0.33. ^1^H NMR (300 MHz, DMSO-*d_6_*) δ 13.59-13.10 (broad s. exch. D_2_O, 1H, COOH), 8.62-8.57 (m, 1H, H_7_), 8.41-8.33 (m, 2H, H_11_, H_9_), 7.52 (d, *J* = 3.7 Hz, 1H, H_4_), 7.38 (d, *J* = 3.7 Hz, 1H, H_3_) ppm. ^13^C APT NMR (75 MHz, DMSO-*d_6_*) δ 159.45 (COOH), 152.84 (C_5_), 146.18 (C_2_), 132.17 (C_7_), 132.17-131.78-131.34-130.90 (q, C_10_), 131.99 (C_6_), 129.14 (C_9_), 128.80-125.19-121.56-117.94 (q, CF_3_), 125.09 (C_11_), 120.09 (C_3_), 117.53 (CN), 114.28 (C_8_), 111.74 (C_4_) ppm. FT-IR (ATR): ν = 3130, 3082, 2960, 2917, 2849, 2237, 1688, 1578, 1519, 1455, 1438, 1410, 1344, 1274, 1256, 1163, 1133, 1114, 1085, 1032, 900 cm^−1^. ESI-MS (m/z) calcd for C_13_H_6_F_3_NO_3_ 281.03, found 280.14 [M-H]^−^. Anal. calcd. for C_13_H_6_F_3_NO_3_: C, 55.53; H, 2.15; N, 4.98. Found: C, 55.47; H, 2.19; N, 5.01.

***5-(3,5-Bis(trifluoromethyl)phenyl)furan-2-carboxylic acid (V).*** The compound was synthesized according to a previously published procedure [13]. Yield: 91%. Aspect: white solid. Mp: 168 °C. TLC (DCM-MeOH 7:3): R_f_ = 0.39. ^1^H NMR (300 MHz, DMSO-*d_6_*) δ 13.65-13.20 (broad s. exch. D_2_O, 1H, COOH), 8.41-8.36 (m, 2H, H_7_, H_11_), 8.12-8.07 (m, 1H, H_9_), 7.59 (d, *J* = 3.7 Hz, 1H, H_3_), 7.38 (d, *J* = 3.7 Hz, 1H, H_4_) ppm. ^13^C APT NMR (75 MHz, DMSO-*d_6_*) δ 159.48 (COOH), 153.19 (C_5_), 146.03 (C_2_), 132.34-131.90-131.46-131.03 (q, C_8_), 132.00 (C_6_), 128.98-125.36-121.74-118.12 (q, CF_3_), 125.00 (C_11_), 122.14 (C_9_), 120.14 (C_3_), 111.73 (C_4_) ppm. FT-IR (ATR): ν = 2960, 2925, 2855, 1689, 1621, 1591, 1526, 1455, 1420, 1366, 1278, 1161, 1124, 1081, 1027, 896 cm^−1^. ESI-MS (m/z) calcd for C_13_H_6_F_6_O_6_ 324.18, found 323.16 [M-H]^−^. Anal. calcd. for C_13_H_6_F_6_O_3_: C, 48.17; H, 1.87. Found: C, 48.02; H, 1.91.

***5-(3-cyano-5-fluorophenyl)furan-2-carboxylic acid (VI).*** The compound was synthesized according to a previously published procedure [13]. Yield: quantitative. Aspect: white solid. Mp: 247 °C. TLC (DCM-MeOH 7:3): R_f_ = 0.26. ^1^H NMR (300 MHz, DMSO-*d_6_*) δ 13.38 (broad s exch D_2_O, 1H, COOH), 8.14 (t, *J* = 1.6, 1H, H_7_), 7.96 (dd, *J* = 7.8, 1.6 Hz, 1H, H_11_), 7.88 (dd, *J* = 6.0, 1.6 Hz, 1H, H_9_), 7.43 (d, *J* = 3.7 Hz, 1H, H_4_), 7.37 (d, *J* = 3.7 Hz, 1H, H_3_) ppm. ^13^C APT NMR (75 MHz, DMSO-*d_6_*) δ 164.18-160.91 (d, CF), 159.53 (COOH), 153.20-153.16 (d, C_5_), 145.89 (C_2_), 133.05-132.93 (d, C_6_) 124.92-124.87 (d, C_7_), 120.19 (C_3_), 119.75-119.41 (d, C_11_), 117.72-117.68 (d, CN), 116.57-116.25 (d, C_9_), 114.46-114.32 (d, C_8_), 111.43 (C_4_) ppm. FT-IR (ATR): ν = 3113, 2916, 2849, 2663, 2575, 2231, 1675, 1594, 1519, 1435, 1308, 1214, 1173, 1028, 866, 809, 760 cm^−1^. ESI-MS (m/z) calcd for C_12_H_6_FNO_3_ 231.18, found 230.50 [M-H]^−^. Anal. calcd. for C_12_H_6_F NO_3_: C, 62.34; H, 2.62. Found: C, 62.53; H, 2.51.

***5-(3-Cyano-5-methoxyphenyl)furan-2-carboxylic acid (VII).*** The compound was synthesized according to a previously published procedure [13]. Yield: 80%. Aspect: white solid. Mp: 226 °C. TLC (DCM-MeOH 7:3): R_f_ = 0.46. ^1^H NMR (300 MHz, DMSO-*d_6_*) δ 13.42-13.19 (broad s. exch. D_2_O, 1H, COOH), 7.82 (t, *J* = 1.4 Hz, 1H, H_7_), 7.59 (dd, *J* = 2.5, 1.5 Hz, 1H, H_11_), 7.45 (dd, *J* = 2.5, 1.3 Hz, 1H, H_9_), 7.35 (d, *J* = 3.7 Hz, 1H, H_3_), 7.33 (d, *J* = 3.7 Hz, 1H, H_4_), 3.87 (s, 3H, CH_3_) ppm. ^13^C NMR (75 MHz, DMSO-*d_6_*) δ 160.43 (C_10_) 159.61 (COOH), 154.2 (C_5_), 145.46 (C_2_), 132.08 (C_6_), 120.73 (C_3_), 120.20 (C_7_), 118.66 (CN), 117.65 (C_9_), 115.02 (C_11_), 113.72 (C_8_), 110.60 (C_4_), 56.49 (CH_3_) ppm. FT-IR (ATR): ν = 3116, 3086, 2926, 2574, 2229, 1693, 1608, 1594, 1572, 1515, 1461, 1427, 1305, 1215, 1167, 1033, 960 cm^−1^. ESI-MS (*m*/*z*) calcd for C_13_H_9_O_4_ 243.05, found 242.28 [M-H]^−^. Anal. calcd. for C_13_H_9_F_3_O_4_: C, 54.56; H, 3.17. Found: C, 54.71; H, 3.23.

***5-(3-cyano-5-methylphenyl)furan-2-carboxylic acid (VIII).*** The compound was synthesized according to a previously published procedure [13]. Yield: 90%. Aspect: white solid. Mp: 238 °C. TLC (DCM-MeOH 7:3): R_f_ = 0.31 ^1^H NMR (300 MHz, DMSO-*d_6_*) δ 13.20 (broad s exch D_2_O, 1H, COOH), 8.08-8.02 (m, 1H, H_7_), 7.93-7.88 (m, 1H, H_11_), 7.67-7.62 (m, 1H, H_9_), 7.33 (d, *J* = 3.6 Hz, 1H, H_3_), 7.28 (d, *J* = 3.6 Hz, 1H, H_4_), 2.39 (s, 3H, CH_3_) ppm. ^13^C NMR (75 MHz, DMSO-*d_6_*) δ 159.56 (COOH), 154.49 (C_2_), 145.39 (C_5_), 140.84 (C_10_), 132.82 (C_9_), 130.66 (C_6_), 129.51 (C_11_), 125.61 (C_7_), 120.13 (C_3_), 118.82 (CN), 112.64 (C_8_), 110.06 (C_4_), 20.89 (CH_3_) ppm. FT-IR (ATR): ν = 3119, 2926, 2849, 2692, 2579, 2228, 1726, 1682, 1584, 1520, 1422, 1299, 1169, 1029, 858, 810, 760 cm^−1^. ESI-MS (m/z) calcd for C_13_H_9_NO_3_ 227.063, found 226.35 [M-H]^−^. Anal. calcd. for C_13_H_9_ NO_3_: C, 68.72; H, 3.99. Found: C, 68.93; H, 4.01.

***5-(3-cyano-5-hydroxyphenyl)furan-2-carboxylic acid (IX).*** The compound was synthesized according to a previously published procedure [13]. Yield: 95%. Aspect: white solid. Mp: 280 °C (dec.). TLC (DCM-MeOH 7:3 and 3 drops of CH_3_COOH): R_f_ = 0.44. ^1^H NMR (300 MHz, DMSO-*d_6_*) δ 13.22 (broad s exch. D_2_O, 1H, COOH), 10.54 (broad s exch. D_2_O, 1H, OH), 7.71 (t, *J* = 1.5, 1H, H_7_), 7.48 (dd, *J* = 2.4, 1.5 Hz, 1H, H_11_), 7.32 (d, *J* = 3.6 Hz, 1H, H_3_), 7.26 (d, *J* = 3.6 Hz, 1H, H_4_), 7.14 (dd, *J* = 2.4, 1.5, 1H, H_9_) ppm. ^13^C APT NMR (75 MHz, DMSO-*d_6_*) δ 159.50 (C_10_), 158.90 (COOH), 154.42 (C_5_), 145.27 (C_2_), 132.09 (C_6_), 120.10 (C_3_), 119.32 (C_7_), 118.93 (C_9_), 118.74 (CN), 115.92 (C_11_), 113.50 (C_8_), 110.08 (C_4_) ppm. FT-IR (ATR): ν = 3400, 3108, 2602, 2228, 1652, 1595, 1516, 1487, 1439, 1310, 1241, 1213, 1174, 1152, 1035, 963, 881, 816, 668 cm^−1^. ESI-MS (m/z) calcd. for C_12_H_7_NO_4_ 229.19, found 228.29 [M-H]^−^. Anal. calcd. for C_12_H_7_ NO_4_: C, 62.89; H, 3.08. Found: C, 62.53; H, 3.05.

### 4.2. Biological Activities

#### 4.2.1. MbtI Enzymatic Assays

Recombinant *M. tuberculosis* MbtI was produced and purified as previously reported [14]. Enzyme activity was determined at 37 °C, measuring the formation of salicylic acid by a fluorimetric assay, slightly modified from Vasan et al. [38]. Briefly, the reactions were performed in a final volume of 400 μL of 50 mM Hepes pH 7.5, 5 mM MgCl_2_, containing 1-2 μM MbtI, by the addition of chorismic acid, and monitored using a Perkin-Elmer LS3 fluorimeter (Ex. λ = 305 nm, Em. λ = 420 nm). Inhibition assays were performed in the presence of the compound at 100 µM (stock solution 20 mM in DMSO) and 50 µM chorismic acid. Where possible, compounds were tested both as free acids and sodium salts, providing analogous results. For compounds inhibiting by more than 75% the initial activity, IC_50_ values were determined. To this end, the activity was measured at different compound concentrations, and values were calculated according to Equation (1), with Origin 8 software:

(1) $$A_{[I]\;}{\;=\;A}_{\left[0\right]}\;\times \;\left(1\;-\frac{\left[I\right]}{{[I]\;+\;\mathrm{IC}}_{50}}\right)$$

where A_[I]_ is the activity at inhibitor concentration [I] and A[0] is the activity in the absence of the inhibitor.

The K_i_ was determined at different substrate [S] and compound concentrations, using Equation (2):

(2) $$v\;=\;\frac{V_{\max}\left[S\right]}{\left[S\right]{\;+\;K}_{m\;}\left(1\;+\;\frac{\left[I\right]}{K_{i}}\right)}$$

#### 4.2.2. MIC Determination

The minimal inhibitory concentration (MIC^99^) of the most active compounds was determined against *M. bovis* BCG in low-iron chelated Sauton’s medium, by the 2-fold microdilution method in U-bottom 96-well microtiter plates, as previously reported [13]. To this purpose, cells were grown in 7H9 medium, sub-cultured in chelated Sauton’s medium, and then diluted to an OD_600_ of 0.01 in chelated Sauton’s containing different concentrations of the test compound. After 15 days of incubation at 37 °C, the growth was evaluated by the resazurin reduction assay method (REMA). Thirty microliters of a 0.01% solution of filter-sterilized resazurin sodium salt were added to each well, and the microtiters were re-incubated at the same temperature for 24 h. The MIC was defined as the lowest concentration of the drug that prevented a change in color from blue to pink, which indicates bacterial growth.

## 5. Conclusions

In this paper, a SAR study on our series of MbtI inhibitors led to the identification of new candidates, endowed with a potent activity against the enzyme and encouraging bactericidal properties. For the new products, we described the design, synthesis, analytical characterization, and biological activity.

Firstly, two sets of compounds, series A and B, incorporating a variety of heterocyclic motifs were biologically evaluated against MbtI and in whole-cell assays against *M. bovis* BCG. This approach led to the disclosure of **1b, 3b**, and **4b** provided with a moderate activity (IC_50_ in the range 18–27 µM), but low bactericidal effects. Overall, the obtained results confirmed that the furan core was a better scaffold to gain MbtI inhibition in comparison with several other heterocycles.

These findings provided the basis for the design of the new furan-based derivatives **IV**–**IX**, which were synthesized, characterized, and tested. The best compound of this series, **IV**, bearing the preferred cyano group at position 3 and a trifluoromethyl moiety in 5, showed a potent MbtI inhibitory effect (%RA ≈ 1%, IC_50_ ≈ 15 μM, K*i* ≈ 9 μM), comparable to the previous lead **I**, but exhibiting an enhanced antimycobacterial action (MIC^99^ = 125 μM), thus becoming one of the few potent MbtI inhibitors endowed with a promising antitubercular activity.

These observations justify the selection of **IV** as the new lead of our next optimization campaign, which will contribute to strengthen the perspectives of anti-TB drug discovery.

## Figures and Tables

**Table 1:** In vitro activity of compounds **1a,b**–**6a,b**.

Structure	Series A R = *m*-CNPh			Series B R = *p*-NO_2_Ph	
	%RA *	IC_50_ ** (µM)		%RA *	IC_50_ ** (µM)
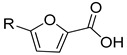	**I**			**II**	
	3.1 ± 1.0		6.3 ± 0.9	18.2 ± 5.1	7.6 ± 1.6
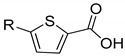	**1a**			**1b**	
	23.7 ± 5.0		35.5 ± 1.9	22.5 ±10.8	18.6 ± 1.7
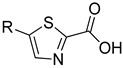	**2a**			**2b**	
	24.8 ± 1.8		41.9 ± 7.3	32.8 ± 2.3	-
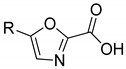	**3a**			**3b**	
	38.0 ± 7.6		-	23.0 ± 7.8	21.1 ± 2.7
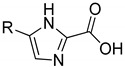	**4a**			**4b**	
	21.2 ± 1.0		39.3 ± 3.0	18.2 ± 4.9	27.5 ± 2.3
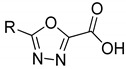	**5a**			**5b**	
	75.0 ± 9.8		-	59.0 ± 4.3	-
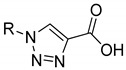	**6a**			**6b**	
	67.9 ± 6.6		-	32.7 ± 7.8	-

* % residual enzymatic activity at 100 µM; ** only for compounds with %RA ≤ 25%.

**Table 2:** In vitro activity of compounds **IV**–**IX**.

Entry	% RA *	IC_50_ ** (µM)
**IV**	0.7 ± 2.7	15.5 ± 3.1
**V**	8.9 ± 1.6	18.8 ± 6.8
**VI**	8.7 ± 1.4	17.3 ± 3.1
**VII**	10.5 ± 3.9	14.5 ± 2.1
**VIII**	16.7 ± 3.8	29.1 ± 1.8
**IX**	15.3 ± 2.6	33.5 ± 3.4

* % residual enzymatic activity at 100 µM; ** only for compounds with %RA ≤ 25%.

## Data Availability

The data presented in this study are available within the article.

## Supplementary Materials

The Supplementary Materials are available online at https://www.mdpi.com/1424-8247/14/2/155/s1.

## References

[1] (2020). Global Tuberculosis Report 2020.

[2] Xu K., Liang Z.C., Ding X., Hu H., Liu S., Nurmik M., Bi S., Hu F., Ji Z., Ren J. (2018). Nanomaterials in the Prevention, Diagnosis, and Treatment of *Mycobacterium Tuberculosis* Infections. Adv. Healthc. Mater..

[3] Truzzi E., Meneghetti F., Mori M., Costantino L., Iannuccelli V., Maretti E., Domenici F., Castellano C., Rogers S., Capocefalo A. (2019). Drugs/Lamellae Interface Influences the Inner Structure of Double-Loaded Liposomes for Inhaled Anti-TB Therapy: An In-Depth Small-Angle Neutron Scattering Investigation. J. Colloid Interface Sci..

[4] Nahid P., Dorman S.E., Alipanah N., Barry P.M., Brozek J.L., Cattamanchi A., Chaisson L.H., Chaisson R.E., Daley C.L., Grzemska M. (2016). Official American Thoracic Society/Centers for Disease Control and Prevention/Infectious Diseases Society of America Clinical Practice Guidelines: Treatment of Drug-Susceptible Tuberculosis. Clin. Infect. Dis..

[5] Falzon D., Jaramillo E., Schünemann H.J., Arentz M., Bauer M., Bayona J., Blanc L., Caminero J.A., Daley C.L., Duncombe C. (2011). WHO Guidelines for the Programmatic Management of Drug-Resistant Tuberculosis: 2011 Update. Eur. Respir. J..

[6] Fanzani L., Porta F., Meneghetti F., Villa S., Gelain A., Lucarelli A.P., Parisini E. (2015). *Mycobacterium tuberculosis* Low Molecular Weight Phosphatases (MPtpA and MPtpB): From Biological Insight to Inhibitors. Curr. Med. Chem..

[7] Stelitano G., Sammartino J.C., Chiarelli L.R. (2020). Multitargeting Compounds: A Promising Strategy to Overcome Multi-Drug Resistant Tuberculosis. Molecules.

[8] Mori M., Sammartino J.C., Costantino L., Gelain A., Meneghetti F., Villa S., Chiarelli L.R. (2019). An Overview on the Potential Antimycobacterial Agents Targeting Serine/Threonine Protein Kinases from *Mycobacterium tuberculosis*. Curr. Top. Med. Chem..

[9] Meneghetti F., Villa S., Gelain A., Barlocco D., Chiarelli L.R., Pasca M.R., Costantino L. (2016). Iron Acquisition Pathways as Targets for Antitubercular Drugs. Curr. Med. Chem..

[10] Chao A., Sieminski P.J., Owens C.P., Goulding C.W. (2019). Iron Acquisition in Mycobacterium tuberculosis. Chem. Rev..

[11] Zhang X.-K., Liu F., Fiers W.D., Sun W.-M., Guo J., Liu Z., Aldrich C.C. (2017). Synthesis of Transition-State Inhibitors of Chorismate Utilizing Enzymes from Bromobenzene *cis* -1,2-Dihydrodiol. J. Org. Chem..

[12] Mori M., Stelitano G., Gelain A., Pini E., Chiarelli L.R., Sammartino J.C., Poli G., Tuccinardi T., Beretta G., Porta A. (2020). Shedding X-ray Light on the Role of Magnesium in the Activity of *M. tuberculosis* Salicylate Synthase (MbtI) for Drug Design. J. Med. Chem..

[13] Chiarelli L.R., Mori M., Beretta G., Gelain A., Pini E., Sammartino J.C., Stelitano G., Barlocco D., Costantino L., Lapillo M. (2019). New Insight into Structure-Activity of Furan-based Salicylate Synthase (MbtI) Inhibitors as Potential Antitubercular Agents. J. Enzym. Inhib. Med. Chem..

[14] Chiarelli L.R., Mori M., Barlocco D., Beretta G., Gelain A., Pini E., Porcino M., Mori G., Stelitano G., Costantino L. (2018). Discovery and Development of Novel Salicylate Synthase (MbtI) Furanic Inhibitors as Antitubercular Agents. Eur. J. Med. Chem..

[15] Pini E., Poli G., Tuccinardi T., Chiarelli L., Mori M., Gelain A., Costantino L., Villa S., Meneghetti F., Barlocco D. (2018). New Chromane-Based Derivatives as Inhibitors of *Mycobacterium tuberculosis* Salicylate Synthase (MbtI): Preliminary Biological Evaluation and Molecular Modeling Studies. Molecules.

[16] Hinsberg O. (1916). Über β-Naphtolsulfid und Iso-β-naphtolsulfid. J. Prakt. Chem..

[17] Chauhan P.M.S., Sunduru N., Sharma M. (2010). Recent Advances in the Design and Synthesis of Heterocycles as Anti-Tubercular Agents. Future Med. Chem..

[18] Singh P., Kumar S.K., Maurya V.K., Mehta B.K., Ahmad H., Dwivedi A.K., Chaturvedi V., Thakur T.S., Sinha S. (2017). S-Enantiomer of the Antitubercular Compound S006-830 Complements Activity of Frontline TB Drugs and Targets Biogenesis of *Mycobacterium tuberculosis* Cell Envelope. ACS Omega.

[19] Aggarwal A., Parai M.K., Shetty N., Wallis D., Woolhiser L., Hastings C., Dutta N.K., Galaviz S., Dhakal R.C., Shrestha R. (2017). Development of a Novel Lead that Targets *M. tuberculosis* Polyketide Synthase 13. Cell.

[20] Wilson R., Kumar P., Parashar V., Vilchèze C., Veyron-Churlet R., Freundlich J.S., Barnes S.W., Walker J.R., Szymonifka M.J., Marchiano E. (2013). Antituberculosis Thiophenes Define a Requirement for Pks13 in Mycolic Acid Biosynthesis. Nat. Chem. Biol..

[21] Nayak S., Gaonkar S.L. (2018). A Review on Recent Synthetic Strategies and Pharmacological Importance of 1,3-Thiazole Derivatives. Mini Rev. Med. Chem..

[22] Machado D., Azzali E., Couto I., Costantino G., Pieroni M., Viveiros M. (2018). Adjuvant Therapies Against Tuberculosis: Discovery of a 2-Aminothiazole Targeting Mycobacterium tuberculosis Energetics. Future Microbiol..

[23] Azzali E., Machado D., Kaushik A., Vacondio F., Flisi S., Cabassi C.S., Lamichhane G., Viveiros M., Costantino G., Pieroni M. (2017). Substituted N-Phenyl-5-(2-(phenylamino)thiazol-4-yl)isoxazole-3-carboxamides Are Valuable Antitubercular Candidates that Evade Innate Efflux Machinery. J. Med. Chem..

[24] Tyagi S., Nuermberger E., Yoshimatsu T., Williams K., Rosenthal I., Lounis N., Bishai W., Grosset J. (2005). Bactericidal Activity of the Nitroimidazopyran PA-824 in a Murine Model of Tuberculosis. Antimicrob. Agents Chemother..

[25] Pieroni M., Wan B., Zuliani V., Franzblau S.G., Costantino G., Rivara M. (2015). Discovery of Antitubercular 2,4-diphenyl-1H-imidazoles from Chemical Library Repositioning and Rational Design. Eur. J. Med. Chem..

[26] De S.S., Khambete M.P., Degani M.S. (2019). Oxadiazole Scaffolds in Anti-Tuberculosis Drug Discovery. Bioorg. Med. Chem. Lett..

[27] Zhou B., He Y., Zhang X., Xu J., Luo Y., Wang Y., Franzblau S.G., Yang Z., Chan R.J., Liu Y. (2010). Targeting Mycobacterium Protein Tyrosine Phosphatase B for Antituberculosis Agents. Proc. Natl. Acad. Sci. USA.

[28] Kadam K.S., Jadhav R.D., Kandre S., Guha T., Reddy M.M.K., Brahma M.K., Deshmukh N.J., Dixit A., Doshi L., Srinivasan S. (2013). Evaluation of Thiazole Containing Biaryl Analogs as Diacylglycerol Acyltransferase 1 (DGAT1) Inhibitors. Eur. J. Med. Chem..

[29] Wu X., Geng X., Zhao P., Zhang J., Wu Y.D., Wu A.X. (2017). I2-Promoted Formal [3+2] Cycloaddition of α-Methylenyl Isocyanides with Methyl Ketones: A Route to 2,5-Disubstituted Oxazoles. Chem. Commun..

[30] Curreli F., Kwon Y.D., Belov D.S., Ramesh R.R., Kurkin A.V., Altieri A., Kwong P.D., Debnath A.K. (2017). Synthesis, Antiviral Potency, in Vitro ADMET, and X-ray Structure of Potent CD4 Mimics as Entry Inhibitors That Target the Phe43 Cavity of HIV-1 gp120. J. Med. Chem..

[31] Leung D., Du W., Hardouin C., Cheng H., Hwang I., Cravatt B.F., Boger D.L. (2005). Discovery of an Exceptionally Potent and Selective Class of Fatty Acid Amide Hydrolase Inhibitors Enlisting Proteome-Wide Selectivity Screening: Concurrent Optimization of Enzyme Inhibitor Potency and Selectivity. Bioorg. Med. Chem. Lett..

[32] Aksenov A.V., Khamraev V., Aksenov N.A., Kirilov N.K., Domenyuk D.A., Zelensky V.A., Rubin M. (2019). Electrophilic Activation of Nitroalkanes in Efficient Synthesis of 1,3,4-Oxadiazoles. RSC Adv..

[33] Devine S.M., Mulcair M.D., Debono C.O., Leung E.W.W., Nissink J.W.M., Lim S.S., Chandrashekaran I.R., Vazirani M., Mohanty B., Simpson J.S. (2015). Promiscuous 2-Aminothiazoles (PrATs): A Frequent Hitting Scaffold. J. Med. Chem..

[34] Lagorce D., Bouslama L., Becot J., Miteva M.A., Villoutreix B.O. (2017). FAF-Drugs4: Free ADME-tox Filtering Computations for Chemical Biology and Early Stages Drug Discovery. Bioinformatics.

[35] Daina A., Michielin O., Zoete V. (2017). SwissADME: A Free WebTool to Evaluate Pharmacokinetics, Drug-Likeness and Medicinal Chemistry Friendliness of Small Molecules. Sci. Rep..

[36] Chapman T.M., Bouloc N., Buxton R.S., Chugh J., Lougheed K.E.A., Osborne S.A., Saxty B., Smerdon S.J., Taylor D.L., Whalley D. (2012). Substituted Aminopyrimidine Protein Kinase B (PknB) Inhibitors Show Activity Against *Mycobacterium tuberculosis*. Bioorg. Med. Chem. Lett..

[37] Shyam M., Shilkar D., Verma H., Dev A., Sinha B.N., Brucoli F., Bhakta S., Jayaprakash V. (2020). The Mycobactin Biosynthesis Pathway: A Prospective Therapeutic Target in the Battle against Tuberculosis. J. Med. Chem..

[38] Vasan M., Neres J., Williams J., Wilson D.J., Teitelbaum A.M., Remmel R.P., Aldrich C.C. (2010). Inhibitors of the Salicylate Synthase (MbtI) from *Mycobacterium tuberculosis* Discovered by High-Throughput Screening. ChemMedChem.

