# Supplementary material for: Synthesis, Characterization, and Biological Evaluation of New Derivatives Targeting MbtI as Antitubercular Agents
Source: Pharmaceuticals (Basel). 2021 Feb 13;14(2):155. doi: 10.3390/ph14020155 (PMC7918538; doi:10.3390/ph14020155)
Supplement: Supplementary file 1 [file pharmaceuticals-14-00155-s001.pdf]

Synthesis, characterization, and biological evaluation of new derivatives targeting MbtI as antitubercular agents

Matteo Mori [1,‡], Giovanni Stelitano [2,‡], Laurent R. Chiarelli [2], Giulia Cazzaniga [1], Arianna Gelain [1], Daniela Barlocco [1], Elena Pini [1], Fiorella Meneghetti [1,*] and Stefania Villa [1]

[1] Department of Pharmaceutical Sciences, University of Milan, via L. Mangiagalli 25, 20133 Milan, Italy; matteo.mori@unimi.it; giulia.cazzaniga@unimi.it; arianna.gelain@unimi.it; daniela.barlocco@unimi.it; elena.pini@unimi.it; stefania.villa@unimi.it

[2] Department of Biology and Biotechnology "Lazzaro Spallanzani", University of Pavia, via A. Ferrata 9, 27100 Pavia; Italy.; laurent.chiarelli@unipv.it; giovanni.stelitano01@universitadipavia.it

* Correspondence: fiorella.meneghetti@unimi.it; Tel.: +39-02-50319306

Supplementary Materials

Chemistry - Synthetic procedures……………………………………………………………………………….S2
Chemistry – Experimental data………………………………………………………………………………S12

Chemistry – Synthetic procedures

Procedure A. The appropriate ester (1.0 mmol) was dissolved in a mixture of EtOH-THF 1:1 (15 mL). A 1 M NaOH solution (2.5 mmol) was added dropwise, and the reaction mixture was heated at reflux for 5 h. THF and EtOH were evaporated under reduced pressure, the aqueous phase was washed with DCM (5 mL), acidified with 1 M HCl and, eventually, extracted with EtOAc (3 × 5 mL). The combined organic layers were dried over anhydrous Na_2SO_4 and concentrated *in vacuo* to give the desired acid. The product was converted to its sodium salt, using equimolar amounts of aqueous $NaHCO_3$, followed by freeze-drying.

Procedure B. Pellets of NaOH (0.11 mmol) were dissolved in H_2O (2 mL) and added to a solution of the appropriate ester (0.11 mmol) in THF (2 mL). The reaction mixture was stirred at room temperature for 1.5 h; then, the solvent was removed under reduced pressure, and the aqueous layer was freeze-dried to afford the final product as a sodium salt.

Procedure C. The appropriate ester (1.0 mmol) was dissolved in THF (5 mL), and a solution of NaOH (5.0 mmol) in H_2O (5 mL) was added dropwise while stirring; the resulting mixture was heated at reflux for 6 h. After completion, the reaction was acidified with 3 M HCl until the formation of a precipitate, which was filtered to afford the desired free acid. The product was converted to its sodium salt, using equimolar amounts of aqueous $NaHCO_3$, followed by freeze-drying.

Procedure D. To a solution of the appropriate ester (1.0 mmol) in THF-H_2O 1:1, LiOH (or NaOH) (1.5 mmol) was added and the resulting solution was stirred at room temperature for 1 h. After completion, THF was evaporated under reduced pressure and the mixture was extracted with EtOAc (3 × 5 mL). The aqueous phase was treated with Amberlite IR120 and freeze-dried, giving the desired product. It is advisable to avoid acidification with mineral acids (even at low temperatures) to prevent the decarboxylation of the 1,3,4-oxadiazole derivative.

Procedure E. To a solution of the appropriate carboxylic acid (9.66 mmol) in MeOH or EtOH (65 mL), concentrated H_2SO_4 (1.5 mL) was added dropwise at room temperature. Then, the reaction mixture was heated at reflux overnight. After cooling to room temperature, the solvent was removed under reduced pressure. The residue was diluted with H_2O (20 mL) and basified with a saturated solution of $NaHCO_3$. The mixture was extracted with EtOAc (3 × 20 mL); the organic layers were combined and dried over anhydrous Na_2SO_4, filtered, and concentrated, to give the desired ester.

Procedure F. Under nitrogen atmosphere, methyl 5-bromothiophene-2-carboxylate (1.0 mmol), the appropriate phenylboronic acid (1.3 mmol) and $Pd(PPh_3)Cl_2$ (5% mol) were dissolved in dry 1,4-dioxane (10 mL). Then, a 2 M Na_2CO_3 solution (2.0 mmol) was added, and the resulting mixture was stirred overnight at 90 °C. The mixture was filtered on a celite pad, diluted with H_2O (5 mL) and extracted with EtOAc (3 × 5 mL). The combined organic layers were dried over anhydrous Na_2SO_4, filtered, and concentrated *in vacuo*. The crude product was purified by flash column chromatography to afford the desired product.

Procedure G. A solution of the suitable acetophenone (3.03 mmol) in DCM (0.6 mL) was added dropwise to a solution of NBS (3.64 mmol) and *p*-TsOH (0.3 mmol) in DCM (2.4 mL) at 0 °C. The reaction mixture was then stirred under nitrogen atmosphere at room temperature for 16 h. After the addition of H_2O (3 mL), the organic layer was separated, and the aqueous layer was extracted with DCM (2 × 15 mL). The combined organic layers were washed with saturated aqueous $NaHCO_3$ (10 mL) and brine (10 mL), dried over anhydrous Na_2SO_4, and concentrated under reduced pressure. The purification, performed by flash column chromatography on silica gel, provided the corresponding α-bromoacetophenones.

Procedure H. Hexamine (0.6 mmol) was added to a stirred solution of the suitable α-bromoacetophenone (0.5 mmol) in DCM (1.8 mL), and the reaction mixture was stirred at room temperature for 8 h. After completion, the reaction was filtered, washed with DCM, and dried under reduced pressure to afford an off-white solid that was dissolved in EtOH (1.8 mL) and concentrated HCl (0.1 mL). The reaction mixture was stirred at room temperature for 16 h. The obtained solid was filtered, washed with cold EtOH, and dried to afford the desired hydrochloride salt of the amine.

Procedure I. To a solution of the appropriate hydrochloride salt (1.34 mmol) in EtOAc (5.8 mL) was added TEA (1.61 mmol, 0.22 mL). The mixture was cooled to 5 °C, ethyl oxalyl chloride (1.61 mmol, 0.18 mL) was added dropwise, and the reaction was refluxed for 3 h. After cooling to room temperature and quenching with H_2O, the organic layer was separated, dried over Na_2SO_4, and evaporated under reduced pressure to afford the crude, which was purified by flash column chromatography.

Procedure J. To a solution of the appropriate amide (0.49 mmol) in 1,4-dioxane (2.8 mL), the Lawesson's Reagent (0.54 mmol) was added, and the mixture was refluxed for 2 h. The reaction was subsequently cooled, added to H_2O, and neutralized with a saturated solution of Na_2CO_3. The product was separated from the aqueous phase by extraction with EtOAc (3 × 10 mL). The organic layer was dried over Na_2SO_4, filtered, and

concentrated under reduced pressure to give a dark brown residue, which was purified through flash column chromatography.

Procedure K. Ethyl 2-isocyanoacetate (2.0 mmol) was added to a solution of the appropriate acetophenone (1.0 mmol) and I_2 (1.6 mmol) in DMSO (3 mL). The mixture was stirred at 130 °C for 3 h; after completion, the reaction was quenched with H_2O (50 mL) and extracted with EtOAc (3 × 50 mL). The combined organic layers were washed with brine, dried over anhydrous Na_2SO_4, and concentrated under reduced pressure. The crude was purified by flash column chromatography on silica gel to afford the desired product.

Procedure L. SeO_2 (2.0 mmol) was dissolved in dry 1,4-dioxane (1 mL) and H_2O (2 drops). Then, the suitable acetophenone (1.0 mmol) was added to the solution and the mixture was stirred at reflux for 7 h. After cooling to room temperature, 5 mL of DCM were added, and the reaction mixture was filtered over a pad of celite, washing with DCM. The solution was concentrated *in vacuo*, and the residue was diluted with H_2O (2 mL) and heated at reflux for 10 min. The resulting mixture was cooled in an ice bath affording a precipitate, which was filtered and used in the following reaction without further purification.

Procedure M. Polymerized ethyl 2-oxoacetate in 50% toluene (3.0 mmol) was heated to 60 °C for 15 min before it was added to a solution of NH_4OAc (3.0 mmol) in H_2O (0.65 mL) and CH_3CN (1.3 mL) at 0 °C. Then, a solution of the suitable 2,2-dihydroxyethanone (1.0 mmol) in CH_3CN (1.3 mL) was added dropwise at 0 °C; the resulting mixture was stirred between 0-5 °C for 30 min and then at room temperature for 1.5 h. After the removal of the solvent under reduced pressure, the residue was diluted with H_2O (10 mL) and extracted with EtOAc (3 × 10 mL). The organic layer was washed with brine and dried over anhydrous Na_2SO_4, filtered, and concentrated *in vacuo*. The residue was purified by flash column chromatography and crystallized from EtOAc and hexane to afford the desired product.

Procedure N. NaN_3 (1.5 mmol) was added to a solution of the appropriate boronic acid (1 mmol) and $Cu(OAc)_2$ (0.1 mmol) in dry MeOH (5 mL), under a nitrogen atmosphere. The mixture was stirred at 55 °C for 1.5 h (4 h for **6a**). Then, ethyl propiolate (3 mmol) and (+)-sodium L-ascorbate (0.1 mmol) were added, and the stirring was continued at room temperature overnight (24 h for **6a**). Air was bubbled into the solution for 2 h to oxidize the residual organoboron compound, and then the mixture was diluted with EtOAc (5 mL) and filtered over a pad of celite. The solution was concentrated *in vacuo* to remove the solvent and then washed with H_2O. The organic phase was dried over anhydrous

Na_2SO_4, filtered, and concentrated *in vacuo*; the crude product was purified by flash column chromatography.

5-(3-Cyanophenyl)thiophene-2-carboxylic acid (1a). Methyl 5-(3-cyanophenyl)thiophene-2-carboxylate (**8a**, 1.0 mmol) was dissolved in THF-H_2O 2:1 (6.0 mL), and LiOH (3.0 mmol) was added, keeping the solution in an ice bath. The resulting mixture was stirred at room temperature for 2 h; then, THF was removed under reduced pressure. The aqueous phase was washed with DCM (5 mL), acidified to pH 3-4 with 1 M HCl, and extracted with EtOAc (3 × 5 mL). The combined organic layers were dried over anhydrous Na_2SO_4, filtered, and concentrated *in vacuo*, affording the desired acid. The product was converted to its sodium salt, using equimolar amounts of aqueous $NaHCO_3$, followed by freeze-drying. Yield: 90%.

5-(3-Cyanophenyl)-1H-imidazole-2-carboxylic acid (4a). Ethyl 5-(3-cyanophenyl)-1*H*-imidazole-2-carboxylate (**15a**, 1.0 mmol) was dissolved in THF-H_2O 2:1 (6 mL), and LiOH (3.0 mmol) was added while keeping the solution in an ice bath. The resulting mixture was stirred at room temperature overnight; then, THF was removed under reduced pressure. The aqueous phase was washed with DCM and acidified to pH 3-4 with 3 M HCl until the formation of a white precipitate, which was filtered to give the desired product. Yield: 48%.

1-(3-Cyanophenyl)-1H-1,2,3-triazole-4-carboxylic acid (6a). Ethyl 1-(3-cyanophenyl)-1*H*-1,2,3-triazole-4-carboxylate (**20a**, 1.0 mmol) was dissolved in THF-H_2O 2:1 (6 mL), and LiOH (3.0 mmol) was added while keeping the solution in an ice bath. The resulting mixture was stirred at room temperature overnight; then, THF was removed under reduced pressure. The aqueous phase was washed with DCM, acidified to pH 3-4 with 3 M HCl and extracted with EtOAc (3 × 5 mL). The combined organic layers were dried over anhydrous Na_2SO_4, filtered, and concentrated *in vacuo*, affording the desired acid. Yield: 70%.

Methyl 5-bromo-2-thiophene-carboxylate (7). The compound was obtained according to Procedure E. Starting compound: 5-bromotiophene-2-carboxylic acid. Yield: 97%. Aspect: pale yellow solid. Mp: 60 °C. TLC (cyclohexane-EtOAc 9:1): R_f = 0.60. ^{1}H NMR (300 MHz, $CDCl_3$) δ 7.55 (d, *J* = 4.0 Hz, 1H, ArH), 7.07 (d, *J* = 4.0 Hz, 1H, ArH), 3.87 (s, 3H, CH_3) ppm.

Methyl 5-(3-cyanophenyl)thiophene-2-carboxylate (8a). The compound was obtained according to Procedure F. Starting compound: 3-cyanophenylboronic acid. Purification: cyclohexane-EtOAc 9:1. Yield: 25%. Aspect: white solid. TLC (cyclohexane-EtOAc 8:2): R_f = 0.33. ^{1}H NMR (300 MHz, $CDCl_3$) δ 7.91 (dt, *J* = 1.5, 0.6 Hz, 1H, ArH), 7.85 (dt, *J* = 7.8, 1.5

Hz, 1H, ArH), 7.79 (d, *J* = 3.9 Hz, 1H, ArH), 7.64 (dt, *J* = 7.8, 1.5 Hz, 1H, ArH), 7.54 (td, *J* = 7.8, 0.6 Hz, 1H, ArH), 7.34 (d, *J* = 3.9 Hz, 1H, ArH), 3.92 (s, 3H, CH_3) ppm.

Methyl 5-(4-nitrophenyl)thiophene-2-carboxylate (8b). The compound was obtained according to Procedure F. Starting compound: 4-nitrophenylboronic acid. Purification: cyclohexane-EtOAc 95:5. Yield: 26%. Aspect: yellow solid. Mp: 192 °C. TLC (cyclohexane-EtOAc 8:2): R_f = 0.50. ^{1}H NMR (300 MHz, $CDCl_3$) δ 8.28 (d, *J* = 8.9 Hz, 2H, ArH), 7.81 – 7.77 (m, 3H, ArH), 7.44 (d, *J* = 3.9 Hz, 1H, ArH), 3.93 (s, 3H, CH_3) ppm.

3-(2-Bromoacetyl)benzonitrile (9a). The compound was obtained according to Procedure G. Starting compound: 3-acetylbenzonitrile. Purification: cyclohexane-EtOAc 8:2. Yield: 20%. Aspect: white solid. Mp: 105 °C. TLC (cyclohexane-EtOAc 8:2): R_f = 0.30. ^{1}H NMR (300 MHz, $CDCl_3$) δ 8.27 (s, 1H, ArH), 8.22 (d, *J* = 7.8 Hz, 1H, ArH), 7.89 (d, *J* = 7.8 Hz, 1H, ArH), 7.66 (t, *J* = 7.8 Hz, 1H, ArH), 4.43 (s, 2H, CH_2) ppm.

2-Bromo-1-(4-nitrophenyl)ethenone (9b). The compound was obtained according to Procedure G. Starting compound: 1-(4-nitrophenyl)ethanone. Purification: cyclohexane-EtOAc 8:2. Yield: 45%. Aspect: white solid. Mp: 105 °C. TLC (cyclohexane-EtOAc 8:2): R_f = 0.38. ^{1}H NMR (300 MHz, $CDCl_3$) δ 8.36 (dd, *J* = 9.0, 2.1 Hz, 2H, ArH), 8.17 (dd, *J* = 9.0, 2.1 Hz, 2H, ArH), 4.47 (s, 2H, CH_2) ppm.

2-(3-Cyanophenyl)-2-oxoethanaminium chloride (10a). The compound was obtained according to Procedure H. Starting compound: 3-(2-bromoacetyl)benzonitrile. Yield: 65%. Aspect: white solid. Mp: 225 °C (dec.). TLC (DCM-MeOH 9:1): R_f = 0.24. ^{1}H NMR (300 MHz, DMSO-d_6) δ 8.52 (t, *J* = 1.4 Hz, 1H, ArH), 8.42 (br s exch. D_2O, 3H, NH_3), 8.30 (dt, *J* = 7.8, 1.4 Hz, 1H, ArH), 8.21 (dt, *J* = 7.8, 1.4 Hz, 1H, ArH), 7.82 (t, *J* = 7.8 Hz, 1H, ArH), 4.65 (s, 2H, CH_2) ppm.

2-(4-Nitrophenyl)-2-oxoethanaminium chloride (10b). The compound was obtained according to Procedure H. Starting compound: 2-bromo-1-(4-nitrophenyl)ethenone. Yield: 54%. Aspect: white solid. Mp: 229 °C (dec.). TLC (DCM-MeOH 9:1): R_f = 0.17. ^{1}H NMR (300 MHz, DMSO-d_6) δ 8.61 (br s exch. D_2O, 3H, NH_3), 8.38 (d, *J* = 8.8 Hz, 2H, ArH), 8.26 (d, *J* = 8.8 Hz, 2H, ArH), 4.67 (s, 2H, CH_2) ppm.

Ethyl 2-((2-(3-cyanophenyl)-2-oxoethyl)amino)-2-oxoacetate (11a). The compound was obtained according to Procedure I. Starting compound: 2-(3-cyanophenyl)-2-oxoethanaminium chloride. Purification: cyclohexane-EtOAc 5:5. Yield: 61%. Aspect: pale yellow solid. TLC (cyclohexane-EtOAc 6:4): R_f = 0.12. ^{1}H NMR (300 MHz, $CDCl_3$) δ 8.29 (td, *J* = 1.7, 0.6 Hz, 1H, ArH), 8.21 (dt, *J* = 7.8, 1.7, 1H, ArH), 8.00 (br s exch. D_2O, 1H,

NH), 7.93 (dt, *J* = 7.8, 1.7, 1H, ArH), 7.69 (td, *J* = 7.8, 0.6 Hz, 1H, ArH), 4.85 (d, *J* = 4.9 Hz, 2H, CH_2), 4.41 (q, *J* = 7.1 Hz, 2H, O*CH_2*), 1.42 (t, *J* = 7.1 Hz, 3H, CH_3) ppm.

Ethyl 2-((2-(4-nitrophenyl)-2-oxoethyl)amino)-2-oxoacetate (11b). The compound was obtained according to Procedure I. Starting compound: 2-(4-nitrophenyl)-2-oxoethanaminium chloride. Purification: cyclohexane-EtOAc 5:5. Yield: 42%. Aspect: yellow solid. Mp: 152 °C. TLC (cyclohexane-EtOAc 6:4): R_f = 0.15. ^{1}H NMR (300 MHz, $CDCl_3$) δ 8.39 (d, *J* = 8.8 Hz, 2H, ArH), 8.18 (d, *J* = 8.8 Hz, 2H, ArH), 8.01 (br s exch. D_2O, 1H, NH), 4.89 (d, *J* = 4.8 Hz, 2H, CH_2), 4.42 (q, *J* = 7.1 Hz, 2H, O*CH_2*), 1.43 (t, *J* = 7.1 Hz, 3H, CH_3) ppm.

Ethyl 5-(3-cyanophenyl)thiazole-2-carboxylate (12a). The compound was obtained according to Procedure J. Starting compound: ethyl 2-((2-(3-cyanophenyl)-2-oxoethyl)amino)-2-oxoacetate. Purification: cyclohexane-EtOAc 7:3. Yield: 60%. Aspect: white solid. TLC (cyclohexane-EtOAc 6:4): R_f = 0.43. ^{1}H NMR (300 MHz, $CDCl_3$) δ 8.19 (s, 1H, ArH), 7.89 (td, *J* = 1.7, 0.6 Hz, 1H, ArH), 7.84 (dt, *J* = 7.8, 1.7, 1H, ArH), 7.69 (dt, *J* = 7.8, 1.7 Hz, 1H, ArH), 7.58 (td, *J* = 7.8, 0.6 Hz, 1H, ArH), 4.50 (q, *J* = 7.1 Hz, 2H, CH_2), 1.46 (t, *J* = 7.1 Hz, 3H, CH_3) ppm.

Ethyl 5-(4-nitrophenyl)thiazole-2-carboxylate (12b). The compound was obtained according to Procedure J. Starting compound: ethyl 2-((2-(4-nitrophenyl)-2-oxoethyl)amino)-2-oxoacetate. Purification: cyclohexane-EtOAc 7:3. Yield: 88%. Aspect: pale yellow solid. Mp: 173 °C. TLC (cyclohexane-EtOAc 6:4): R_f = 0.45. ^{1}H NMR (300 MHz, $CDCl_3$) δ 8.33 – 8.28 (m, 3H, ArH), 7.80 (d, *J* = 9.1 Hz, 2H, ArH), 4.51 (q, *J* = 7.1 Hz, 2H, CH_2), 1.47 (t, *J* = 7.1 Hz, 3H, CH_3) ppm.

Ethyl 5-(3-cyanophenyl)oxazole-2-carboxylate (13a). The compound was obtained according to Procedure K. Starting compound: 3-acetylbenzonitrile. Purification: cyclohexane-EtOAc 8:2. Yield: 6%. Aspect: beige solid. Mp: 154 °C. TLC (cyclohexane-EtOAc 6:4): R_f = 0.46. ^{1}H NMR (300 MHz, $CDCl_3$) δ 8.06 (t, *J* = 1.6 Hz, 1H, ArH), 7.99 (dt, *J* = 7.8, 1.6 Hz, 1H, ArH), 7.70 (dt, *J* = 7.8, 1.6 Hz, 1H, ArH), 7.64 – 7.58 (m, 2H, ArH), 4.52 (q, *J* = 7.1 Hz, 2H, CH_2), 1.48 (t, *J* = 7.1 Hz, 3H, CH_3) ppm.

Ethyl 5-(4-nitrophenyl)oxazole-2-carboxylate (13b). The compound was obtained according to Procedure K. Starting compound: 1-(4-nitrophenyl)ethanone. Purification: cyclohexane-EtOAc 8:2. Yield: 7%. Aspect: yellow solid. Mp: 182 °C. TLC (cyclohexane-EtOAc 8:2): R_f = 0.15. ^{1}H NMR (300 MHz, $CDCl_3$) δ 8.35 (d, *J* = 9.0 Hz, 2H, ArH), 7.94 (d, *J* = 9.0 Hz, 2H, ArH), 7.72 (s, 1H, ArH), 4.53 (q, *J* = 7.1 Hz, 2H, CH_2), 1.48 (t, *J* = 7.1 Hz, 3H, CH_3) ppm.

3-(2,2-Dihydroxyacetyl)benzonitrile (14a). The compound was obtained according to Procedure L. Starting compound: 3-acetylbenzonitrile. Not isolated.

2,2-Dihydroxy-1-(4-nitrophenyl)ethenone (14b). The compound was obtained according to Procedure L. Starting compound: 1-(4-nitrophenyl)ethanone. Not isolated.

Ethyl 5-(3-cyanophenyl)-1H-imidazole-2-carboxylate (15a). The compound was obtained according to Procedure M. Starting compound: 3-(2,2-dihydroxyacetyl)benzonitrile. Purification: cyclohexane-EtOAc 7:3. Yield: 40%. Aspect: white solid. Mp: 176 °C. TLC (cyclohexane-EtOAc 6:4): R_f = 0.42. ^{1}H NMR (300 MHz, $CDCl_3$) δ 10.72 (br s exch. D_2O, 1H, NH), 8.14 (s, 1H, ArH), 8.07 (d, *J* = 7.8 Hz, 1H, ArH), 7.58 – 7.46 (m, 3H, ArH), 4.49 (q, *J* = 7.1 Hz, 2H, CH_2), 1.44 (t, *J* = 7.1 Hz, 3H, CH_3) ppm.

Ethyl 5-(4-nitrophenyl)-1H-imidazole-2-carboxylate (15b). The compound was obtained according to Procedure M. Starting compound: 2,2-dihydroxy-1-(4-nitrophenyl)ethenone. Purification: cyclohexane-EtOAc 6:4. Yield: 31%. Aspect: yellow solid. TLC (cyclohexane-EtOAc 6:4): R_f = 0.39. ^{1}H NMR (300 MHz, $CDCl_3$) δ 8.27 (d, *J* = 8.7 Hz, 2H, ArH), 8.00 (d, *J* = 8.7 Hz, 2H, ArH), 7.63 (s, 1H, ArH), 4.52 (q, *J* = 7.1 Hz, 2H, CH_2), 1.47 (t, *J* = 7.1 Hz, 3H, CH_3) ppm.

Methyl 3-cyanobenzoate (16a). The compound was obtained according to Procedure E. The reaction was conducted in inert atmosphere to reduce the risk of hydrolyzation of the nitrile group. Starting compound: 3-cyanobenzoic acid. Yield: 76%. Aspect: white solid. Mp: 61 °C. TLC (cyclohexane-EtOAc 7:3): R_f = 0.64. ^{1}H NMR (300 MHz, $CDCl_3$) δ 8.33 (s, 1H, ArH), 8.27 (d, *J* = 7.9 Hz, 1H, ArH), 7.84 (d, *J* = 7.9 Hz, 1H, ArH), 7.59 (t, *J* = 7.9 Hz, 1H, ArH), 3.96 (s, 3H, CH_3) ppm.

Ethyl 4-nitrobenzoate (16b). The compound was obtained according to Procedure E. Starting compound: 4-nitrobenzoic acid. Yield: 81%. Aspect: yellow solid. Mp: 57 °C. TLC (cyclohexane-EtOAc 8:2): R_f = 0.68. ^{1}H NMR (300 MHz, $CDCl_3$) δ 8.28 (d, *J* = 9.1 Hz, 2H, ArH), 8.21 (d, *J* = 9.1 Hz, 2H, ArH), 4.44 (q, *J* = 7.1 Hz, 2H, CH_2), 1.43 (t, *J* = 7.1 Hz, 3H, CH_3) ppm.

3-Cyanobenzohydrazide (17a). $NH_2NH_2 \cdot H_2O$ (2.5 mmol) was added to a solution of methyl 3-cyanobenzoate (**16a**, 1.0 mmol) in MeOH (2 mL) at 0 °C. Then, the reaction mixture was stirred at room temperature overnight. The formed precipitate was filtered to give the pure product. Yield: 65%. Aspect: white solid. Mp: 170 °C. TLC (cyclohexane-EtOAc 7:3): R_f = 0.10. ^{1}H NMR (300 MHz, DMSO-*d*$_6$) δ 9.98 (br s exch. D_2O, 1H, NH), 8.21 (s, 1H, ArH), 8.13 (d, *J* = 7.8 Hz, 1H, ArH), 8.00 (d, *J* = 7.8 Hz, 1H, ArH), 7.69 (t, *J* = 7.8 Hz, 1H, ArH), 4.57 (br s exch. D_2O, 2H, NH_2) ppm.

4-Nitrobenzohydrazide (17b). $NH_2NH_2{\cdot}H_2O$ (2.0 mmol) was added to a solution of ethyl 4-nitrobenzoate (**16b**, 1.0 mmol) in EtOH (1 mL), and the resulting mixture was stirred at reflux overnight. After cooling to room temperature, EtOH was removed under reduced pressure and the crude product was washed with hexane, affording the desired product. Yield: 84%. Aspect: yellow solid. Mp: 217 °C. TLC (cyclohexane-EtOAc 6:4): R_f = 0.25. ^1H NMR (300 MHz, DMSO-d_6) δ 10.10 (br s exch. D_2O, 1H, NH), 8.28 (d, J = 7.0 Hz, 2H, ArH), 8.03 (d, J = 7.0 Hz, 2H, ArH), 4.62 (br s exch. D_2O, 2H, NH_2) ppm.

Ethyl 2-(2-(3-cyanobenzoyl)hydrazinyl)-2-oxoacetate (18a). Triethylamine (6.0 mmol) was added to a solution of 3-cyanobenzohydrazide (**17a**, 1.0 mmol) in DCM (3.2 mL) at 0 °C. The mixture was stirred for 5 min at 0 °C before adding ethyl chlorooxoacetate (2.2 mmol) dropwise at 0 °C. The resulting mixture was stirred at room temperature for 2 h. Then, it was diluted with H_2O (10 mL) and extracted with DCM (3 × 10 mL). The combined organic layers were dried over anhydrous Na_2SO_4, filtered, and concentrated under reduced pressure. The crude product (**18a**) was used in the following step without any further purification. Not isolated.

Ethyl 5-(3-cyanophenyl)-1,3,4-oxadiazole-2-carboxylate (19a). Triethylamine (3.0 mmol) was added to a stirred solution of ethyl 2-(2-(3-cyanobenzoyl)hydrazinyl)-2-oxoacetate (**18a**, 1.0 mmol) in DCM (3.2 mL) at 0 °C. The reaction mixture was stirred at 0 °C for 5 min before the addition of *p*-toluenesulfonyl chloride (2.2 mmol) at 0 °C. The resulting mixture was stirred at room temperature for 2 h; then, it was diluted with H_2O (13 mL) and extracted with DCM (3 x 10 mL). The combined organic layers were dried over anhydrous Na_2SO_4, filtered, and concentrated under reduced pressure. The crude product was purified by flash column chromatography (cyclohexane-EtOAc 7:3) to afford the desired product (**19a**). Yield: 40%. Aspect: pale yellow solid. TLC (cyclohexane-EtOAc 7:3): R_f = 0.26. ^1H NMR (300 MHz, $CDCl_3$) δ 8.47 (s, 1H, ArH), 8.43 (d, J = 7.9 Hz, 1H, ArH), 7.90 (d, J = 7.9 Hz, 1H, ArH), 7.72 (t, J = 7.9 Hz, 1H, ArH), 4.58 (q, J = 7.1 Hz, 2H, CH_2), 1.50 (t, J = 7.1 Hz, 3H, CH_3) ppm.

Ethyl 5-(4-nitrophenyl)-1,3,4-oxadiazole-2-carboxylate (19b). 86% polyphosphoric acid (1500 mg) and ethyl 2-nitroacetate (2.0 mmol) were heated to 120-130 °C while stirring. 4-Nitrobenzohydrazide (**17b**, 1.0 mmol) was added slowly in small portions over a period of 1 h; then, the mixture was heated for an additional 30 min. After cooling to room temperature, the mixture was diluted with H_2O (6 mL), neutralized with 25% aqueous NH_3 and extracted with EtOAc (3 × 6 mL). The combined organic layers were dried over anhydrous Na_2SO_4, filtered, and concentrated *in vacuo.* The crude product was purified by flash column chromatography (cyclohexane-EtOAc 9:1) to afford the desired product.

Yield: 10%. Aspect: white solid. TLC (cyclohexane-EtOAc 8:2): R_f = 0.66. ^{1}H NMR (300 MHz, $CDCl_3$) δ 8.29 (d, *J* = 9.1 Hz, 2H, ArH), 8.22 (d, *J* = 9.1 Hz, 2H, ArH), 4.44 (q, *J* = 7.1 Hz, 2H, CH_2), 1.43 (t, *J* = 7.1 Hz, 3H, CH_3) ppm.

Ethyl 1-(3-cyanophenyl)-1H-1,2,3-triazole-4-carboxylate (20a). The compound was obtained according to Procedure N. Starting compound: (3-cyanophenyl)boronic acid. Purification: cyclohexane–EtOAc 7:3. Yield: 20%. Aspect: off-white solid. TLC (cyclohexane–EtOAc 8:2): R_f = 0.24. ^{1}H NMR (300 MHz, $CDCl_3$) δ 8.55 (s, 1H, ArH), 8.11 (s, 1H, ArH), 8.05 (d, *J* = 7.8 Hz, 1H, ArH), 7.80 (d, *J* = 7.8 Hz, 1H, ArH), 7.72 (t, *J* = 7.8 Hz, 1H, ArH), 4.49 (q, *J* = 7.1 Hz, 2H, CH_2), 1.45 (t, *J* = 7.1 Hz, 3H, CH_3) ppm.

Ethyl 1-(4-nitrophenyl)-1H-1,2,3-triazole-4-carboxylate (20b). The compound was obtained according to Procedure N. Starting compound: (4-nitrophenyl)boronic acid. Purification: cyclohexane–EtOAc 7:3. Yield: 30%. Aspect: white solid. Mp: 196 °C. TLC (cyclohexane–EtOAc 7:3): R_f = 0.37. ^{1}H NMR (300 MHz, $CDCl_3$) δ 8.62 (s, 1H, ArH), 8.46 (d, *J* = 9.1 Hz, 2H, ArH), 8.02 (d, *J* = 9.1 Hz, 2H, ArH), 4.49 (q, *J* = 7.1 Hz, 2H, CH_2), 1.45 (t, *J* = 7.1 Hz, 3H, CH_3) ppm.

Methyl 5-(3-cyano-5-(trifluoromethyl)phenyl)furan-2-carboxylate (IV-E). The compound was obtained according to a previously published procedure (see ref. 13 in the main text). Yield: 30%. Aspect: white solid. Mp: 135 °C. TLC (cyclohexane–EtOAc 8:2): R_f = 0.44. ^{1}H NMR (300 MHz, $CDCl_3$) δ 8.22-8.20 (m, 2H, ArH), 7.86 (s, 1H, ArH), 7.29 (d, *J* = 3.7 Hz, 1H, ArH), 6.94 (d, *J* = 3.7 Hz, 1H, ArH), 3.95 (s, 3H, CH_3) ppm.

Methyl 5-(3,5-bis(trifluoromethyl)phenyl)furan-2-carboxylate (V-E). The compound was obtained according to Procedure F. Yield: 62%. Aspect: white solid. Mp: 139 °C. TLC (cyclohexane–EtOAc 8:2): R_f = 0.54. ^{1}H NMR (300 MHz, $CDCl_3$) δ 8.20-8.15 (m, 2H, ArH), 7.84 (s, 1H, ArH), 7.29 (d, *J* = 3.7 Hz, 1H, ArH), 6.94 (d, *J* = 3.7 Hz, 1H, ArH), 3.95 (s, 3H, CH_3) ppm.

Methyl 5-(3-cyano-5-fluorophenyl)furan-2-carboxylate (VI-E). The compound was obtained according to a previously published procedure (see ref. 13 in the main text). Yield: 25%. Aspect: white solid. Mp: 149 °C. TLC (cyclohexane–EtOAc 8:2): R_f = 0.35. ^{1}H NMR (300 MHz, $CDCl_3$) δ 7.84 (t, *J* = 1.5 Hz, 1H, ArH), 7.71 (ddd, *J* = 9.2, 2.5, 1.5 Hz, 1H, ArH), 7.32 (ddd, *J* = 7.7, 2.5, 1.5 Hz, 1H, ArH), 7.27 (d, *J* = 3.7 Hz, 1H, ArH), 6.86 (d, *J* = 3.7 Hz, 1H, ArH), 3.94 (s, 3H, CH_3) ppm.

Methyl 5-(3-cyano-5-methoxyphenyl)furan-2-carboxylate (VII-E). The compound was obtained according to a previously published procedure (see ref. 13 in the main text). Yield: 35%. Aspect: white solid. Mp: 157 °C. TLC (cyclohexane–EtOAc 8:2): R_f = 0.36. ^{1}H

NMR (300 MHz, $CDCl_3$) δ 7.64 (t, *J* = 1.4 Hz, 1H, ArH), 7.51 (dd, *J* = 2.7, 1.4 Hz, 1H, ArH), 7.27 (d hidden by solvent peak, 1H, ArH), 7.12 (dd, *J* = 2.7, 1.4 Hz, 1H, ArH), 6.82 (d, *J* = 3.6 Hz, 1H, ArH), 3.94 (s, 3H, CH_3), 3.91 (s, 3H, CH_3) ppm.

Methyl 5-(3-cyano-5-methylphenyl)furan-2-carboxylate (VIII-E). The compound was obtained according to a previously published procedure (see ref. 13 in the main text). Yield: 65%. Aspect: white solid. Mp: 156 °C. TLC (cyclohexane–EtOAc 8:2): R_f = 0.33. ^1H NMR (300 MHz, $CDCl_3$) δ 7.85 (s, 1H, ArH), 7.82 (s, 1H, ArH), 7.43 (s, 1H, ArH), 7.26 (d partially hidden by solvent peak, *J* = 3.7 Hz, 1H, ArH), 6.80 (d, *J* = 3.7 Hz, 1H, ArH), 3.94 (s, 3H, OCH_3), 2.45 (s, 3H, CH_3) ppm.

Methyl 5-(3-cyano-5-hydroxyphenyl)furan-2-carboxylate (IX-E). The compound was obtained according to a previously published procedure (see ref. 13 in the main text). Yield: 98%. Aspect: beige solid. Mp: 227 °C. TLC (cyclohexane–EtOAc 7:3): R_f = 0.12. ^1H NMR (300 MHz, DMSO-d_6) δ 10.55 (br s exch. D_2O, 1H, OH), 7.73 (t, *J* = 1.5 Hz, 1H, ArH), 7.49 (dd, *J* = 2.4, 1.5 Hz, 1H, ArH), 7.43 (d, *J* = 3.7 Hz, 1H, ArH), 7.30 (d, *J* = 3.7 Hz, 1H, ArH), 7.15 (dd, *J* = 2.4, 1.5 Hz, 1H, ArH), 3.83 (s, 3H, CH_3) ppm.

Chemistry – Experimental data

1. **5-(3-Cyanophenyl)thiophene-2-carboxylic acid (as sodium salt) (1a)**

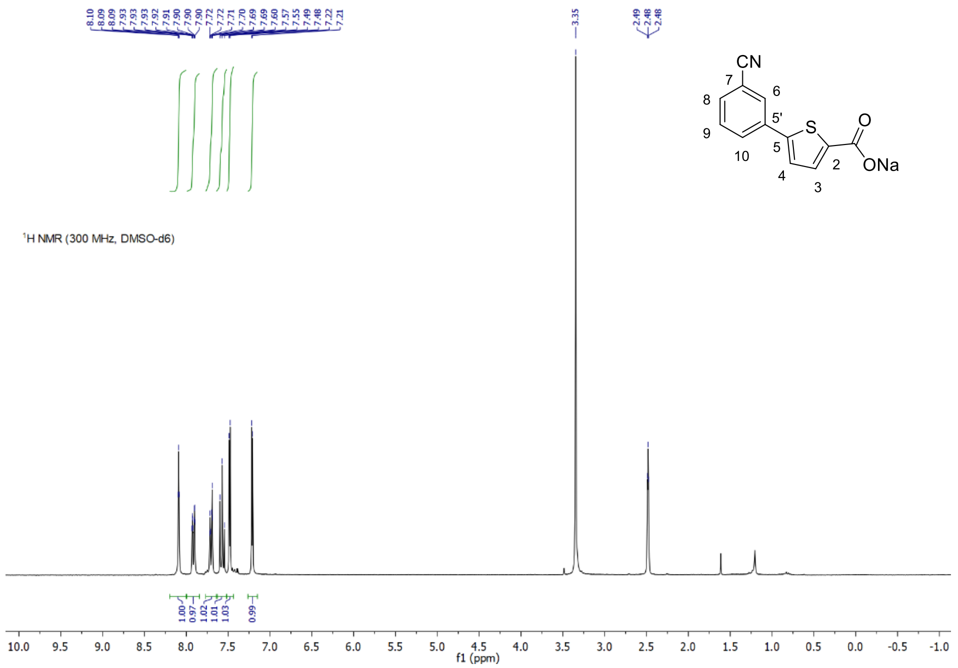

Figure S1. ^{1}H NMR spectrum of **1a**.

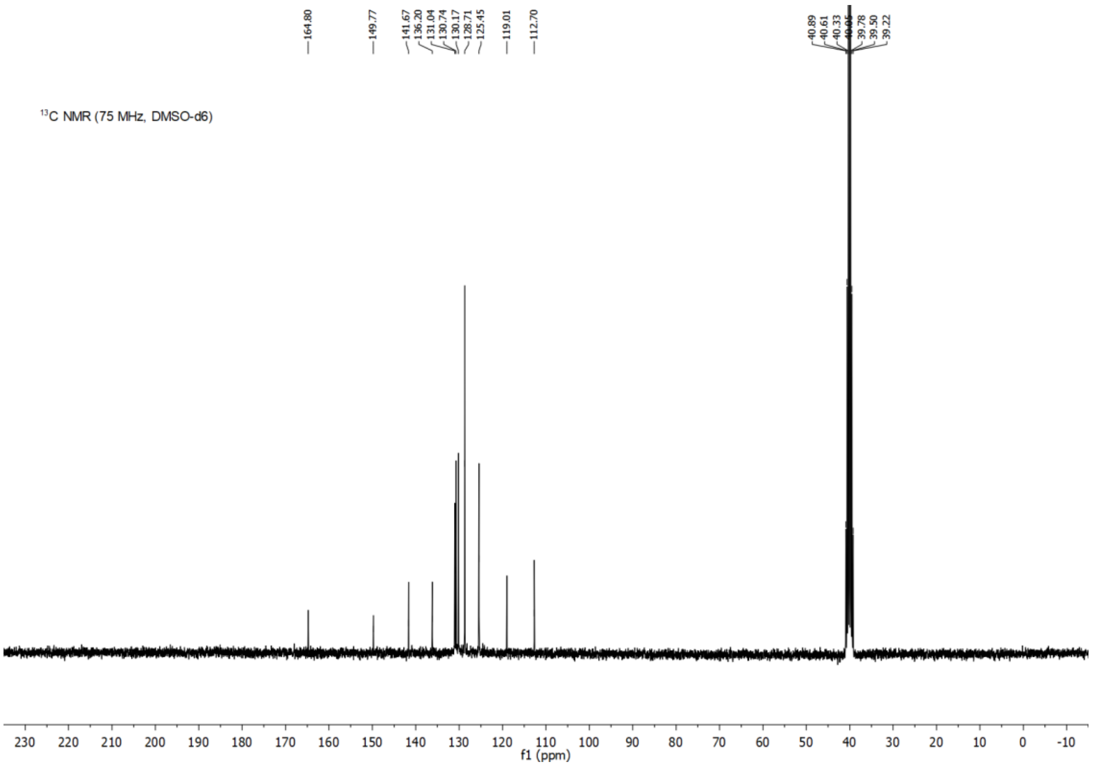

Figure S2. ^{13}C NMR spectrum of **1a**.

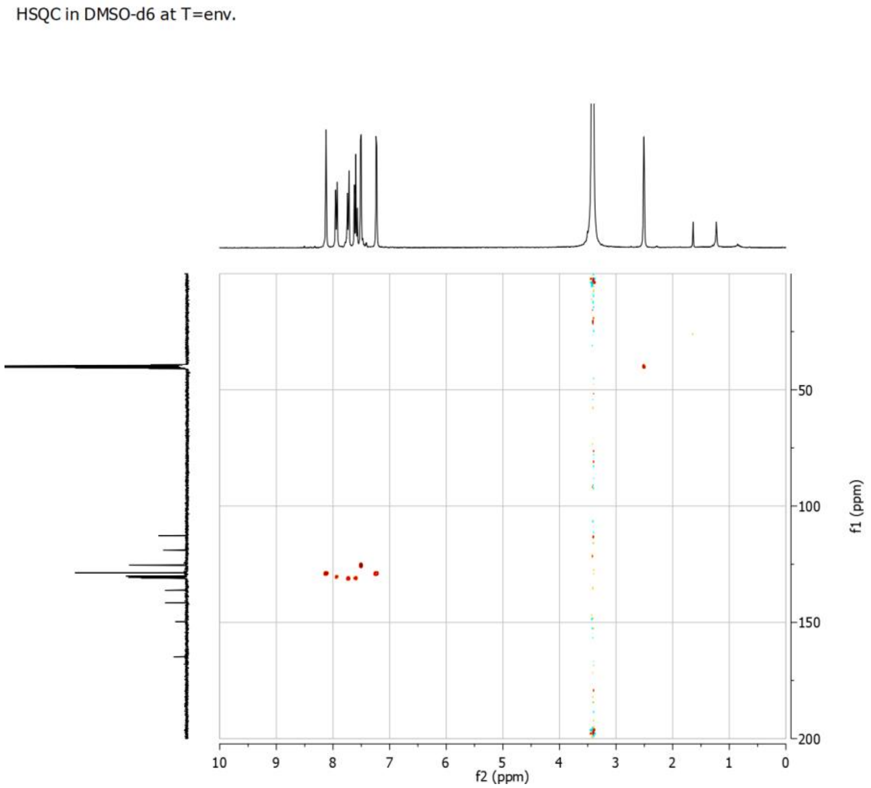

Parameter	Value
1 Data File Name	C:/ Users/ Donatella/ Desktop/ NMR/ 300MHz - Americo/ PIN_GW3/ 2/ ser
2 Title	PIN_GW3.2.ser
3 Comment	GW3 HSQC in DMSO a T=amb.
4 Origin	Bruker BioSpin GmbH
5 Owner	nmr
6 Site	
7 Instrument	spect
8 Author	
9 Solvent	DMSO
10 Temperature	300.0
11 Pulse Sequence	hsqcetgpsi2
12 Experiment	HSQC
13 Probe	5 mm QNP 1H/ 13C/ 31P/ 19F Z-grad Z8352/ 0036
14 Number of Scans	32
15 Receiver Gain	46300.0
16 Relaxation Delay	2.0000
17 Pulse Width	11.7500
18 Presaturation Frequency	
19 Acquisition Time	0.3408
20 Acquisition Date	2021-01-28T10:39:48
21 Modification Date	2021-01-29T10:43:00
22 Class	
23 Spectrometer Frequency	(300.13, 75.48)
24 Spectral Width	(3004.8, 15095.1)
25 Lowest Frequency	(-1.8, -0.8)
26 Nucleus	(1H, 13C)
27 Acquired Size	(1024, 256)
28 Spectral Size	(2048, 2048)
29 Digital Resolution	(1.47, 7.37)

Figure S3. HSQC spectrum of **1a**.

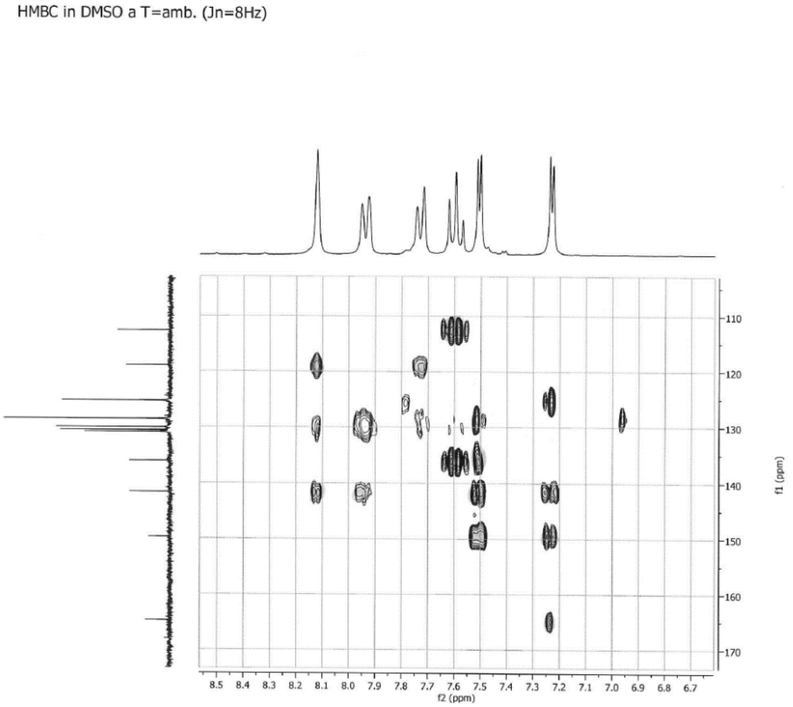

Parameter	Value
1 Data File Name	C:/ Users/ Donatella/ Desktop/ NMR/ 300MHz - Americo/ PIN_GW3/ 4/ ser
2 Title	PIN_GW3.4.ser
3 Comment	GW3 HMBC in DMSO a T=amb. (Jn=8Hz)
4 Origin	Bruker BioSpin GmbH
5 Owner	nmr
6 Site	
7 Instrument	spect
8 Author	
9 Solvent	DMSO
10 Temperature	300.0
11 Pulse Sequence	hmbcgplpndqf
12 Experiment	HMBC
13 Probe	5 mm QNP 1H/ 13C/ 31P/ 19F Z-grad Z8352/ 0036
14 Number of Scans	32
15 Receiver Gain	46300.0
16 Relaxation Delay	2.0000
17 Pulse Width	11.7500
18 Presaturation Frequency	
19 Acquisition Time	0.3408
20 Acquisition Date	2021-01-29T12:25:54
21 Modification Date	2021-01-29T15:12:00
22 Class	
23 Spectrometer Frequency	(300.13, 75.48)
24 Spectral Width	(3004.8, 15095.1)
25 Lowest Frequency	(-1.8, -0.8)
26 Nucleus	(1H, 13C)
27 Acquired Size	(1024, 85)
28 Spectral Size	(2048, 2048)
29 Digital Resolution	(1.47, 7.37)

Figure S4. HMBC spectrum of **1a**.

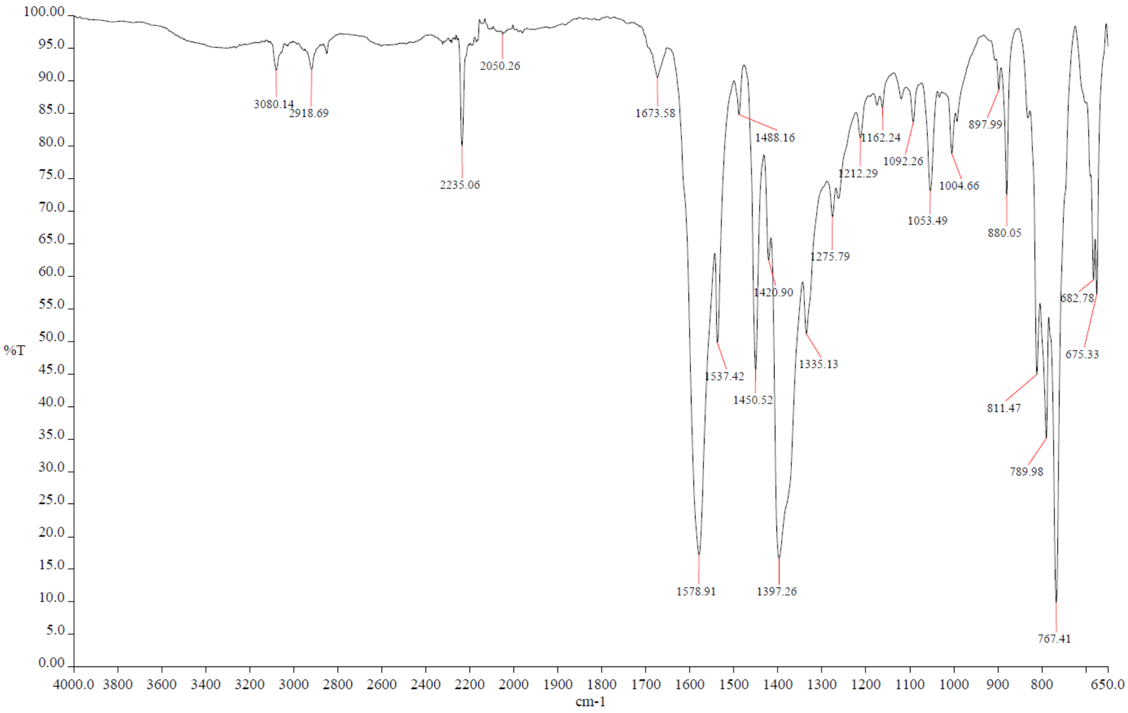

Figure S5. FT-IR spectrum of **1a**.

2. 5-(4-Nitrophenyl)thiophene-2-carboxylic acid (as sodium salt) (1b)

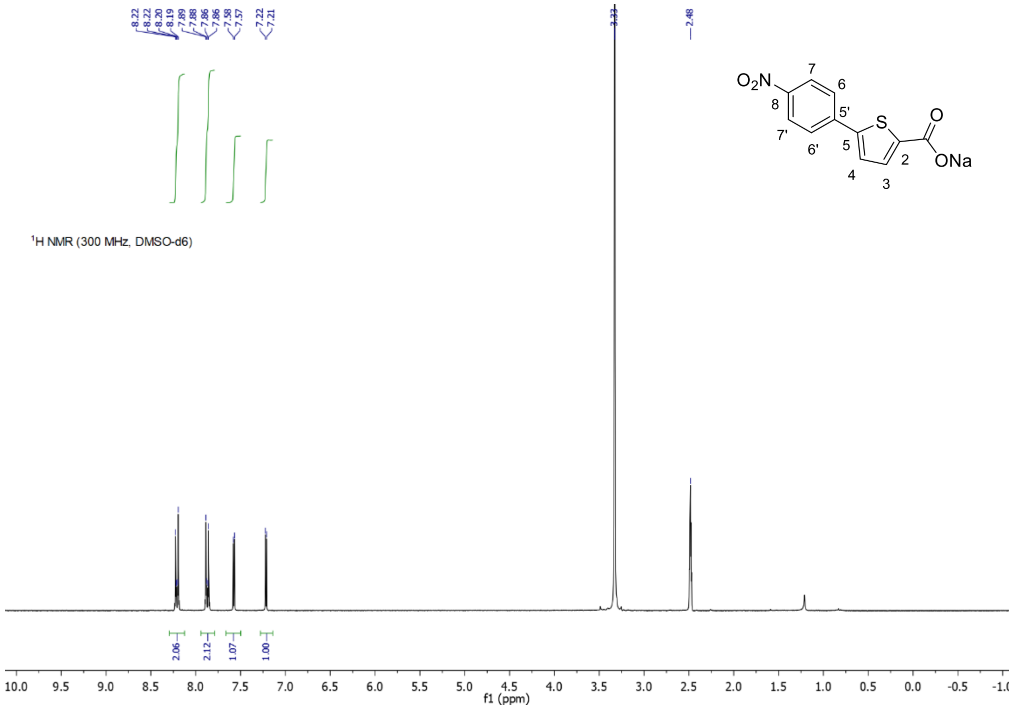

Figure S6. ^{1}H NMR spectrum of **1b**.

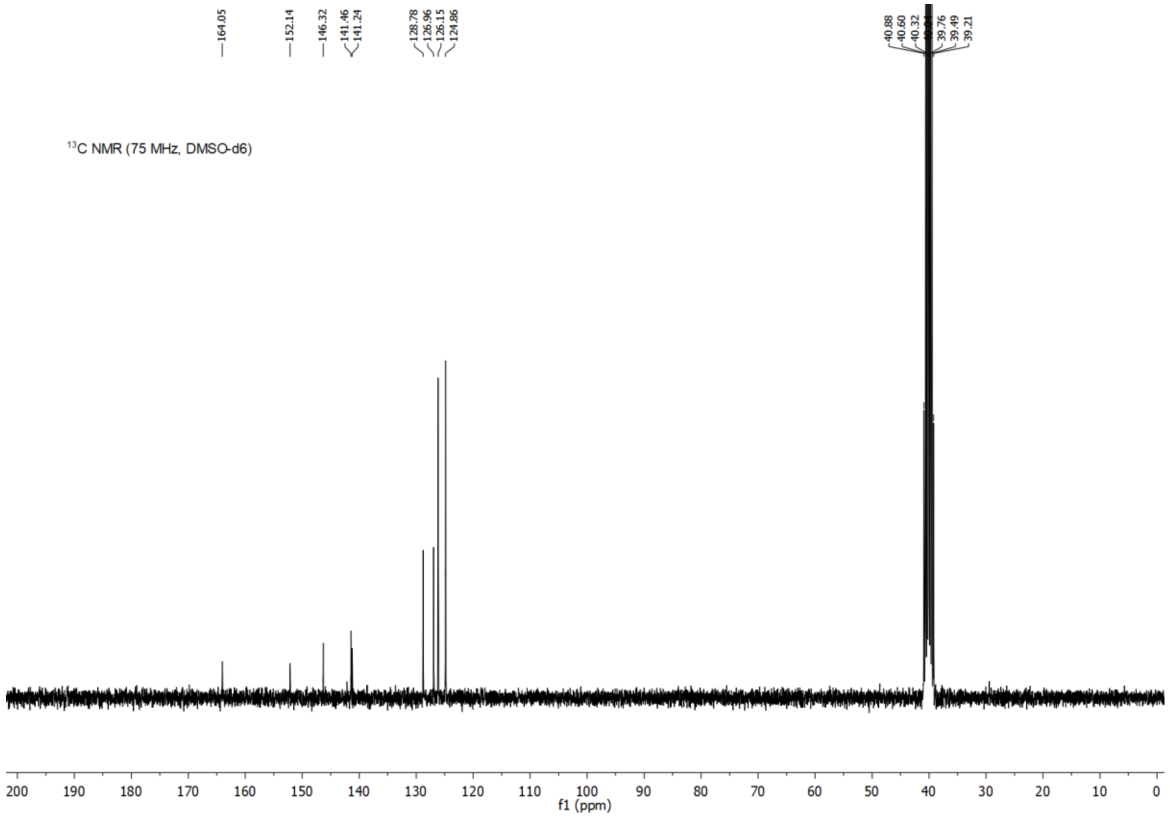

Figure S7. ^{13}C NMR spectrum of **1b**.

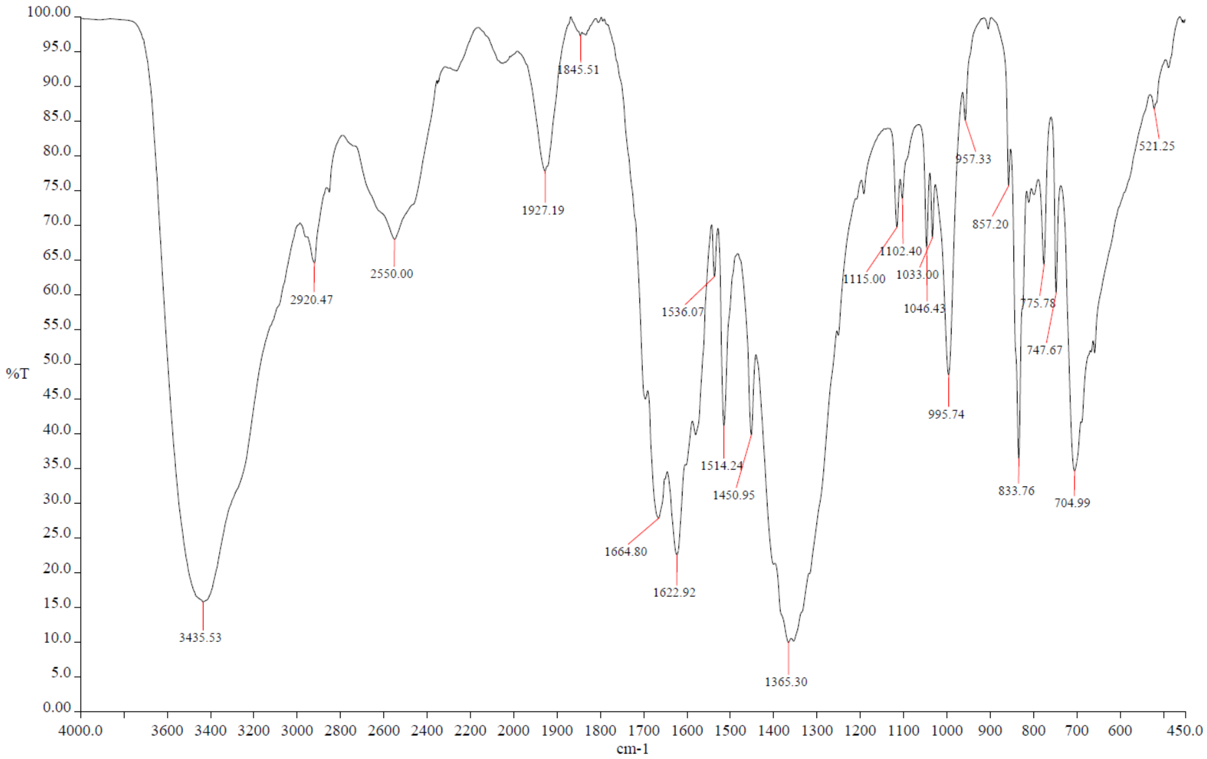

Figure S8. FT-IR spectrum of **1b**.

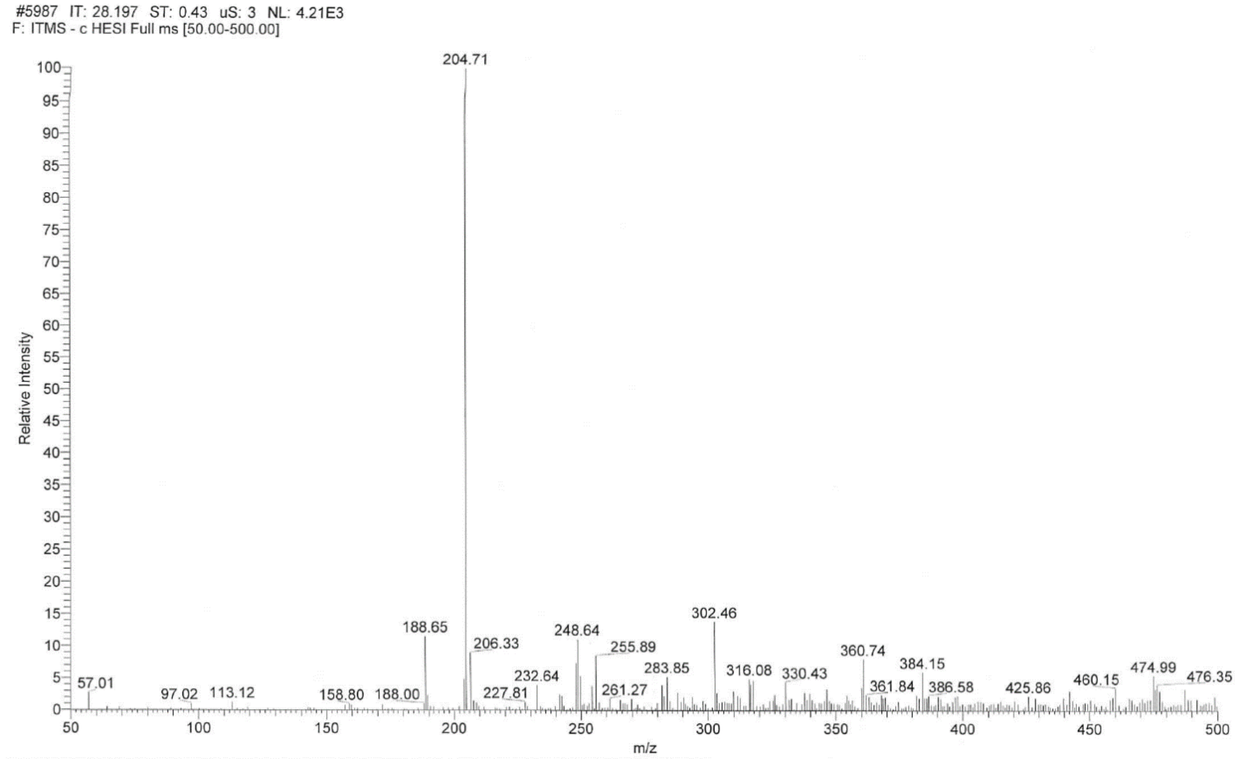

Figure S9. ESI-MS spectrum of **1b**.

3. Sodium 5-(3-cyanophenyl)thiazole-2-carboxylate (2a)

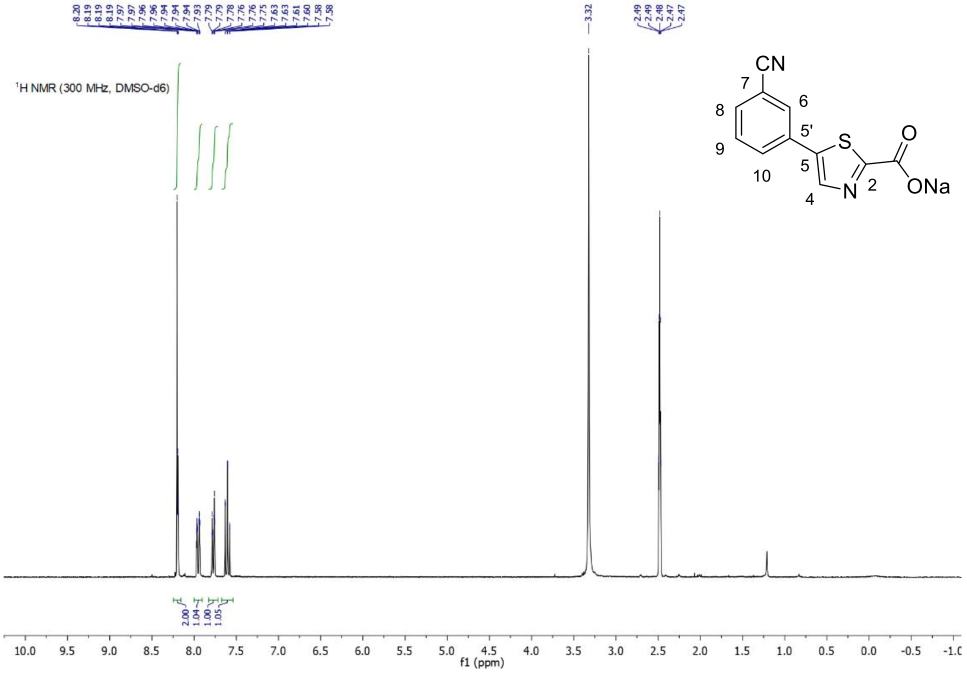

Figure S10. ^{1}H NMR spectrum of **2a**.

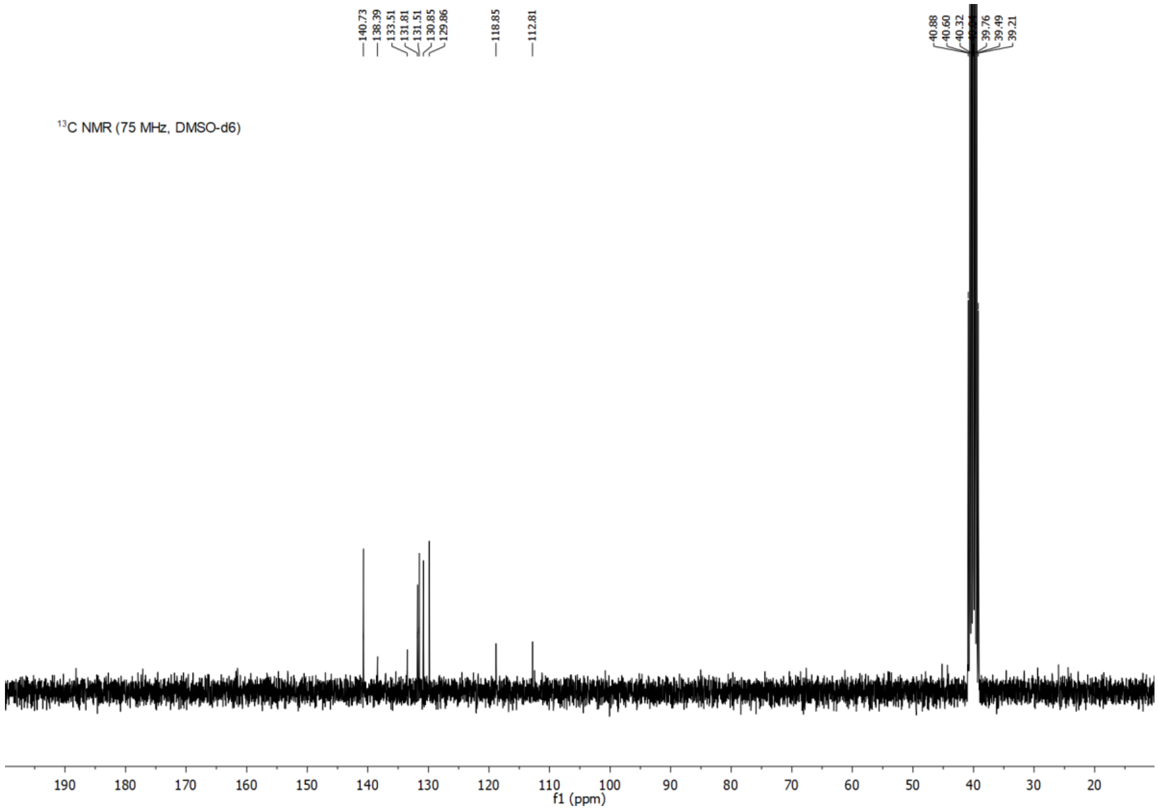

Figure S11. ^{13}C NMR spectrum of **2a**.

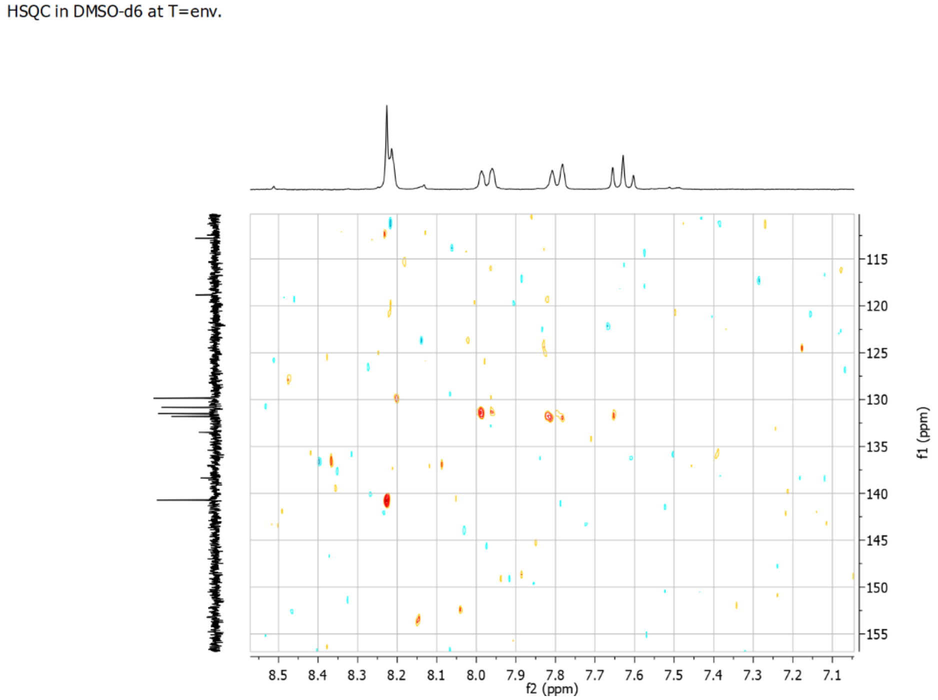

Figure S12. HSQC spectrum of **2a**.

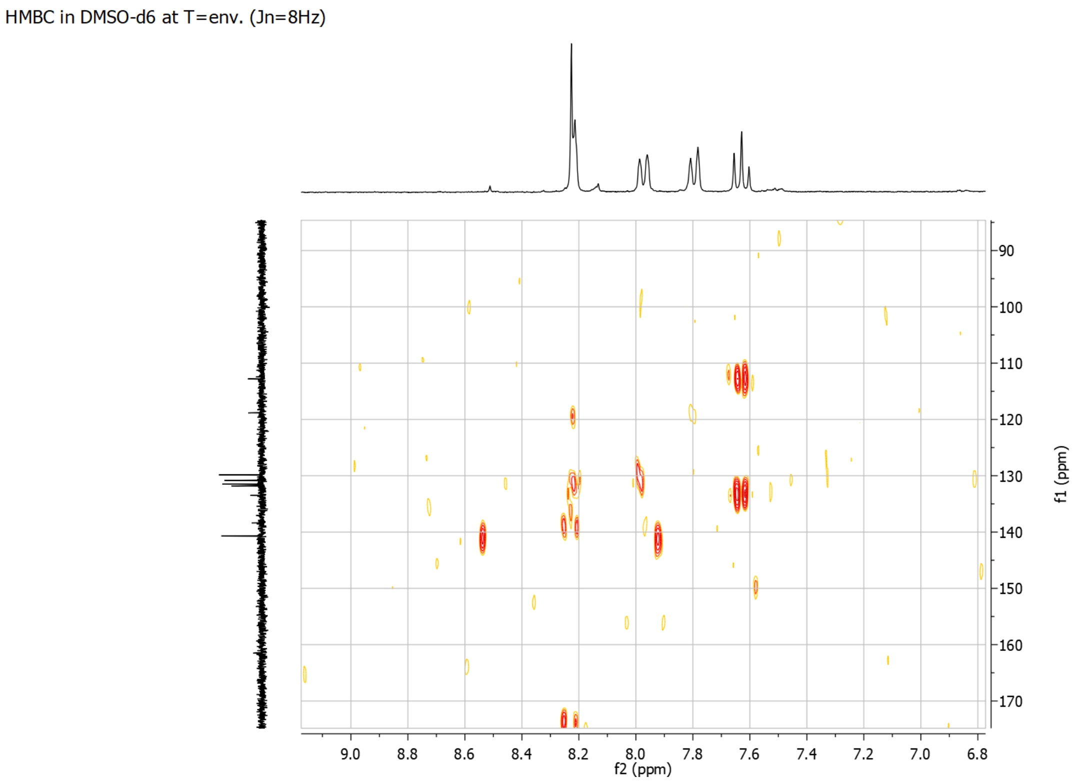

Figure S13. HMBC spectrum of **2a**.

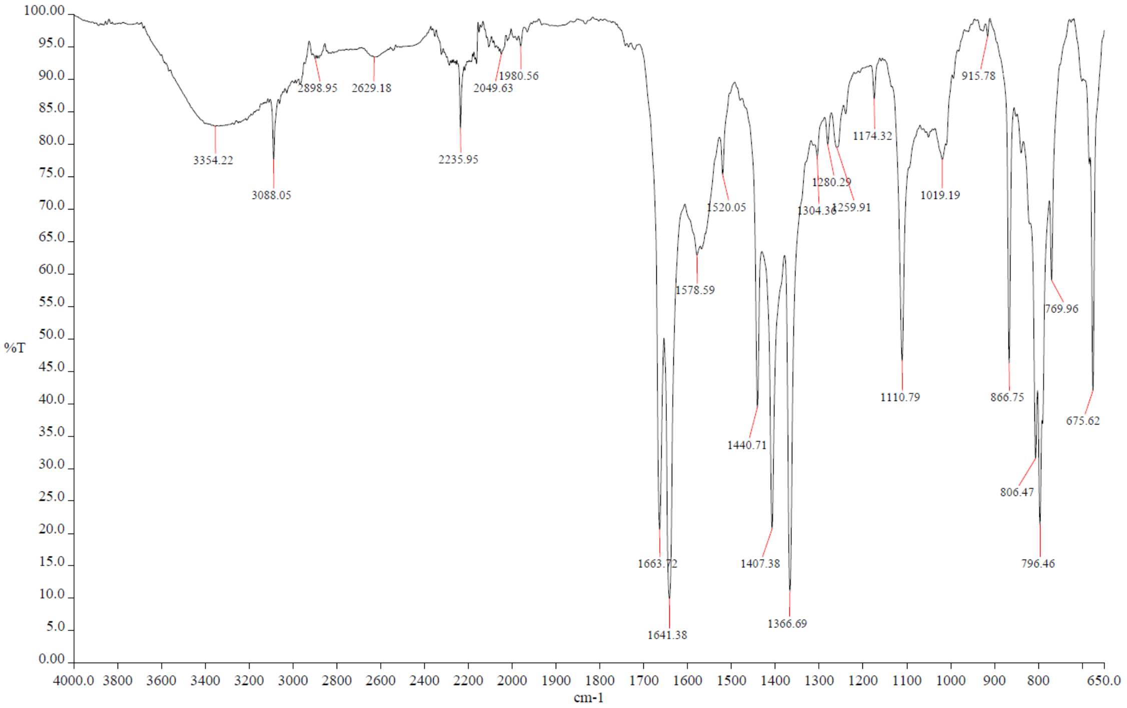

Figure S14. FT-IR spectrum of **2a**.

4. Sodium 5-(4-nitrophenyl)thiazole-2-carboxylate (2b)

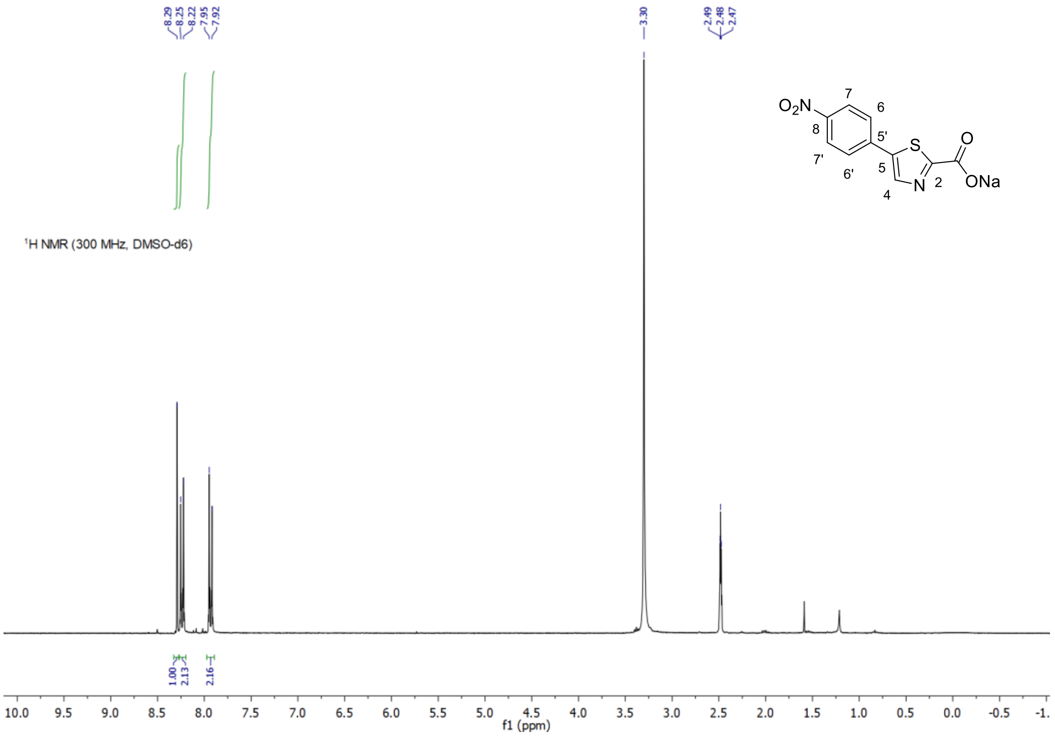

Figure S15. ^{1}H NMR spectrum of **2b**.

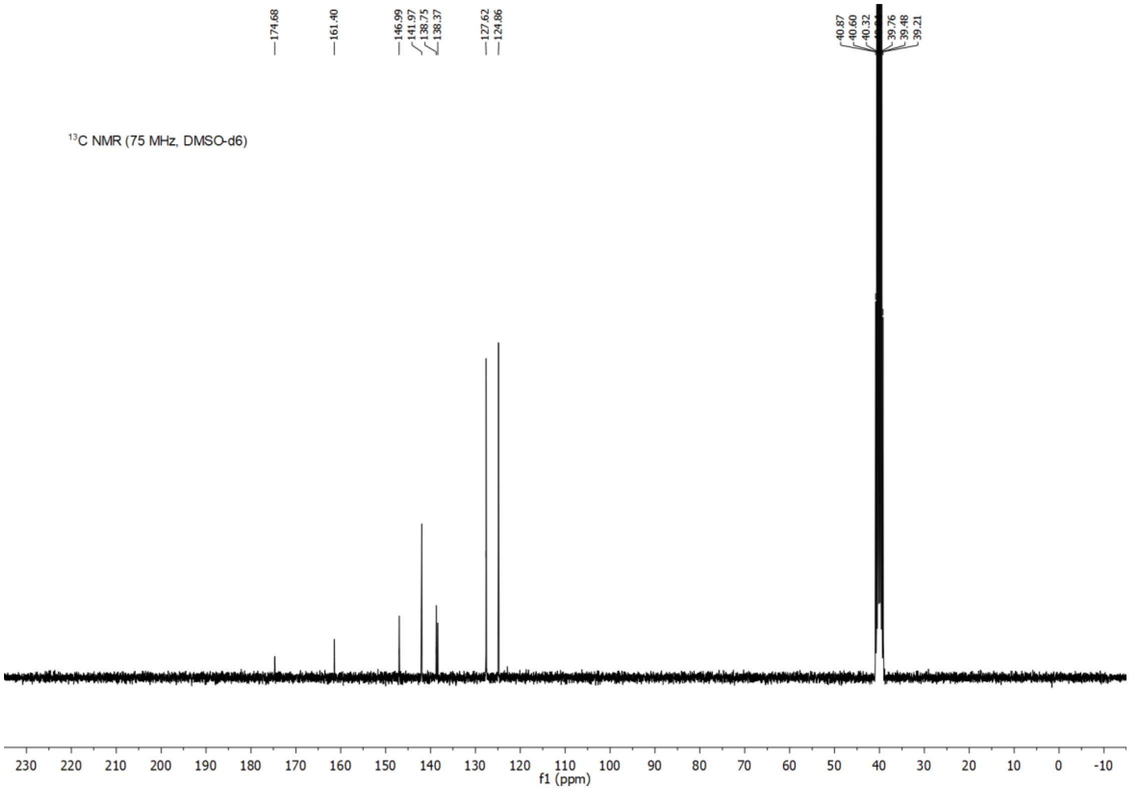

Figure S16. ^{13}C NMR spectrum of **2b**.

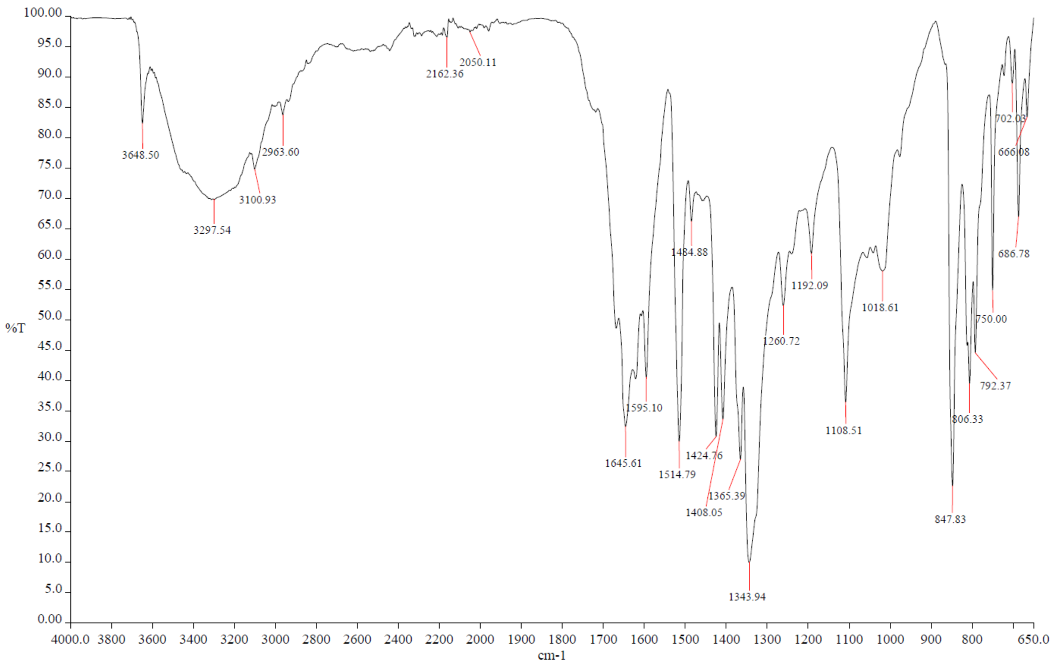

Figure S17. FT-IR spectrum of **2b**.

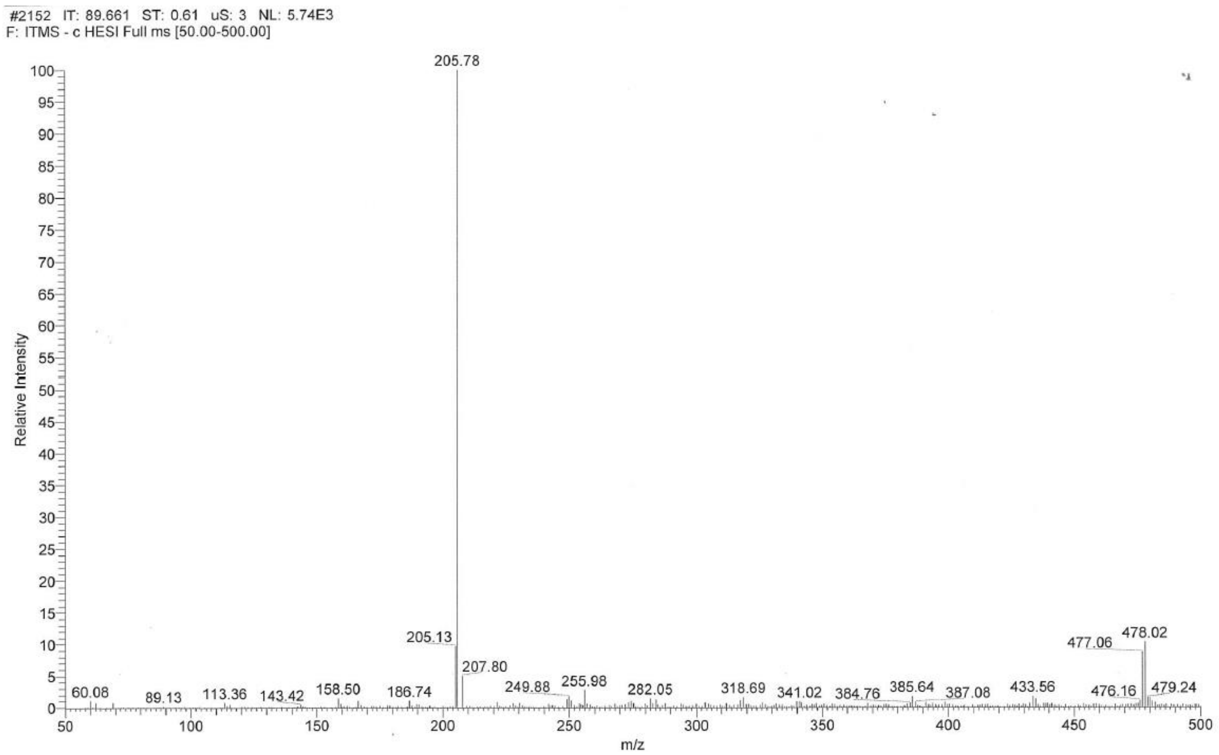

Figure S18. ESI-MS spectrum of **2b**.

5. Sodium 5-(3-cyanophenyl)oxazole-2-carboxylate (3a)

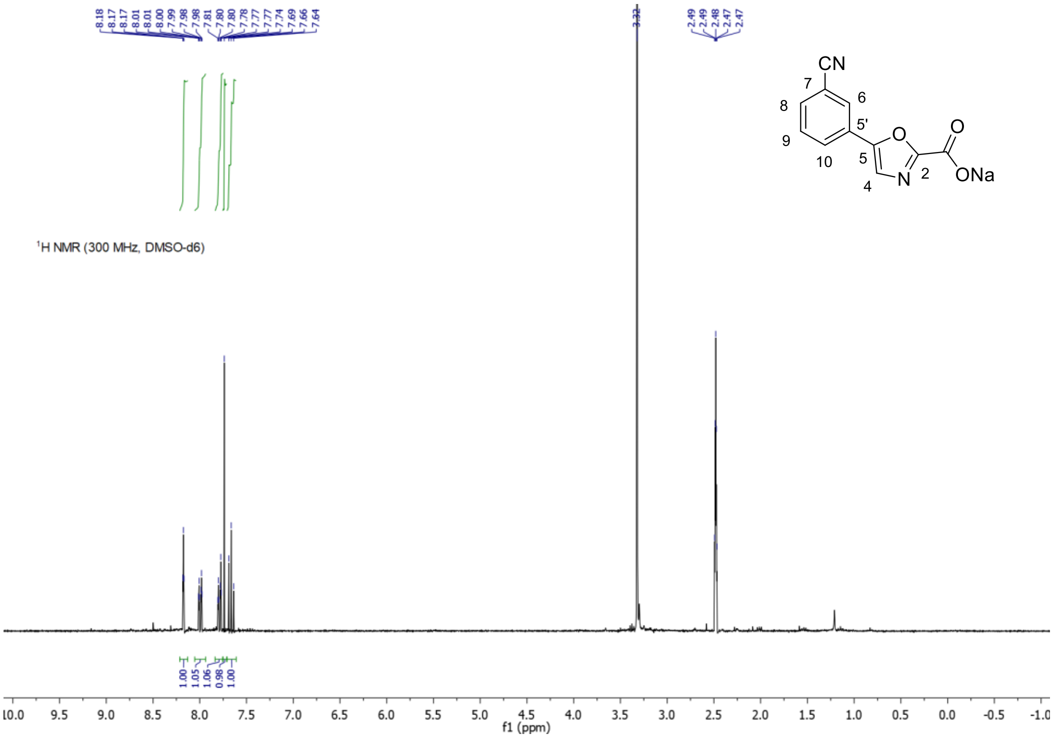

Figure S19. ^{1}H NMR spectrum of **3a**.

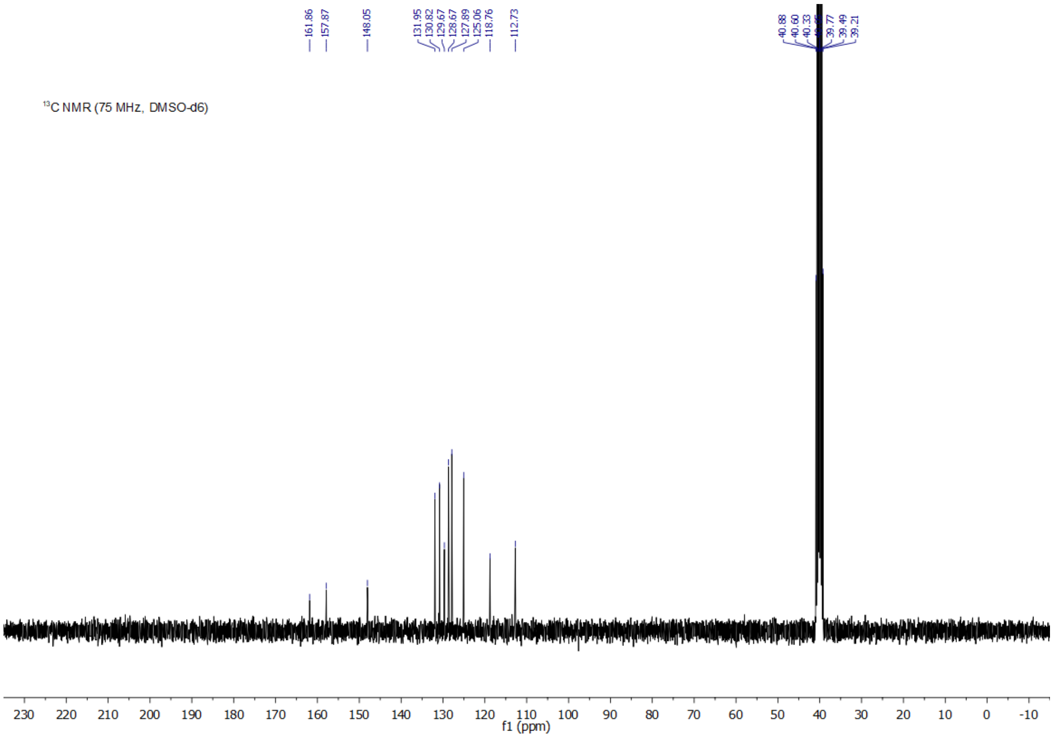

Figure S20. ^{13}C NMR spectrum of **3a**.

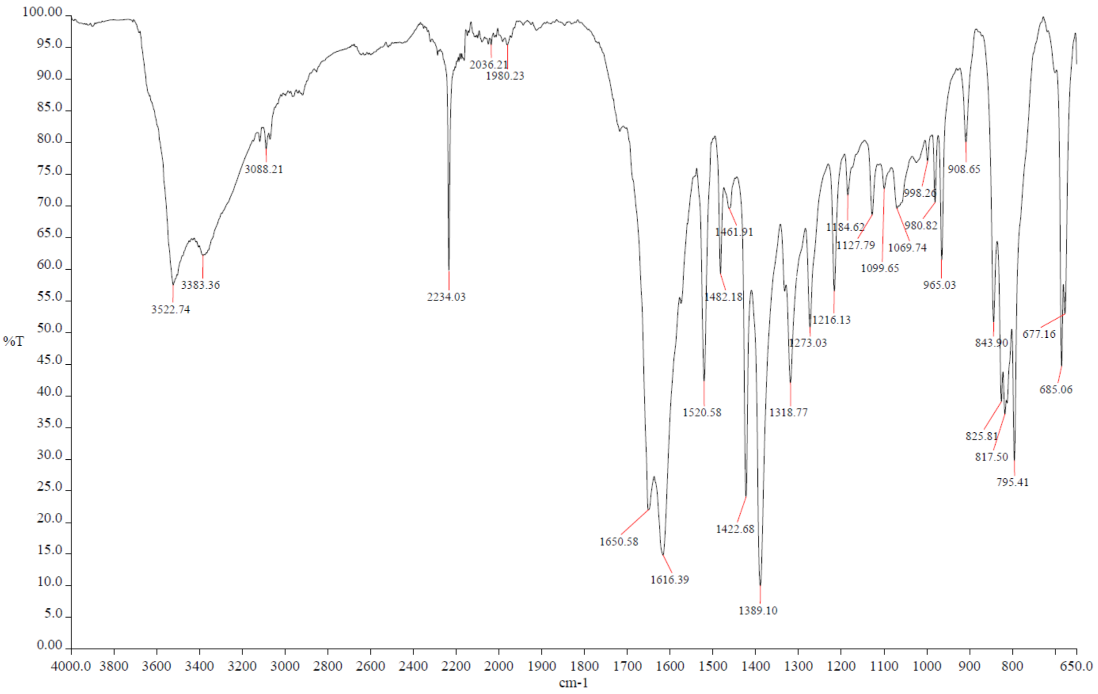

Figure S21. FT-IR spectrum of **3a**.

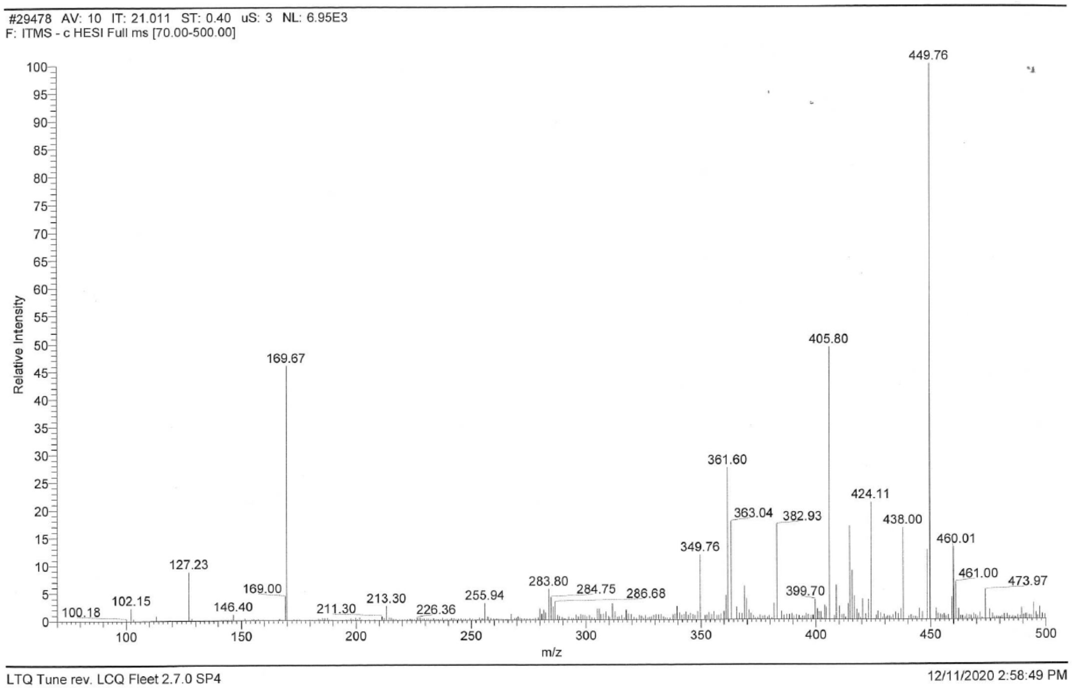

Figure S22. ESI-MS spectrum of **3a**.

6. Sodium 5-(4-nitrophenyl)oxazole-2-carboxylate (3b)

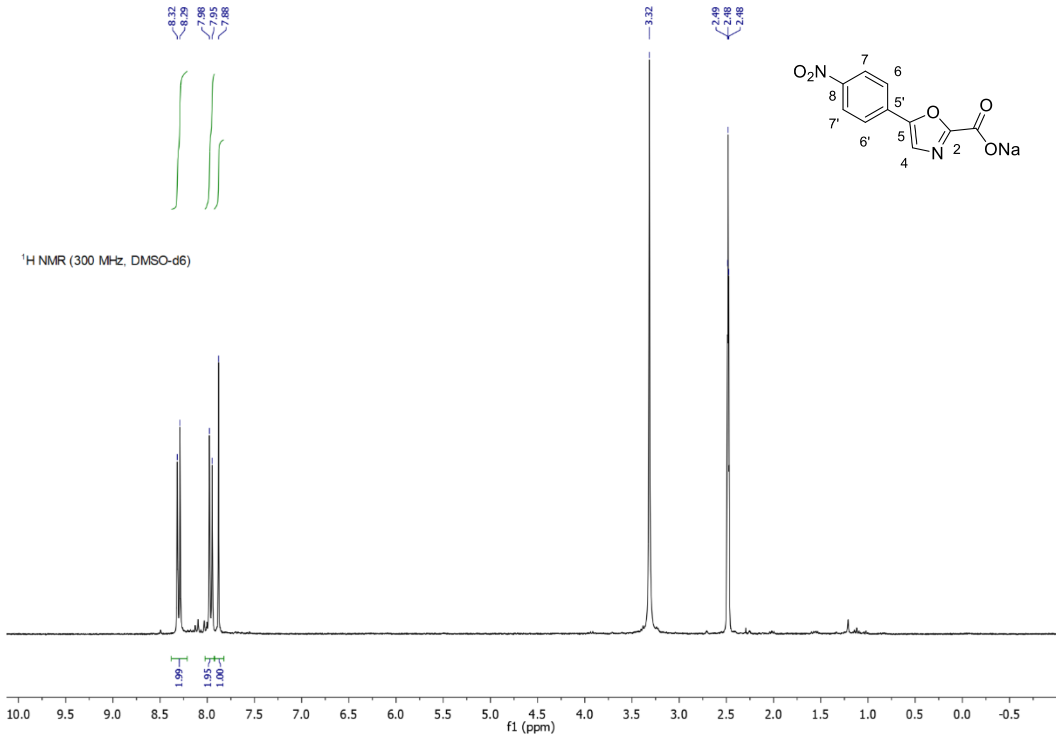

Figure S23. ^{1}H NMR spectrum of **3b**.

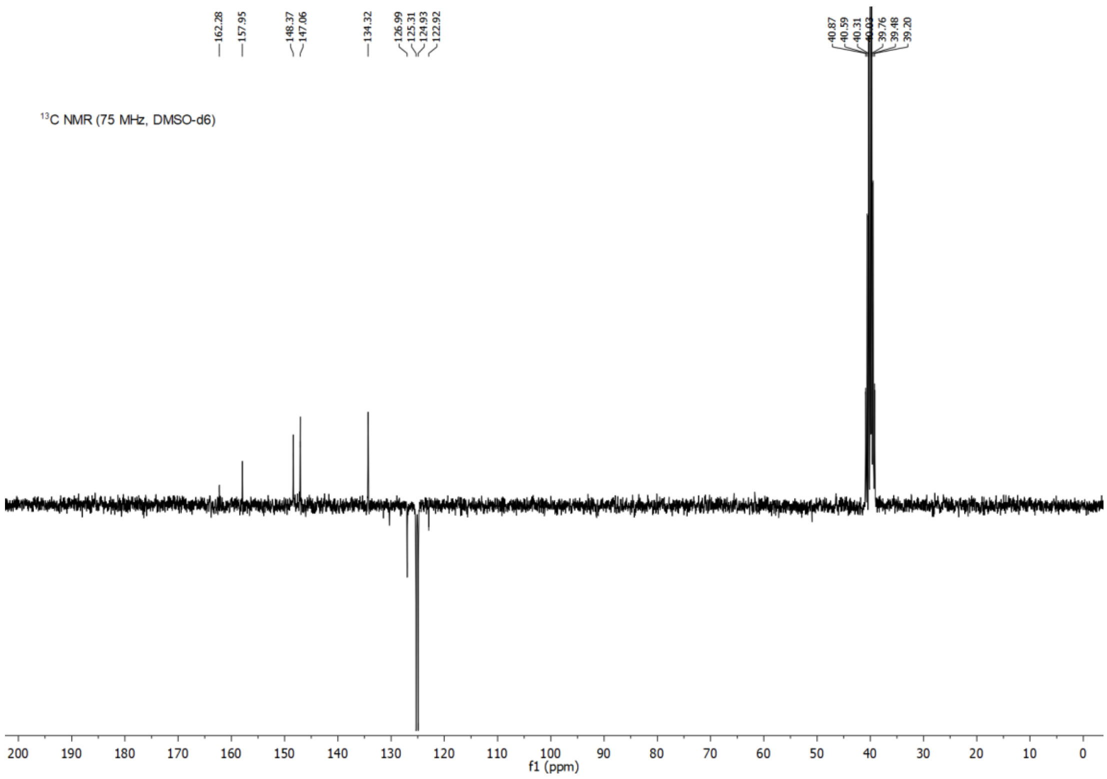

Figure S24. ^{13}C NMR APT spectrum of **3b**.

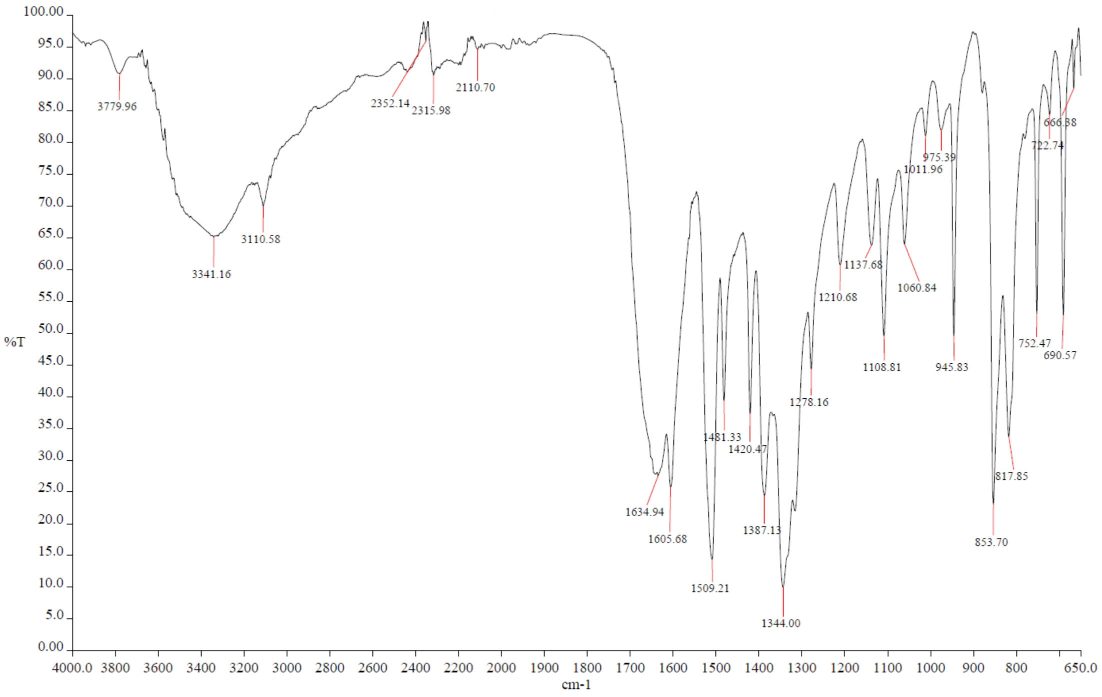

Figure S25. FT-IR spectrum of **3b**.

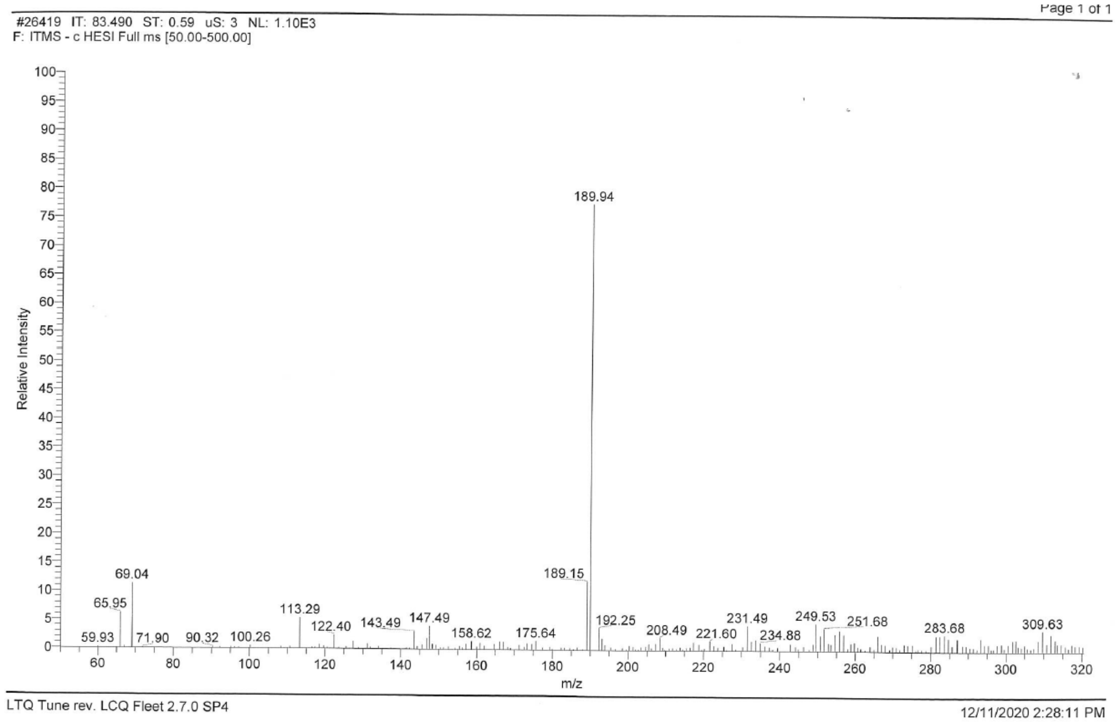

Figure S26. ESI-MS spectrum of **3b**.

7. 5-(3-Cyanophenyl)-1*H*-imidazole-2-carboxylic acid (4a)

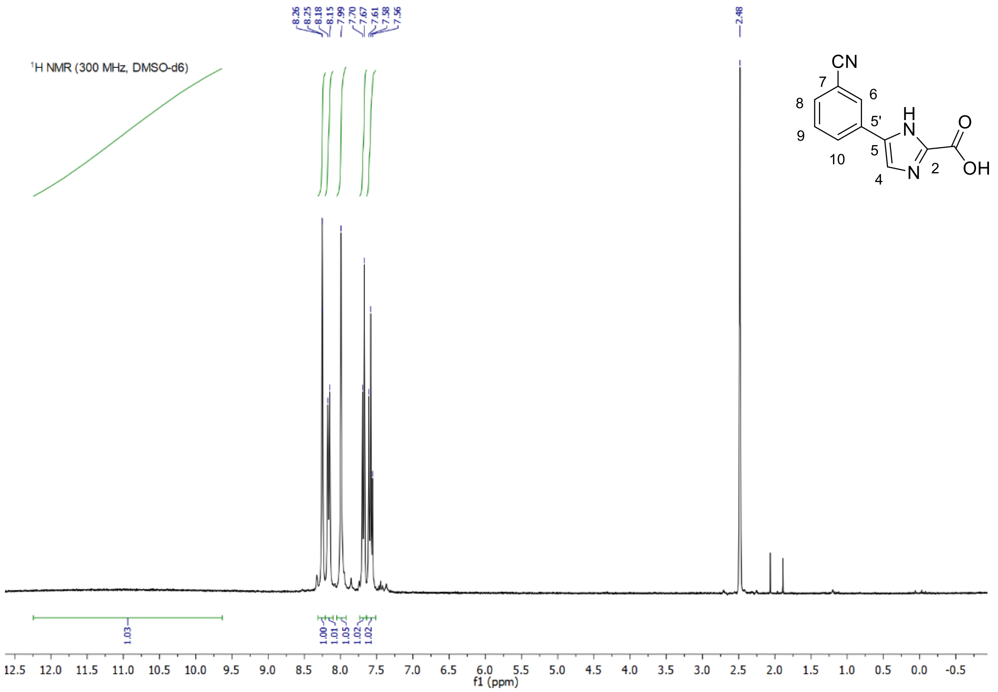

Figure S27. ^{1}H NMR spectrum of **4a**.

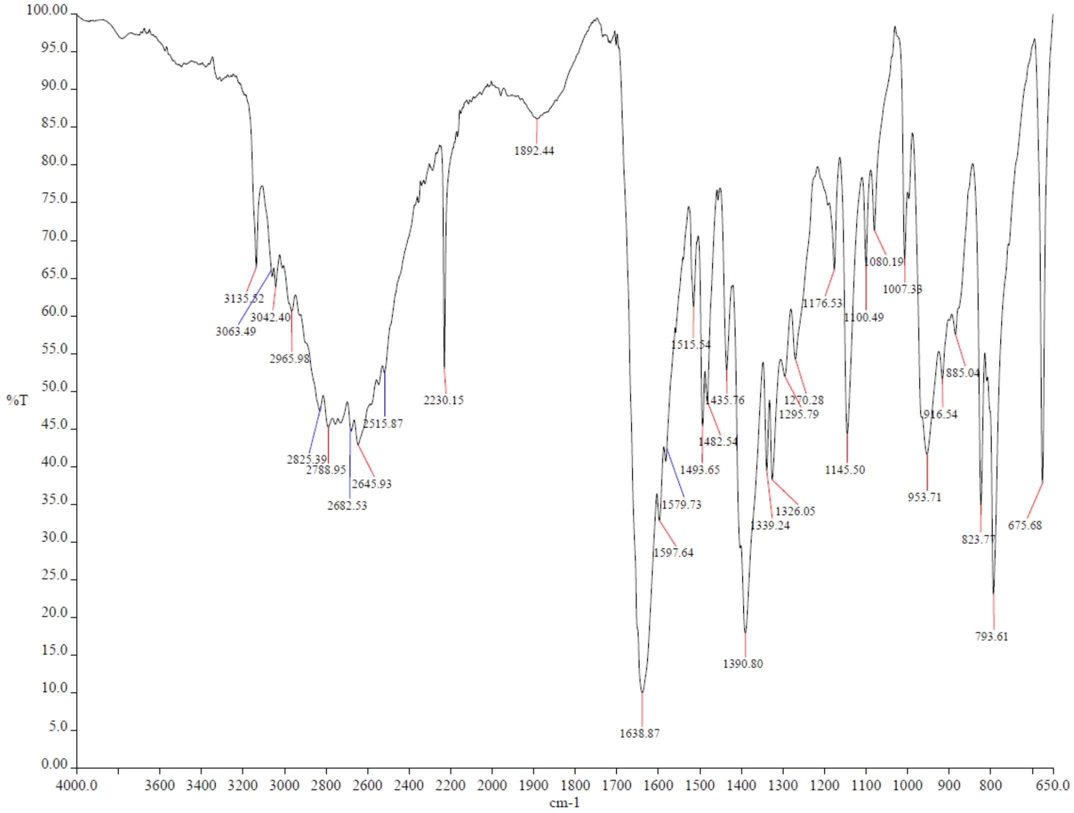

Figure S28. FT-IR spectrum of **4a**.

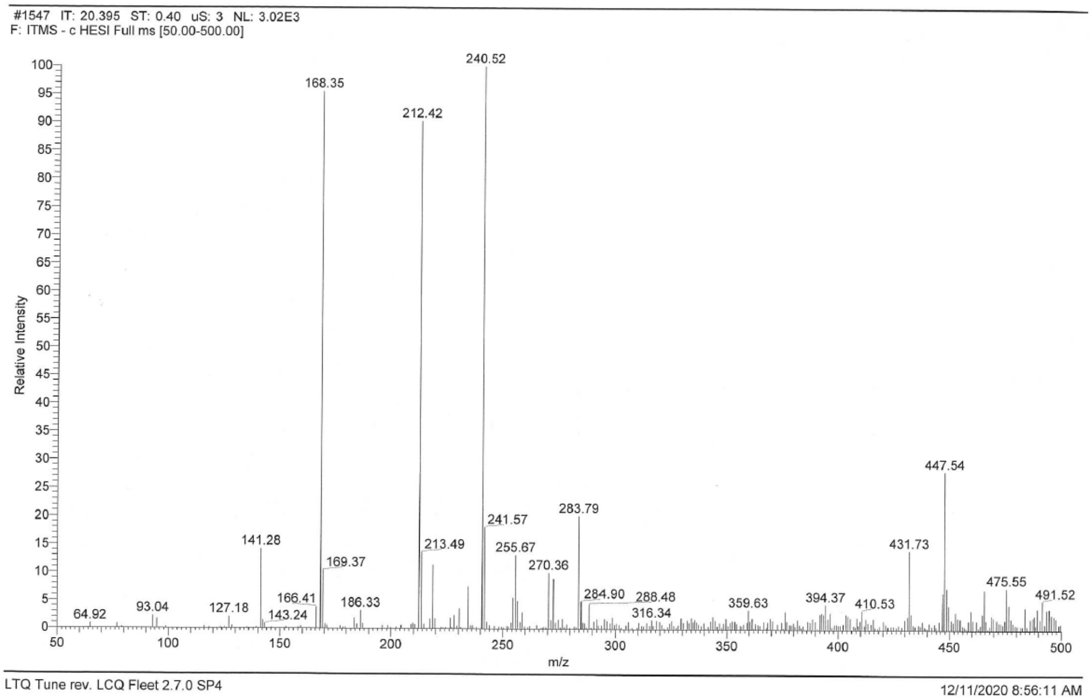

Figure S29. ESI-MS spectrum of **4a**.

8. 5-(4-Nitrophenyl)-1*H*-imidazole-2-carboxylic acid (as sodium salt) (4b)

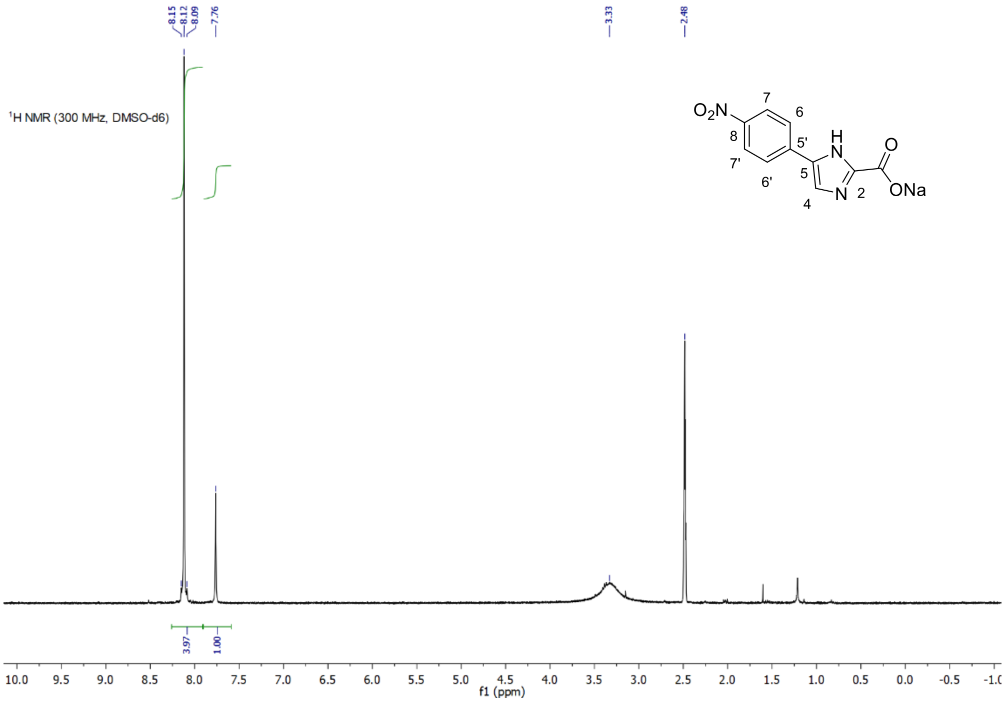

Figure S30. ^{1}H NMR spectrum of **4b**.

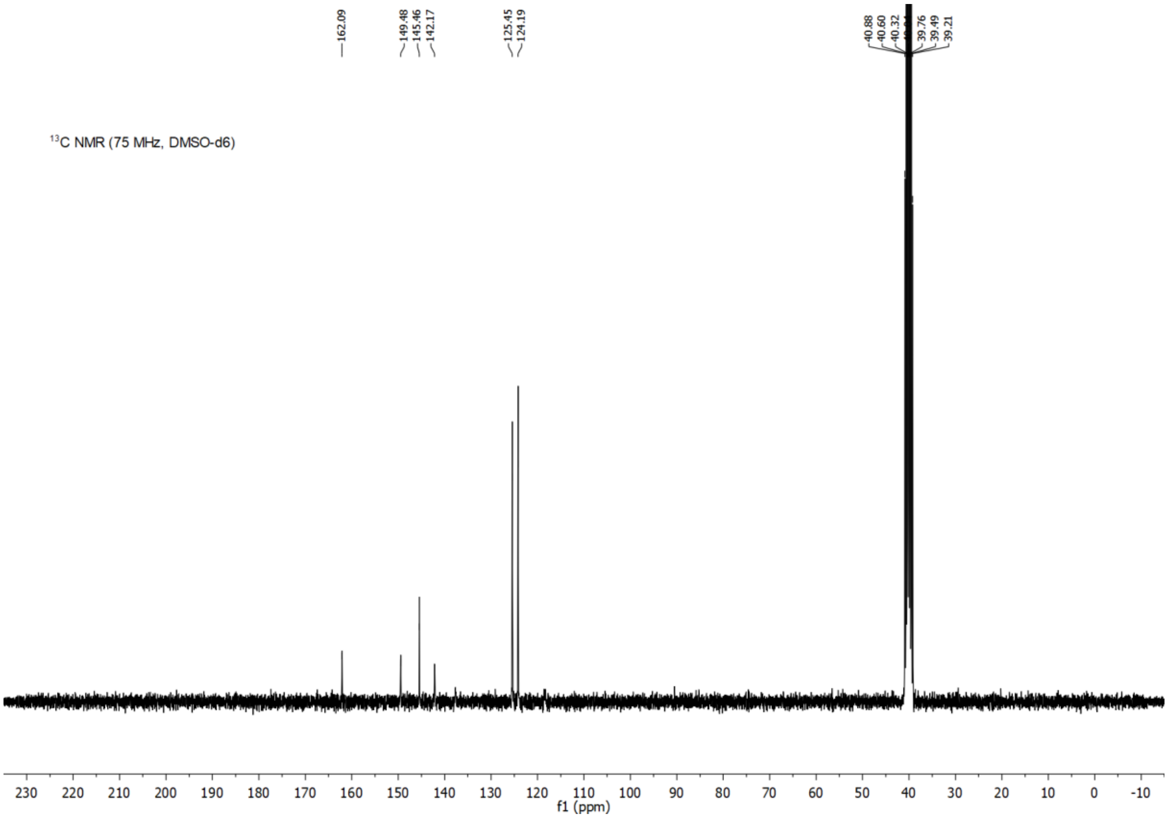

Figure S31. ^{13}C NMR spectrum of **4b**.

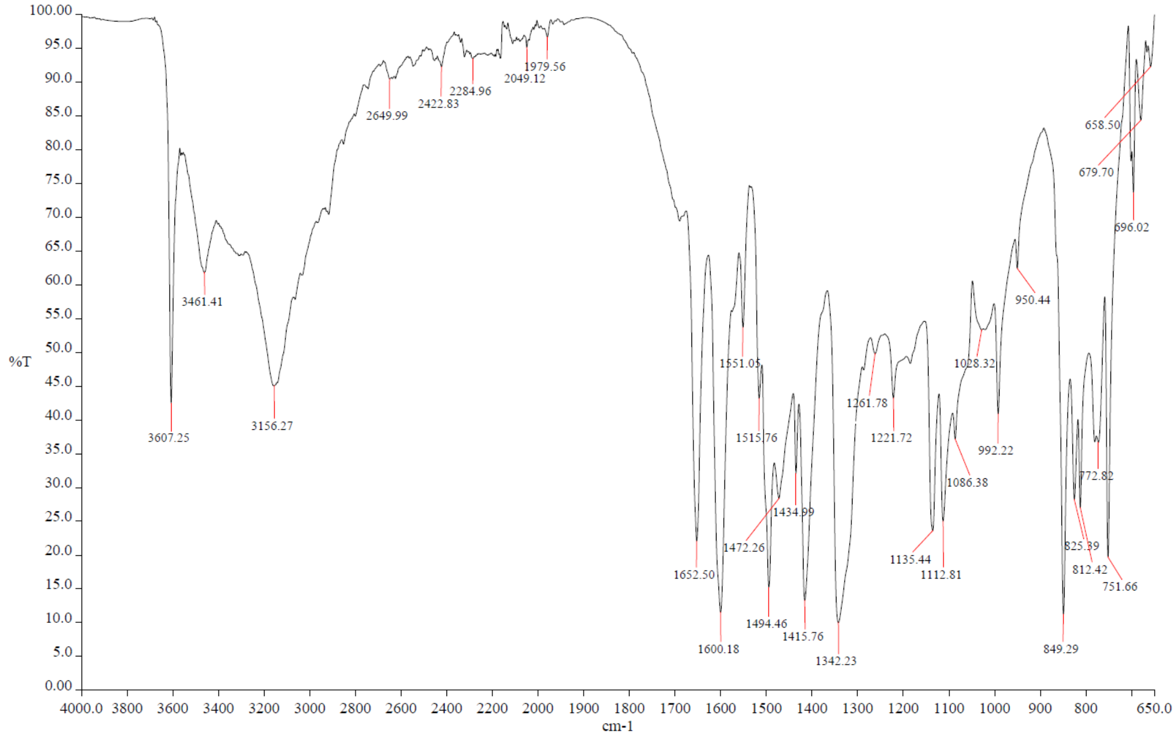

Figure S32. FT-IR spectrum of **4b**.

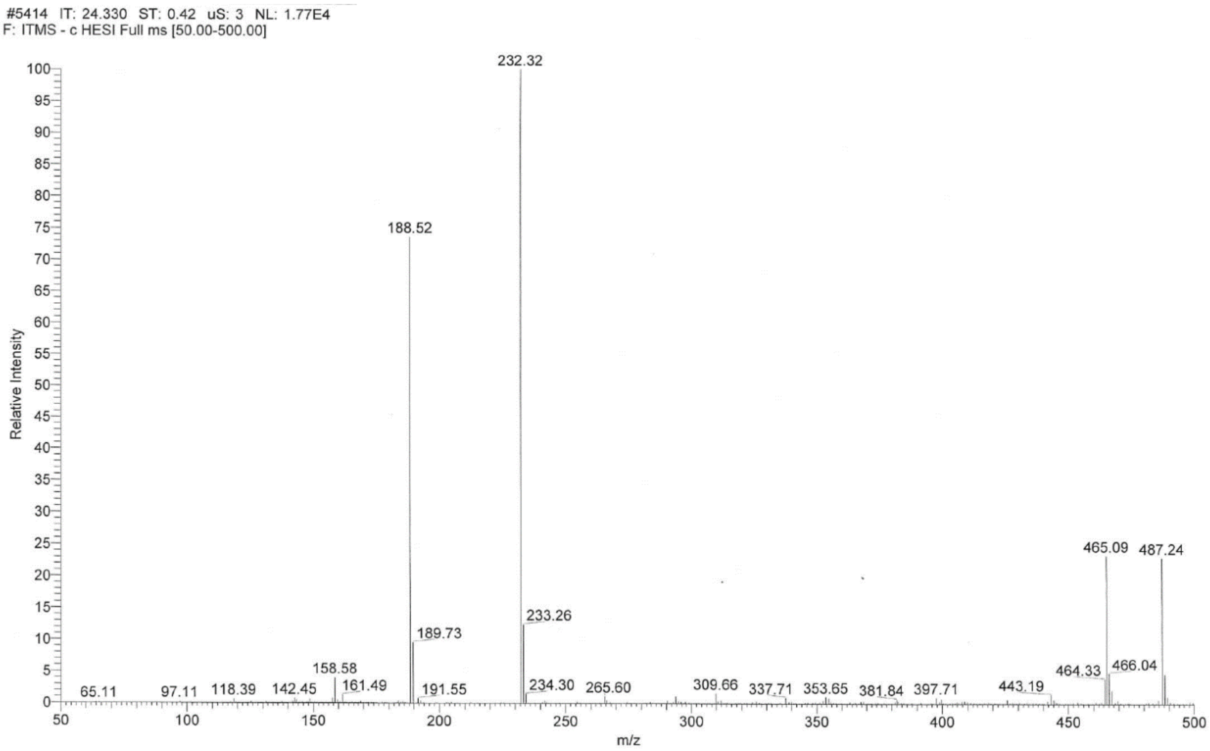

Figure S33. ESI-MS spectrum of **4b**.

9. 5-(3-Cyanophenyl)-1,3,4-oxadiazole-2-carboxylic acid (as sodium salt) (5a)

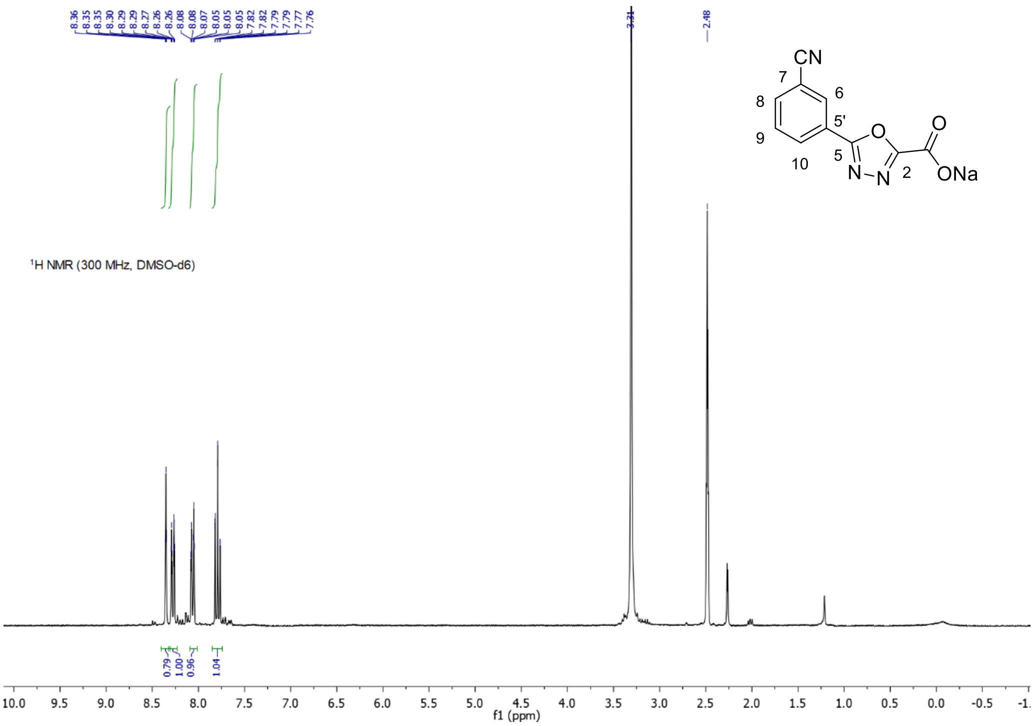

Figure S34. ^{1}H NMR spectrum of **5a**.

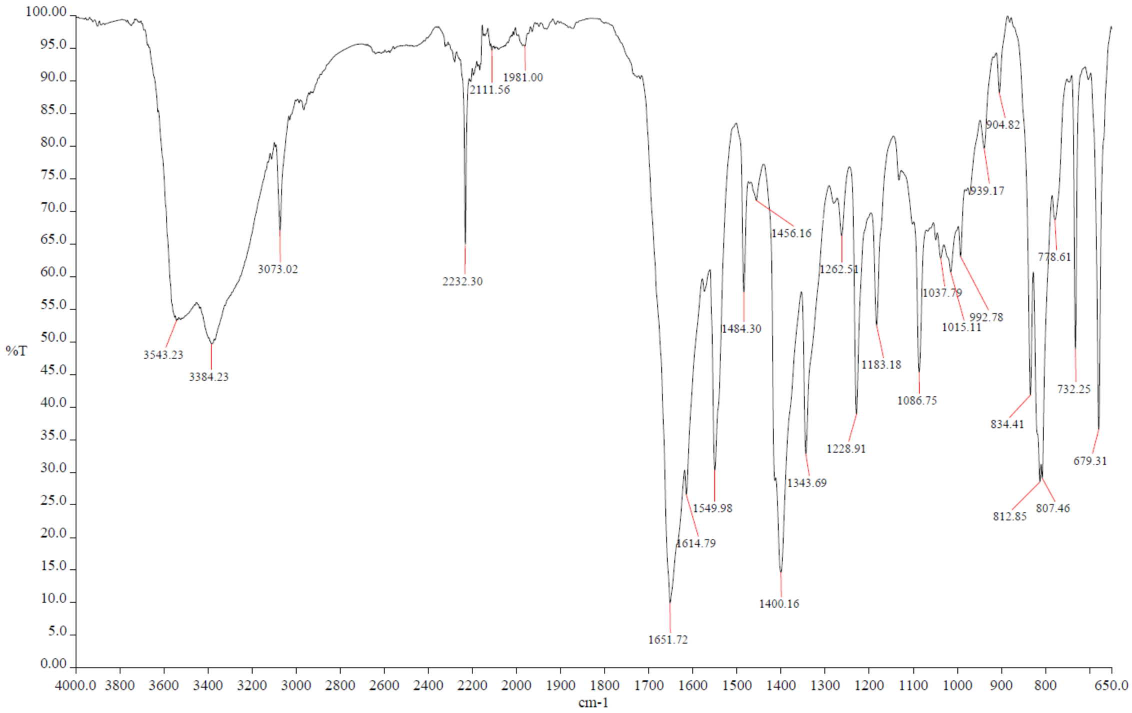

Figure S35. FT-IR spectrum of **5a**.

10. 5-(4-Nitrophenyl)-1,3,4-oxadiazole-2-carboxylic acid (5b)

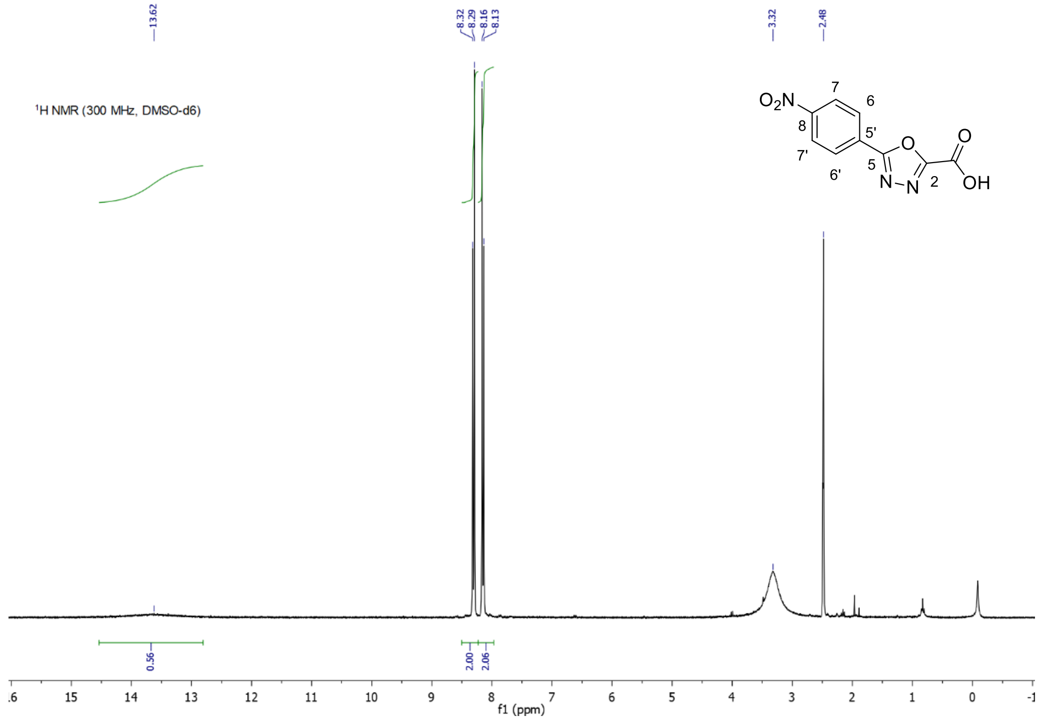

Figure S36. ^{1}H NMR spectrum of **5b**.

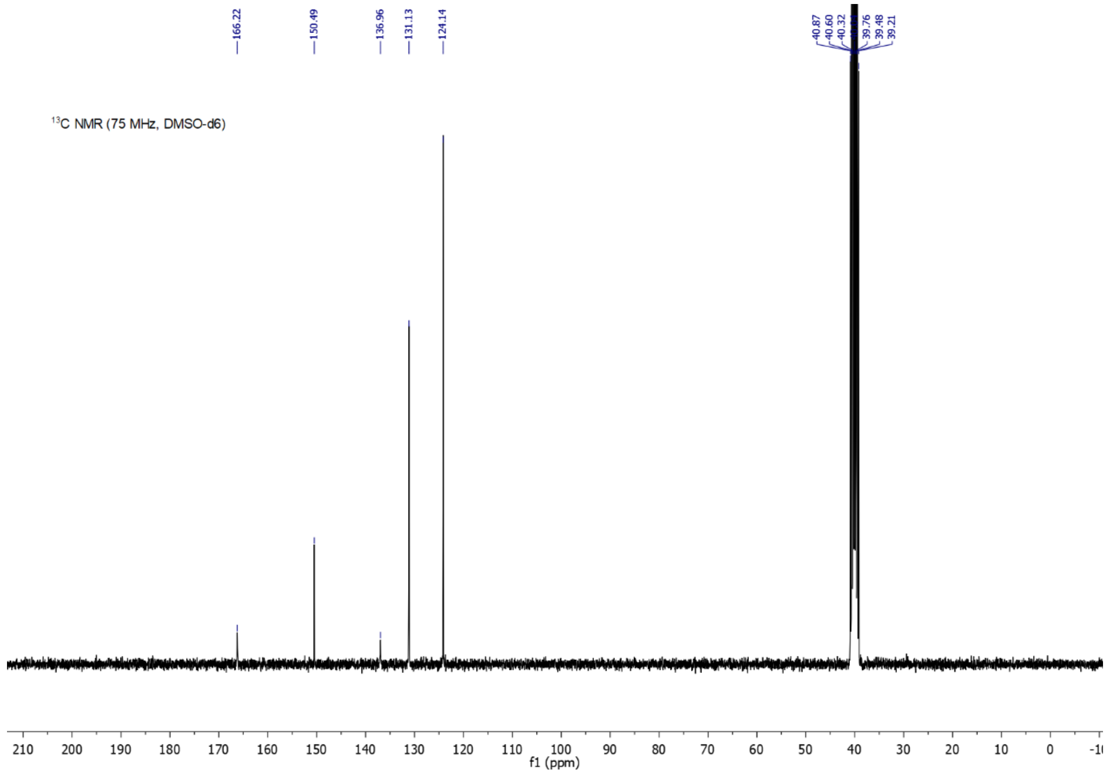

Figure S37. ^{13}C NMR spectrum of **5b**.

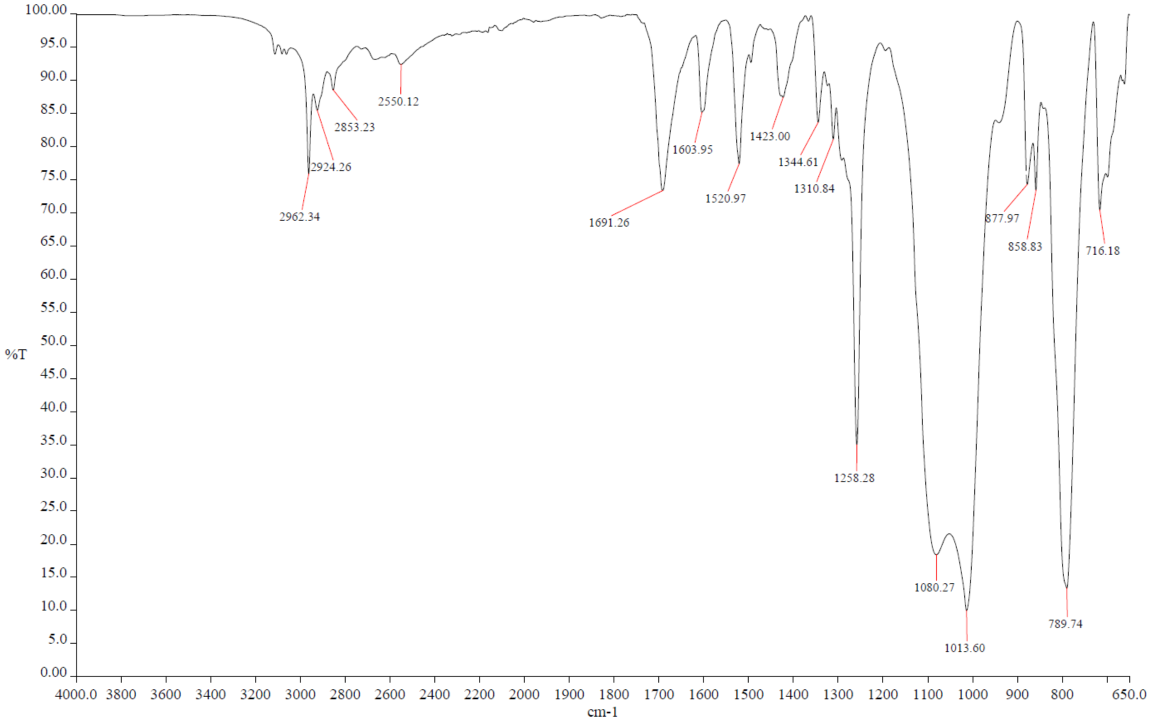

Figure S38. FT-IR spectrum of **5b**.

11. 1-(3-Cyanophenyl)-1*H*-1,2,3-triazole-4-carboxylic acid (6a)

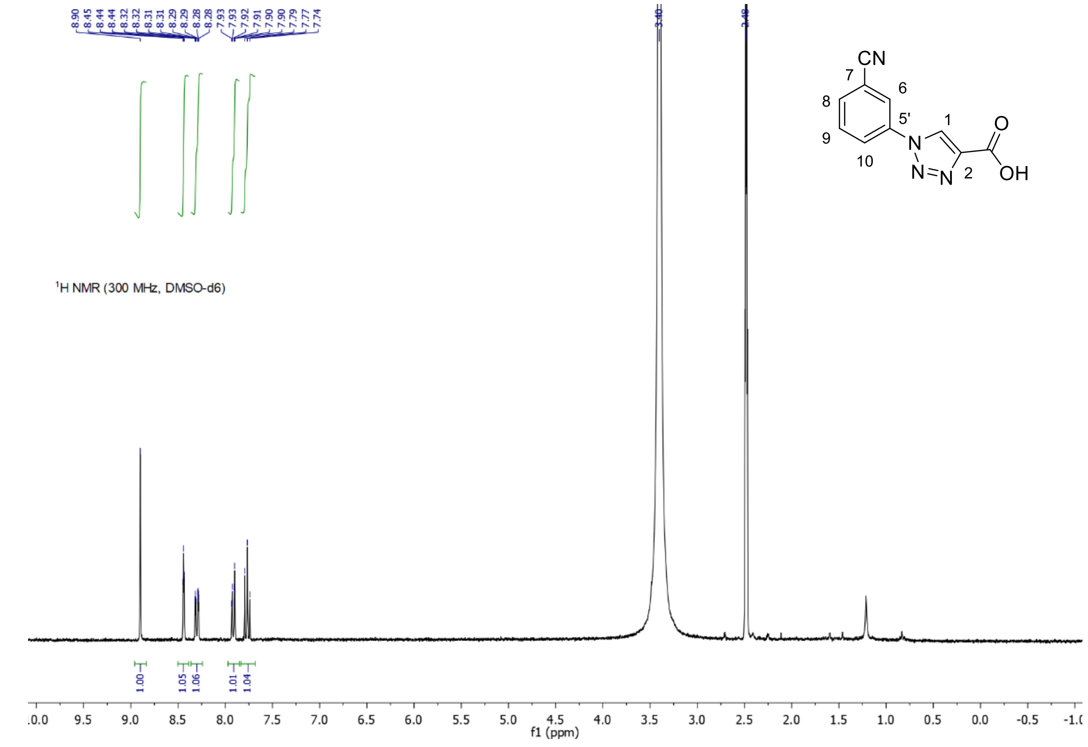

Figure S39. ^{1}H NMR spectrum of **6a**.

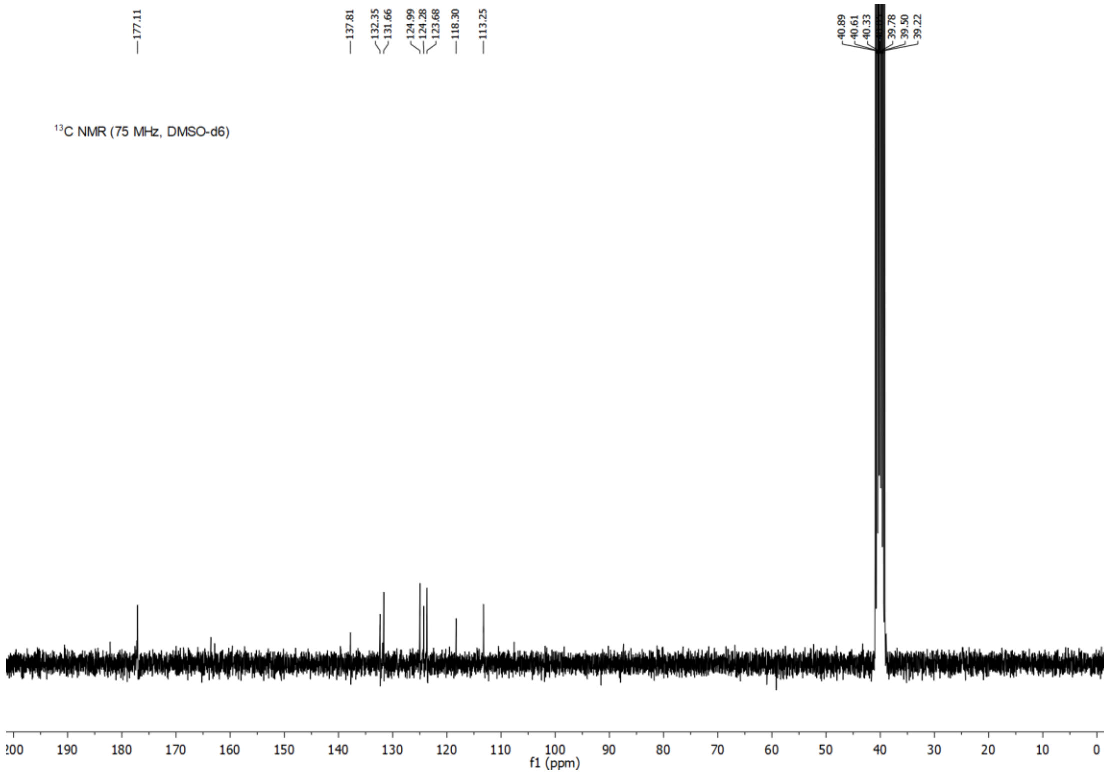

Figure S40. ^{13}C NMR spectrum of **6a**.

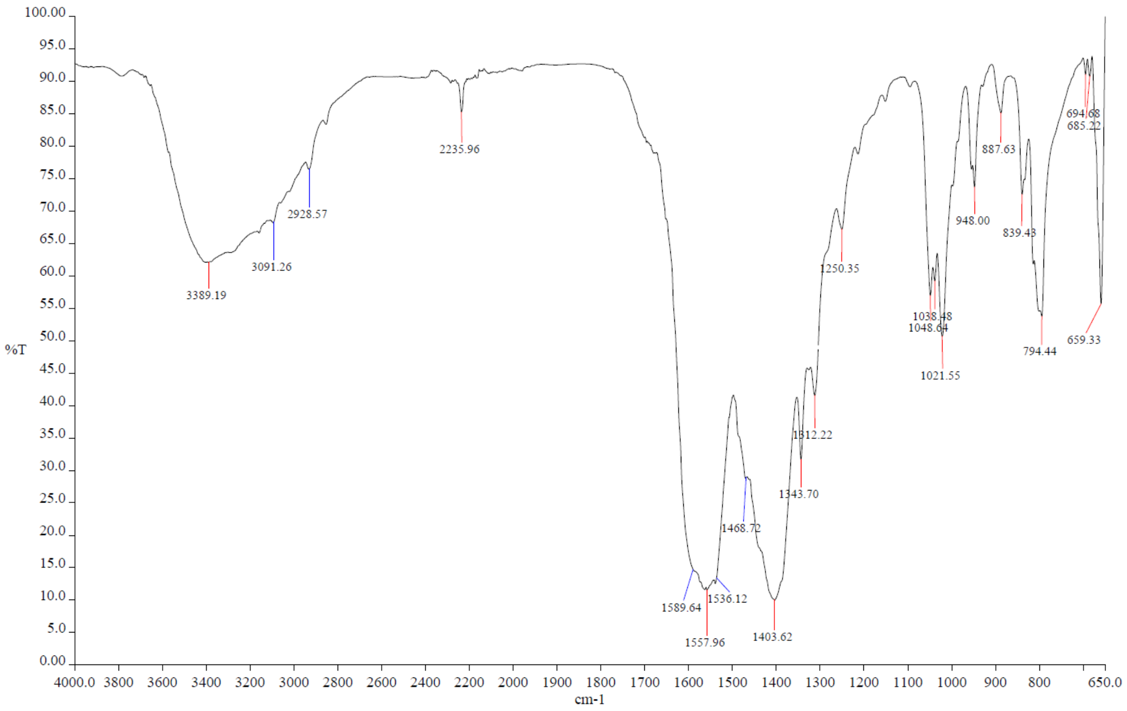

Figure S41. FT-IR spectrum of **6a**.

12. 1-(4-Nitrophenyl)-1*H*-1,2,3-triazole-4-carboxylic acid (6b)

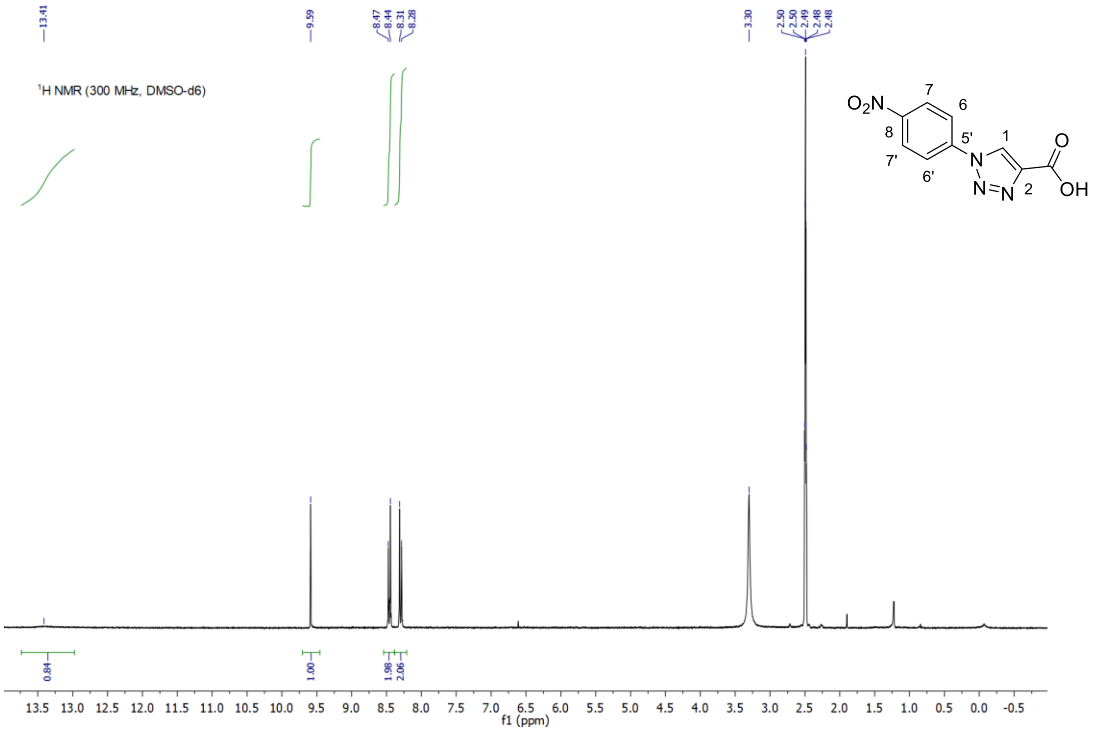

Figure S42. ^{1}H NMR spectrum of **6b**.

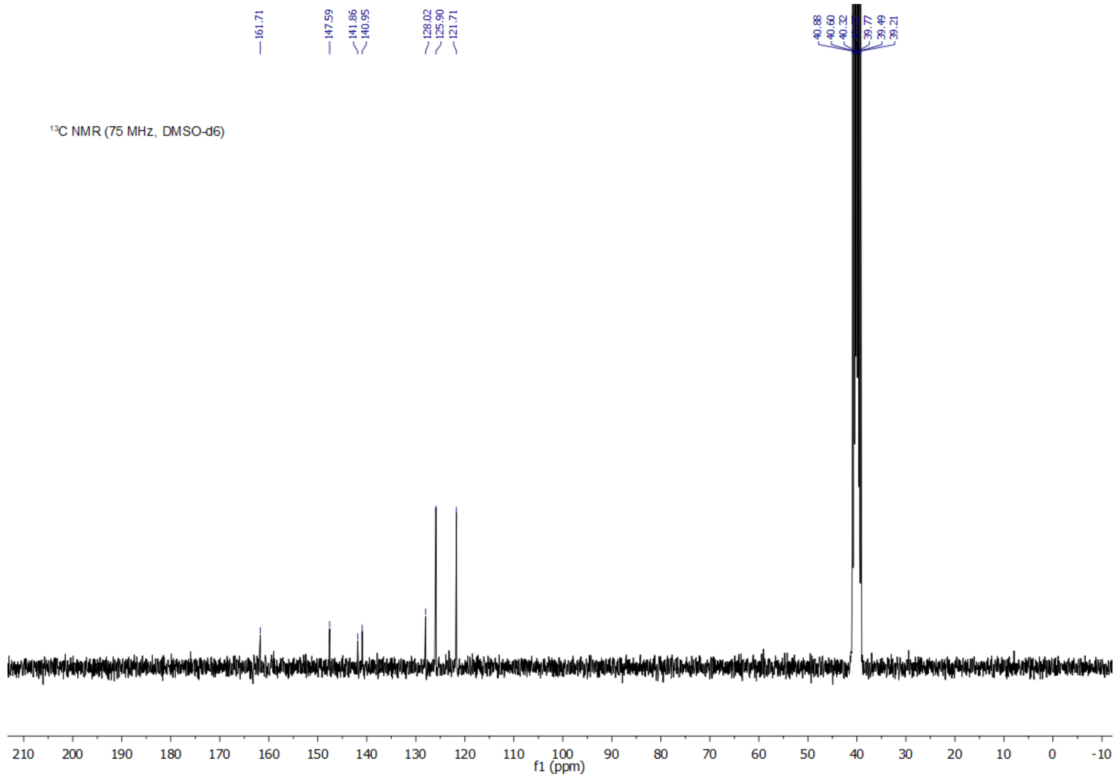

Figure S43. ^{13}C NMR spectrum of **6b**.

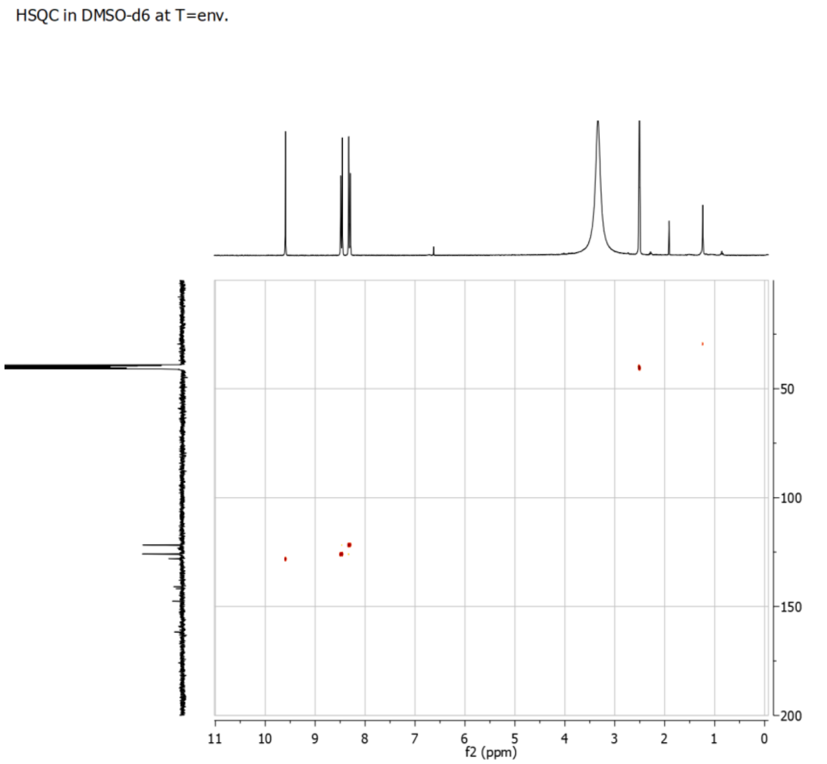

	Parameter	Value
1	Data File Name	C:/ Users/ Donatella/ Desktop/ NMR/ 300MHz - Americo/ PIN_FL102/ 4/ ser
2	Title	PIN_FL102.4.ser
3	Comment	FL 102 HSQC in DMSO a T=amb.
4	Origin	Bruker BioSpin GmbH
5	Owner	nmr
6	Site	
7	Instrument	spect
8	Author	
9	Solvent	DMSO
10	Temperature	300.0
11	Pulse Sequence	hsqcetgpsi2
12	Experiment	HSQC
13	Probe	5 mm QNP 1H/ 13C/ 31P/ 19F Z-grad Z8352/ 0036
14	Number of Scans	32
15	Receiver Gain	46300.0
16	Relaxation Delay	2.0000
17	Pulse Width	11.7500
18	Presaturation Frequency	
19	Acquisition Time	0.3072
20	Acquisition Date	2020-08-04T15:33:52
21	Modification Date	2020-08-05T09:37:00
22	Class	
23	Spectrometer Frequency	(300.13, 75.48)
24	Spectral Width	(3333.3, 15095.1)
25	Lowest Frequency	(-26.4, -0.8)
26	Nucleus	(1H, 13C)
27	Acquired Size	(1024, 256)
28	Spectral Size	(2048, 2048)
29	Digital Resolution	(1.63, 7.37)

Figure S44. HSQC spectrum of **6b**.

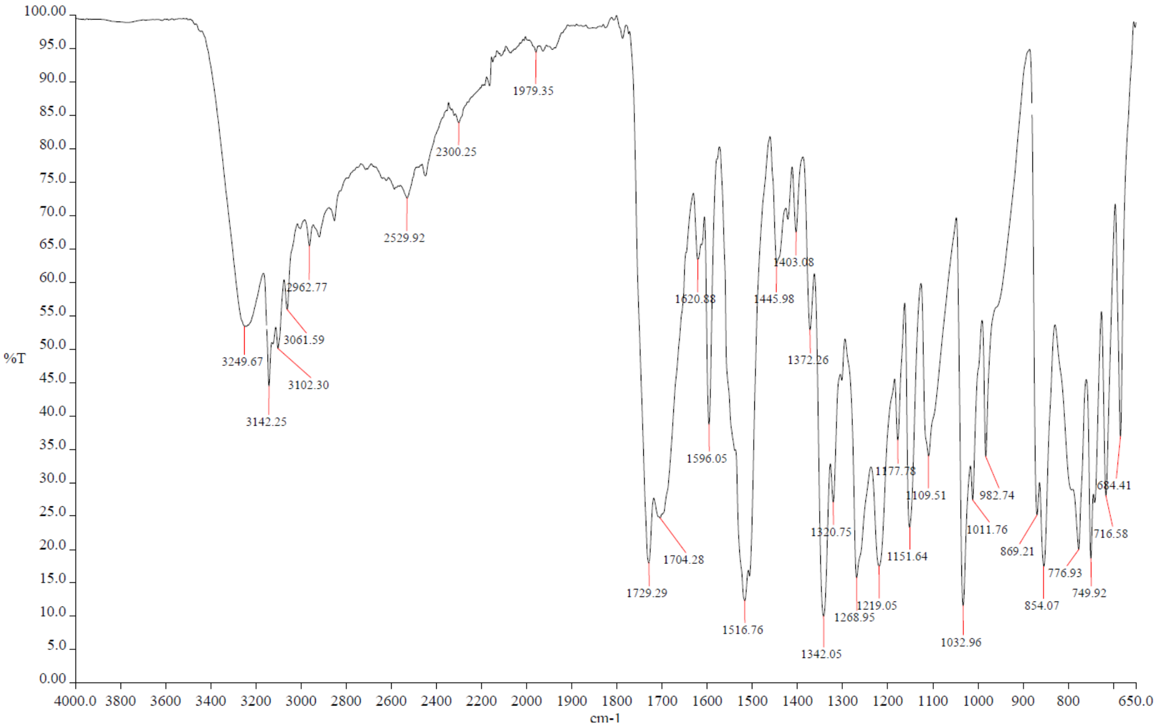

Figure S45. FT-IR spectrum of **6b**.

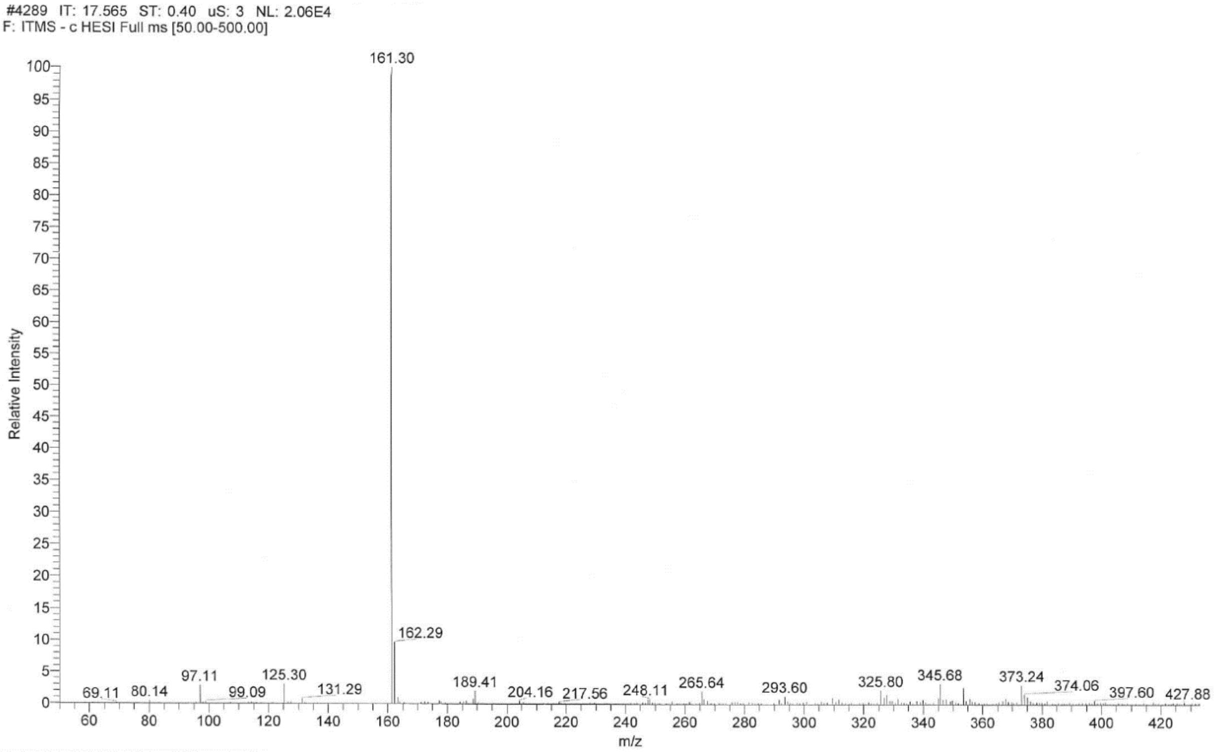

Figure S46. ESI-MS spectrum of **6b**.

13. Methyl 5-bromothiophene-2-carboxylate (7)

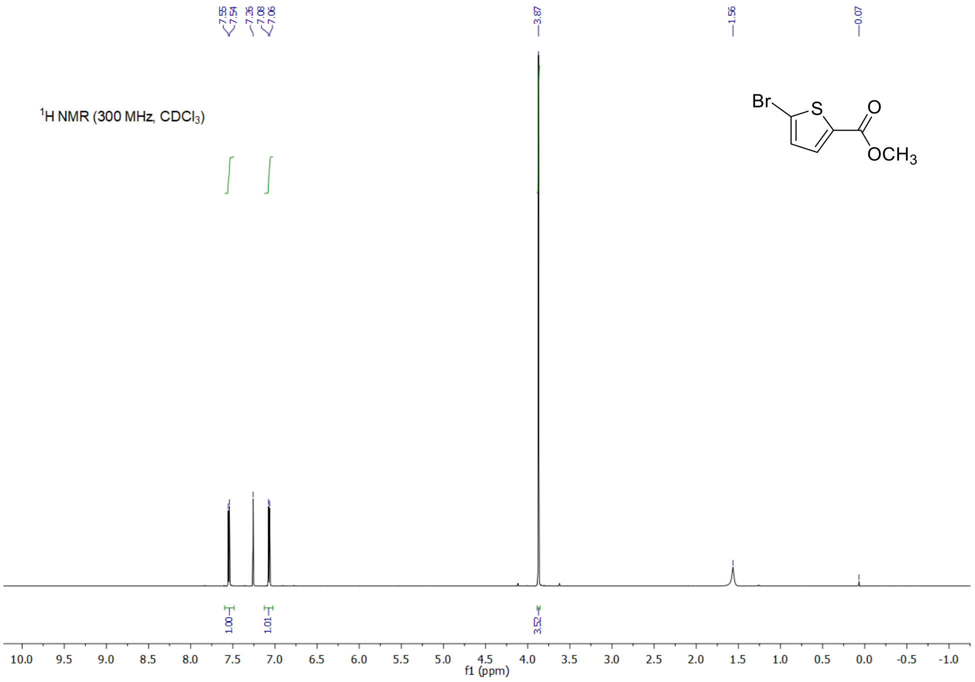

Figure S47. ^{1}H NMR spectrum of **7**.

14. Methyl 5-(3-cyanophenyl)thiophene-2-carboxylate (8a)

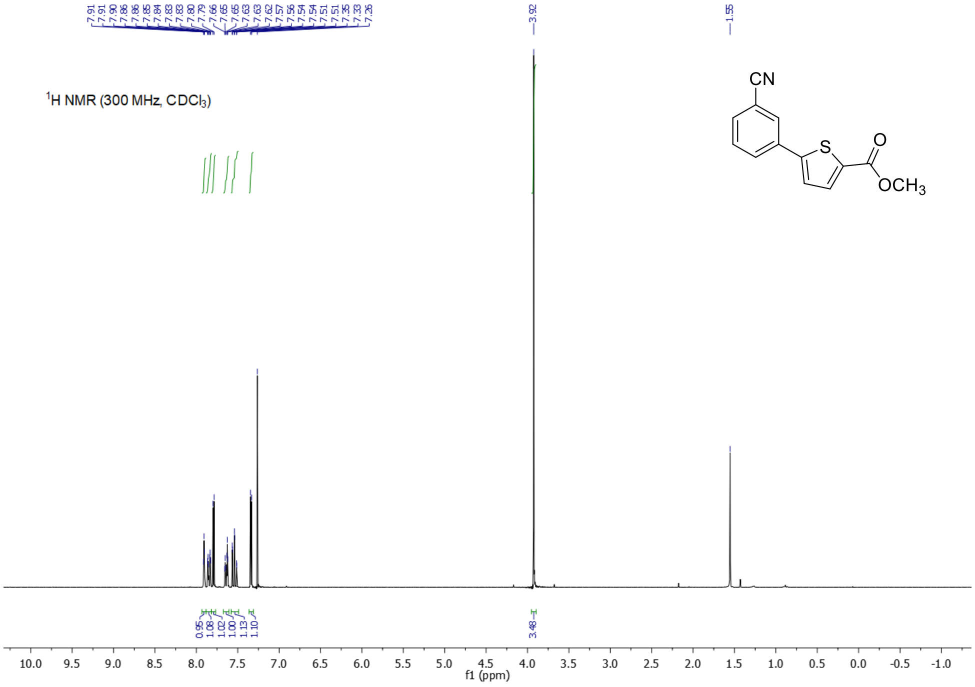

Figure S48. ^{1}H NMR spectrum of **8a**.

15. Methyl 5-(4-nitrophenyl)thiophene-2-carboxylate (8b)

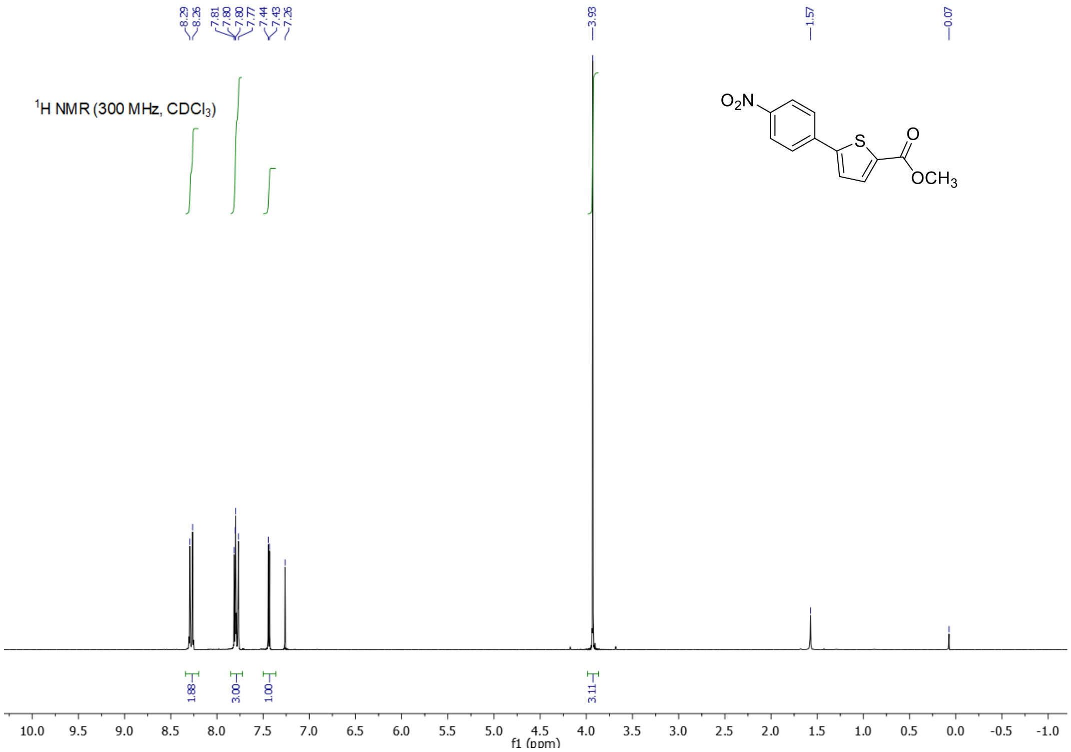

Figure S49. ^{1}H NMR spectrum of **8b**.

16. 3-(2-Bromoacetyl)benzonitrile (9a)

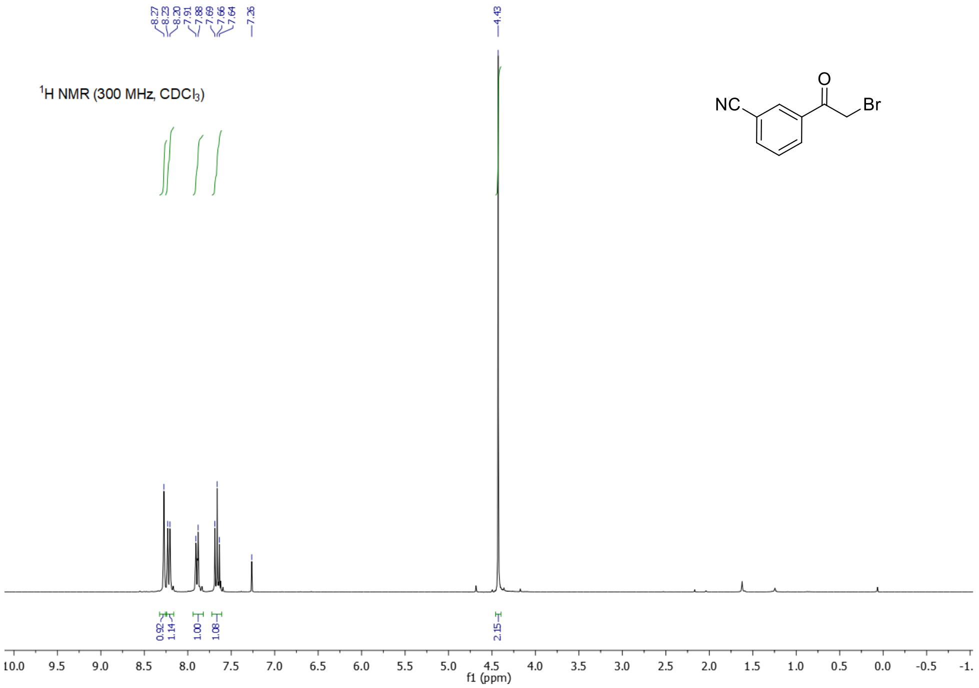

Figure S50. ^{1}H NMR spectrum of **9a**.

17. 2-Bromo-1-(4-nitrophenyl)ethanone (9b)

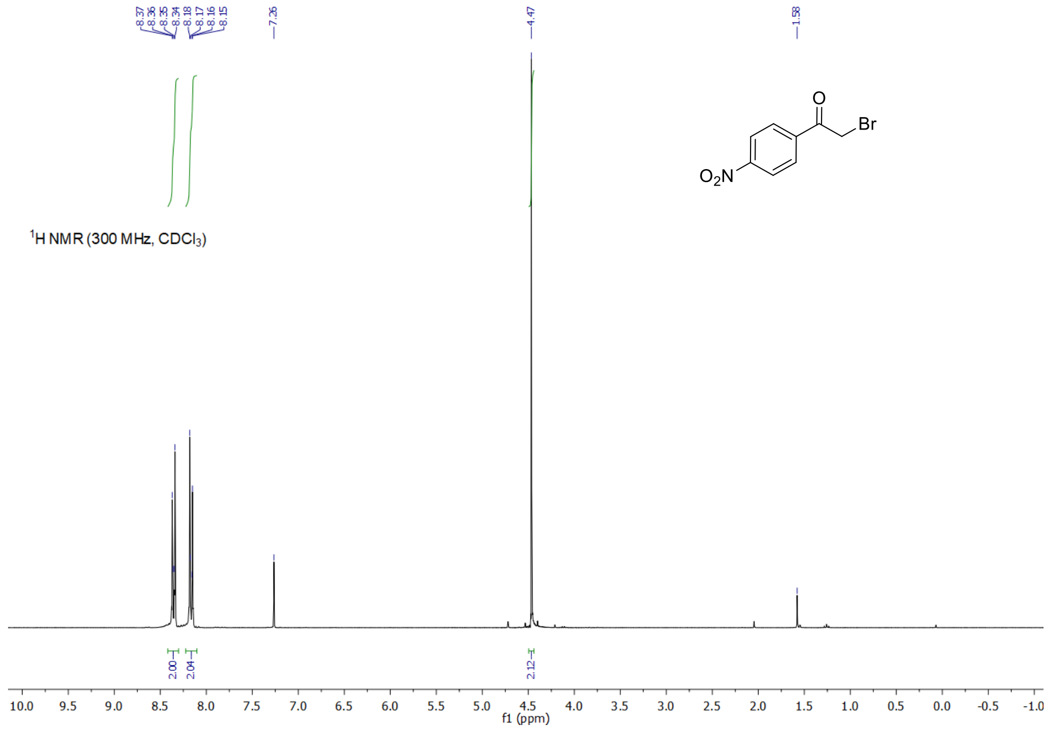

Figure S51. ^{1}H NMR spectrum of **9b**.

18. 2-(3-Cyanophenyl)-2-oxoethanaminium chloride (10a)

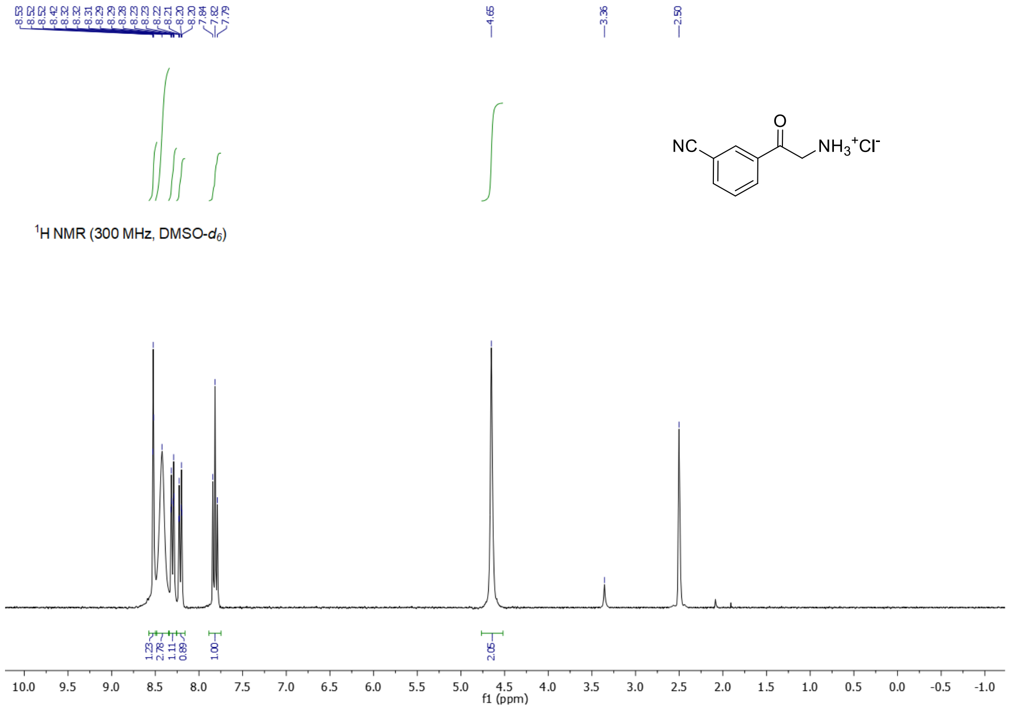

Figure S52. ^{1}H NMR spectrum of **10a**.

19. 2-(4-Nitrophenyl)-2-oxoethanaminium chloride (10b)

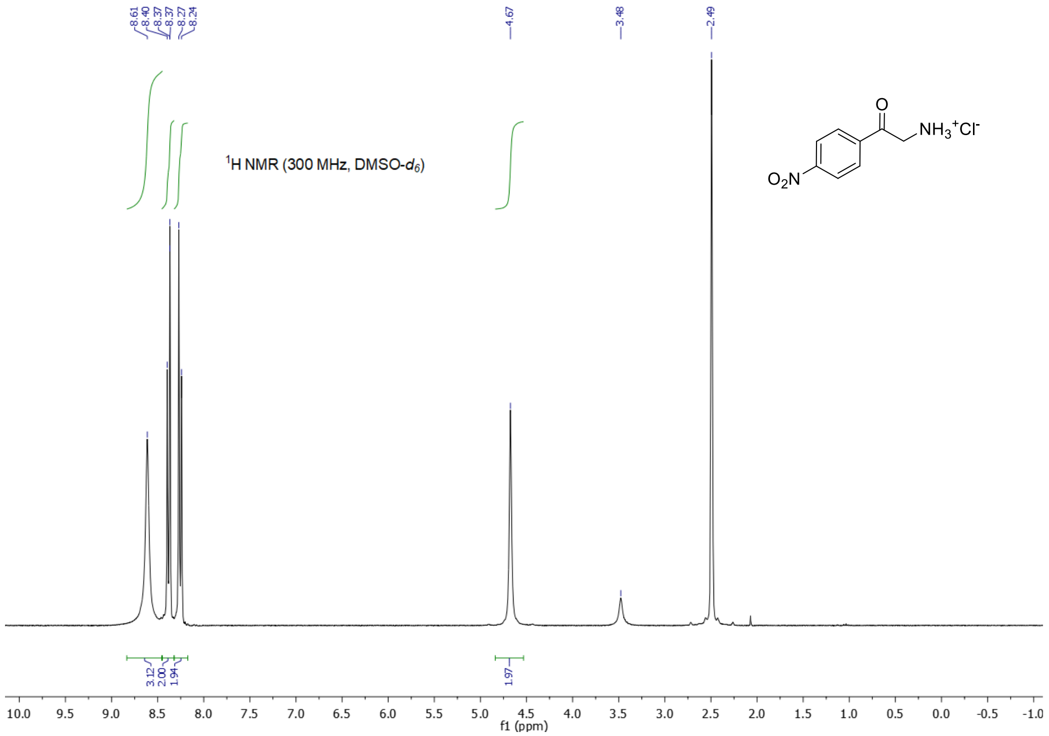

Figure S53. ^{1}H NMR spectrum of **10b**.

20. Ethyl 2-((2-(3-cyanophenyl)-2-oxoethyl)amino)-2-oxoacetate (11a)

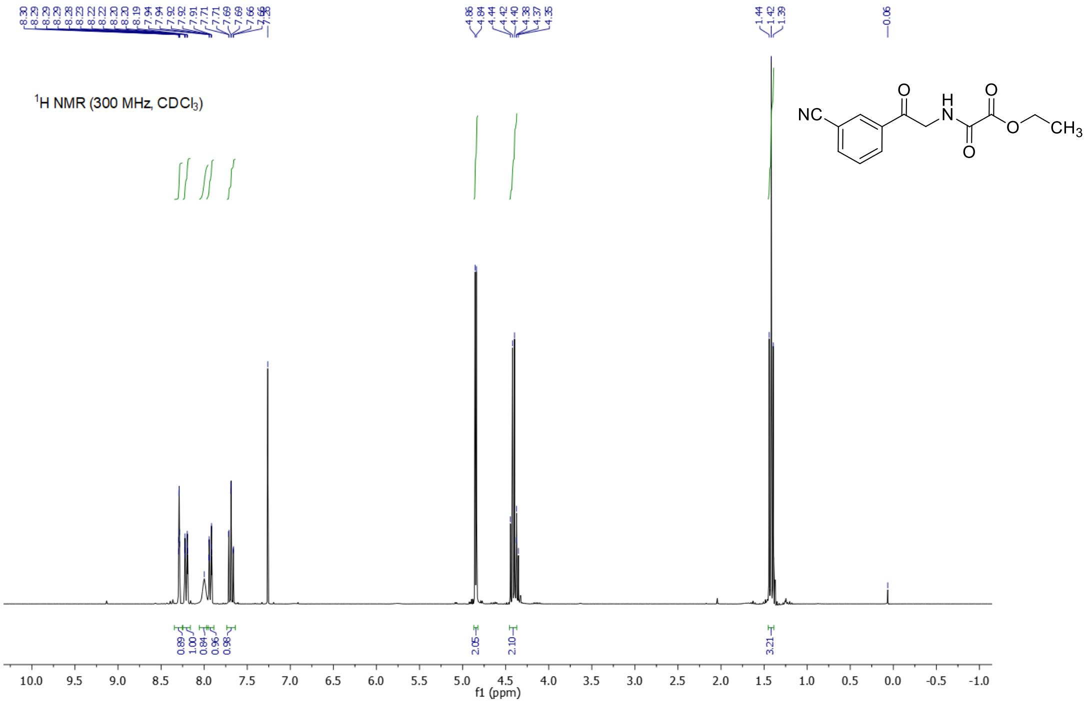

Figure S54. ^{1}H NMR spectrum of **11a**.

21. Ethyl 2-((2-(4-nitrophenyl)-2-oxoethyl)amino)-2-oxoacetate (11b)

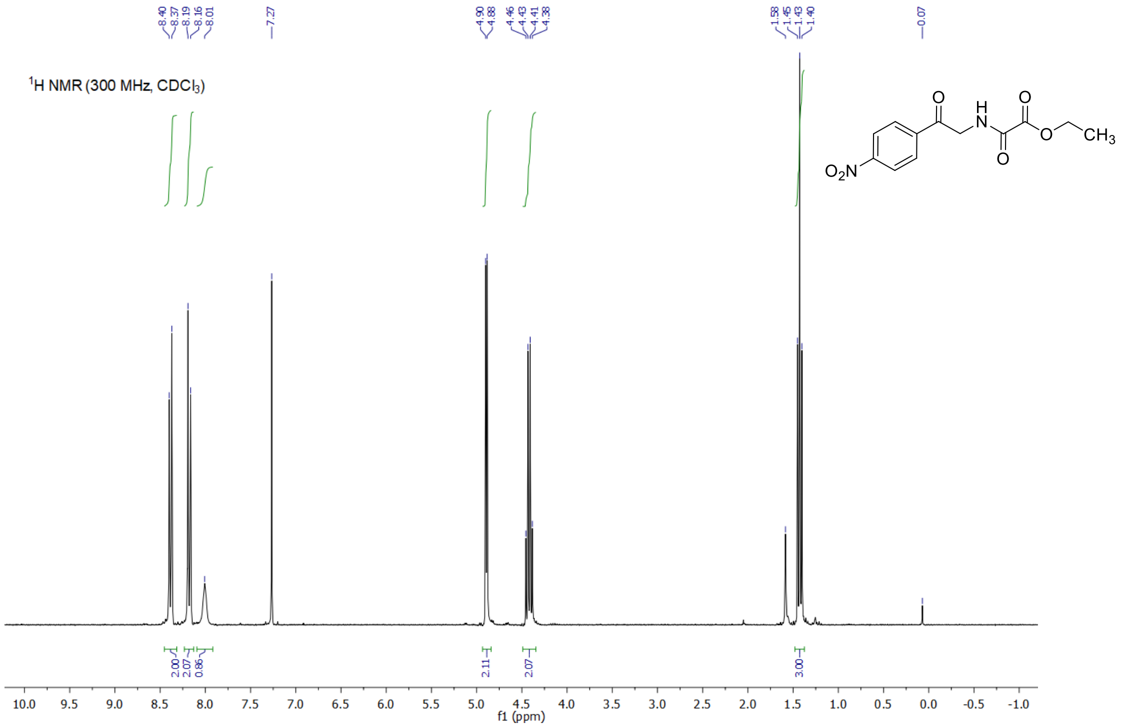

Figure S55. ¹H NMR spectrum of **11b**.

22. Ethyl 5-(3-cyanophenyl)thiazole-2-carboxylate (12a)

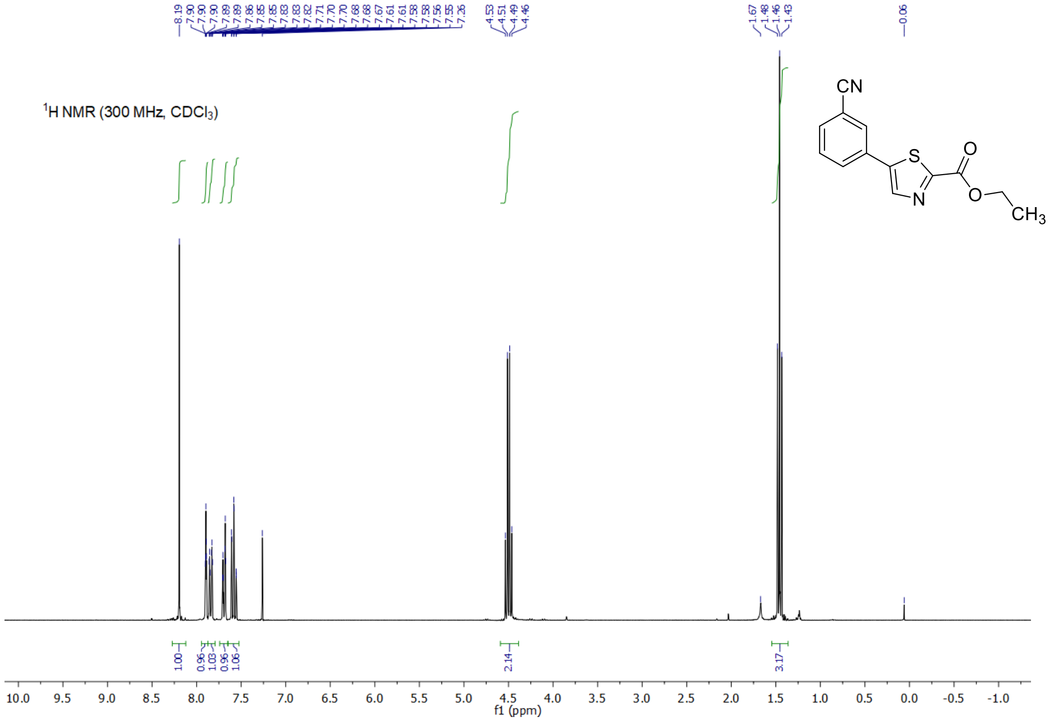

Figure S56. ¹H NMR spectrum of **12a**.

23. Ethyl 5-(4-nitrophenyl)thiazole-2-carboxylate (12b)

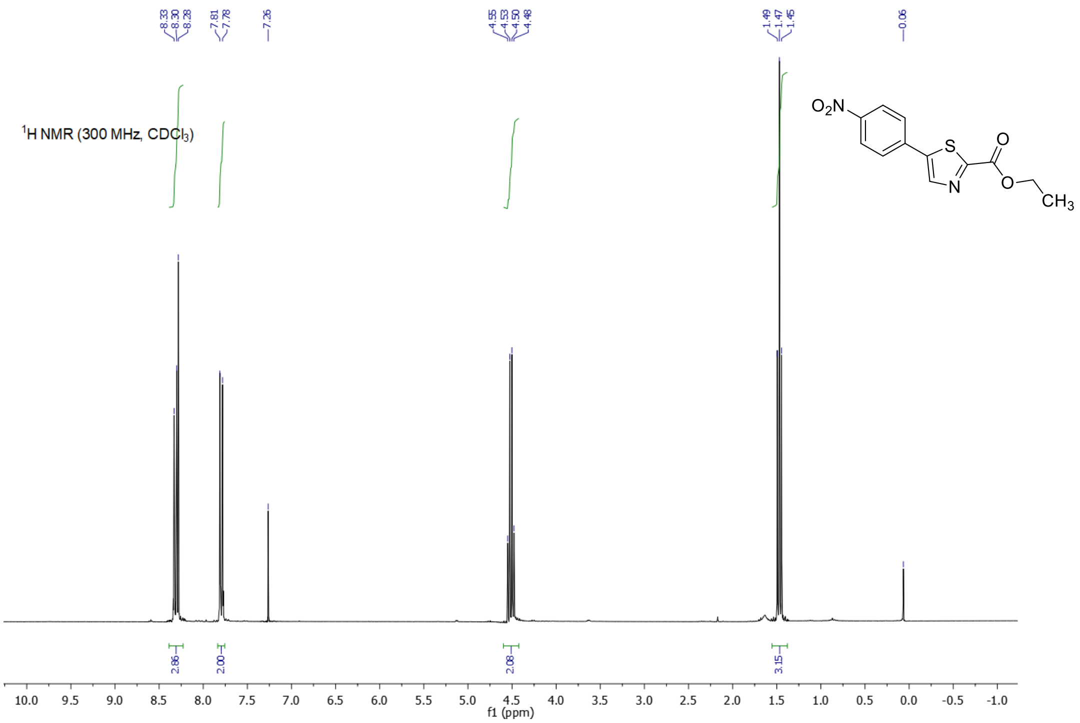

Figure S57. ^{1}H NMR spectrum of **12b**.

24. Ethyl 5-(3-cyanophenyl)oxazole-2-carboxylate (13a)

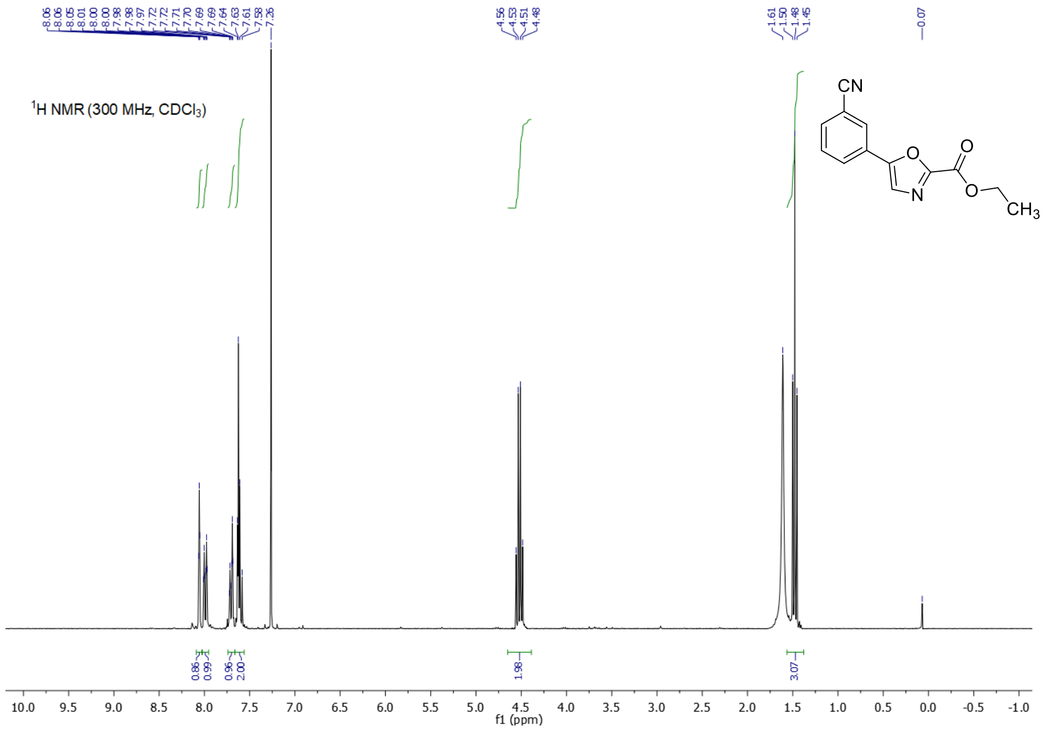

Figure S58. ^{1}H NMR spectrum of **13a**.

25. Ethyl 5-(4-nitrophenyl)oxazole-2-carboxylate (13b)

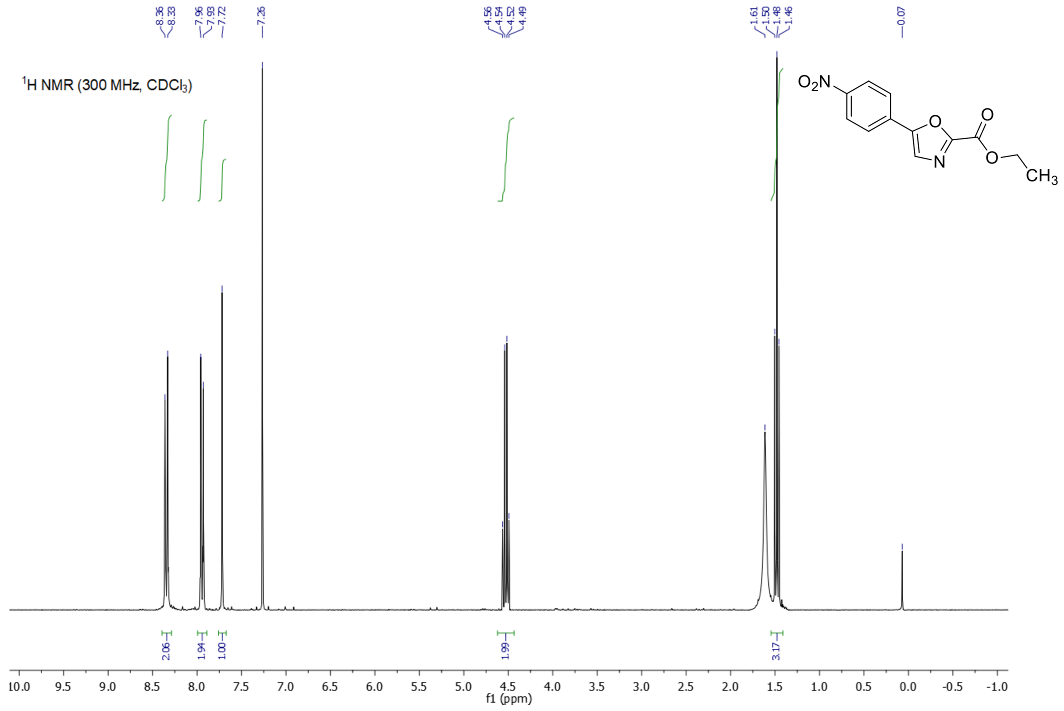

Figure S59. ^{1}H NMR spectrum of **13b**.

26. Ethyl 5-(3-cyanophenyl)-1*H*-imidazole-2-carboxylate (15a)

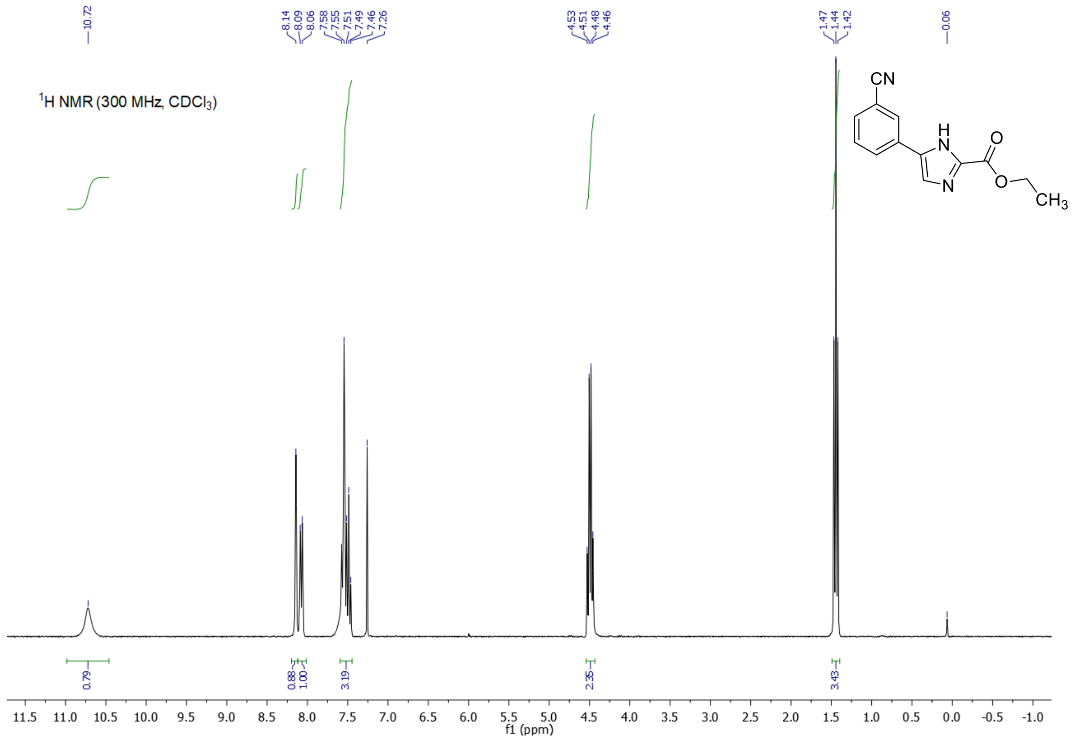

Figure S60. ^{1}H NMR spectrum of **15a**.

27. Ethyl 5-(4-nitrophenyl)-1*H*-imidazole-2-carboxylate (15b)

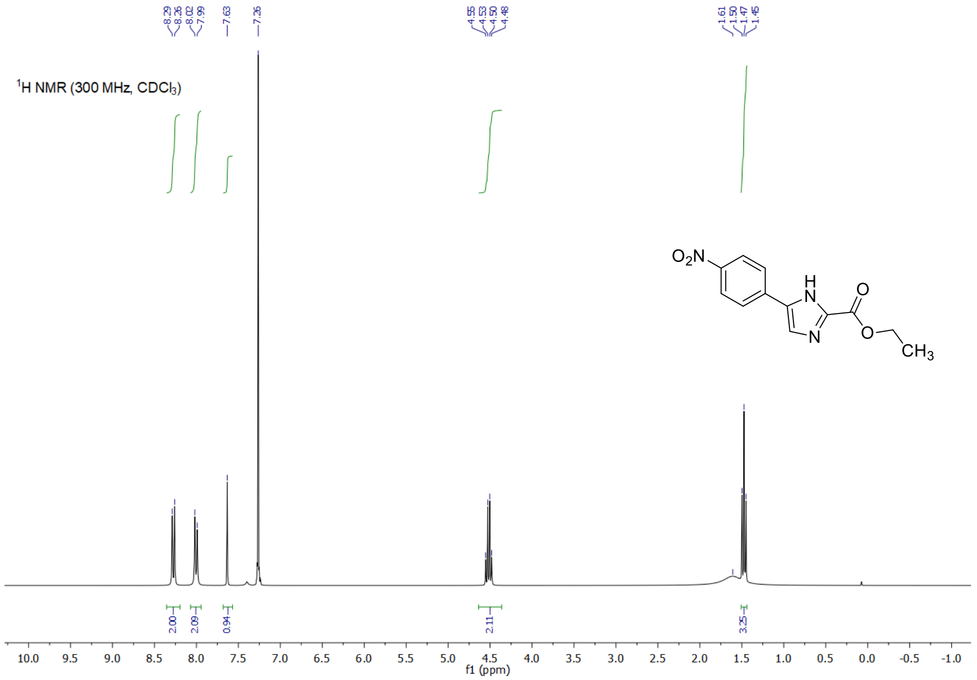

Figure S61. ^{1}H NMR spectrum of **15b**.

28. Methyl 3-cyanobenzoate (16a)

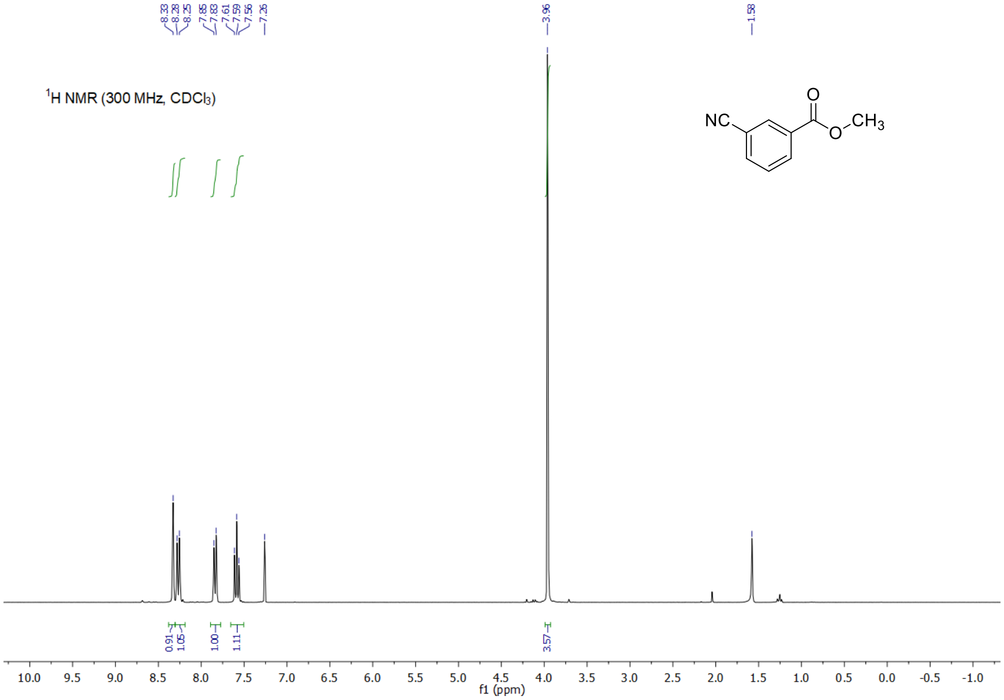

Figure S62. ^{1}H NMR spectrum of **16a**.

29. Ethyl 4-nitrobenzoate (16b)

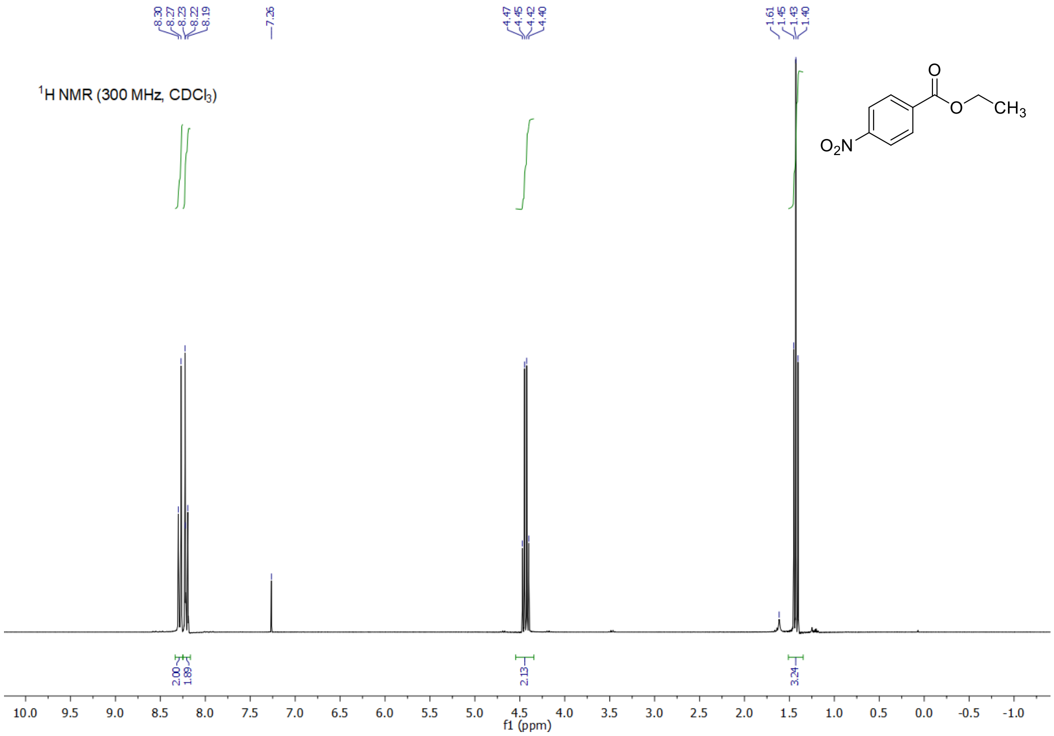

Figure S63. ^{1}H NMR spectrum of **16b**.

30. 3-Cyanobenzohydrazide (17a)

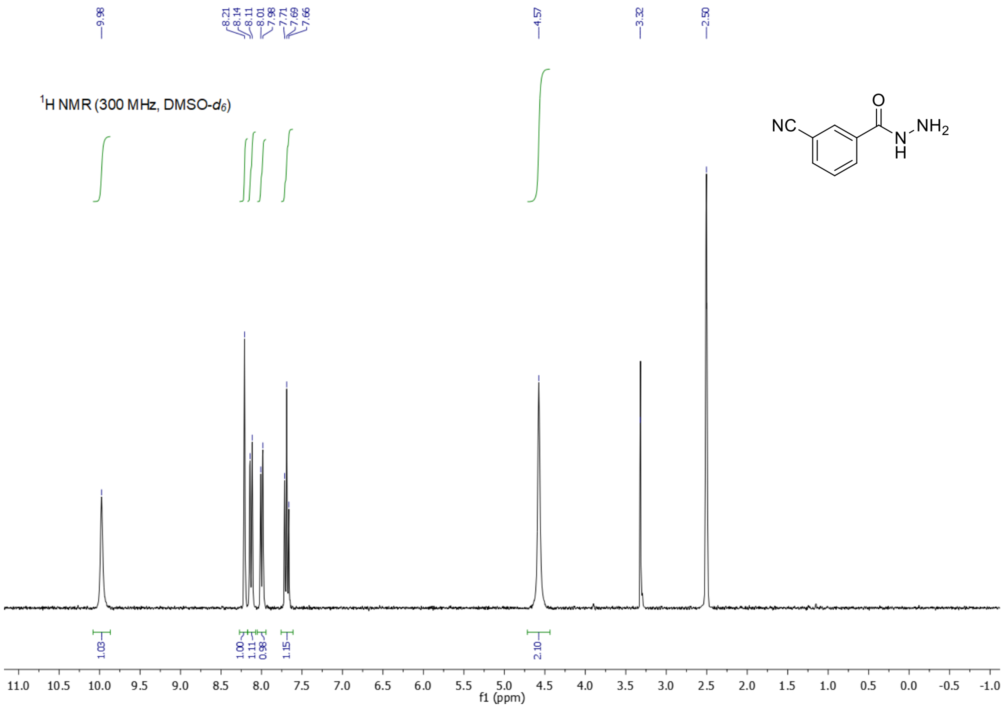

Figure S64. ^{1}H NMR spectrum of **17a**.

31. 4-Nitrobenzohydrazide (17b)

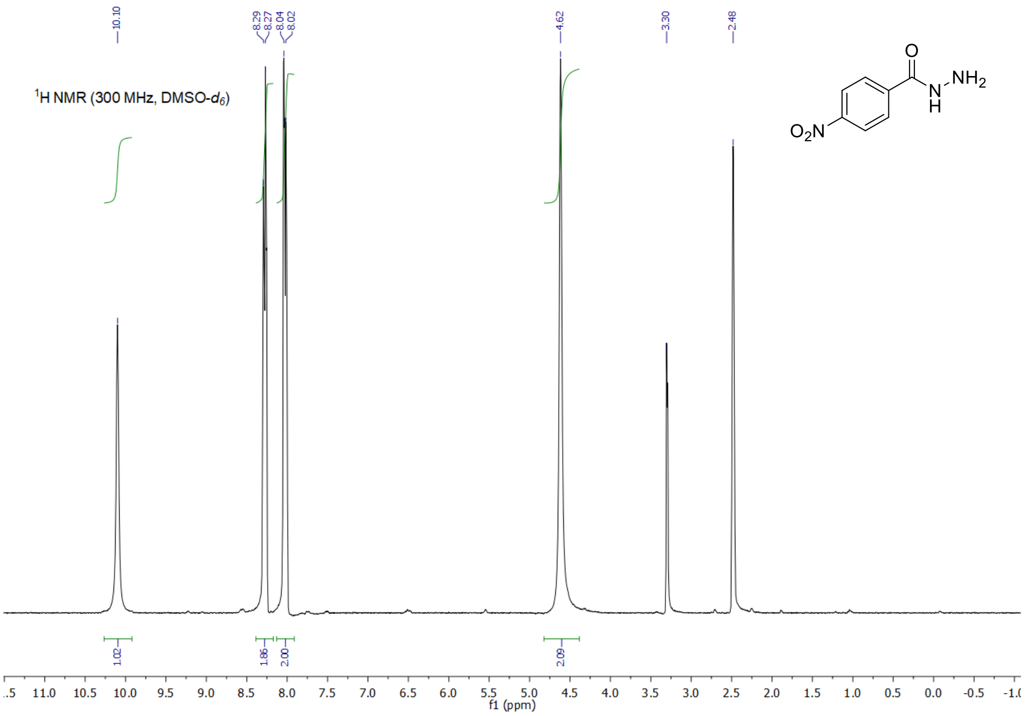

Figure S65. ^{1}H NMR spectrum of **17b**.

32. Ethyl 5-(3-cyanophenyl)-1,3,4-oxadiazole-2-carboxylate (19a)

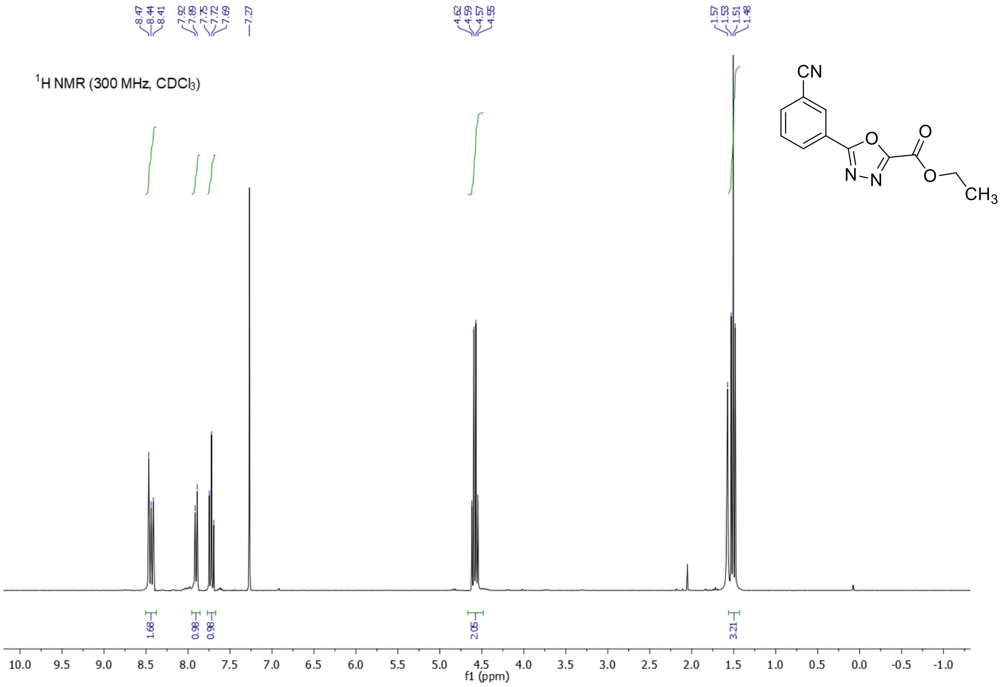

Figure S66. ^{1}H NMR spectrum of **19a**.

33. Ethyl 5-(4-nitrophenyl)-1,3,4-oxadiazole-2-carboxylate (19b)

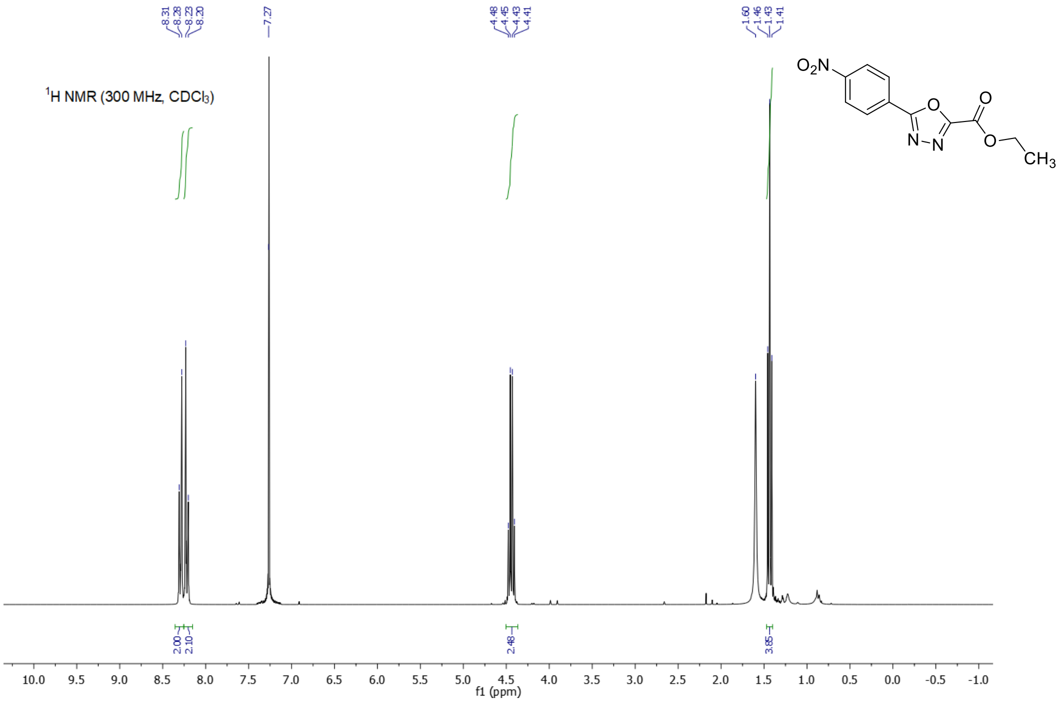

Figure S67. ^{1}H NMR spectrum of **19b**.

34. Ethyl 1-(3-cyanophenyl)-1*H*-1,2,3-triazole-4-carboxylate (20a)

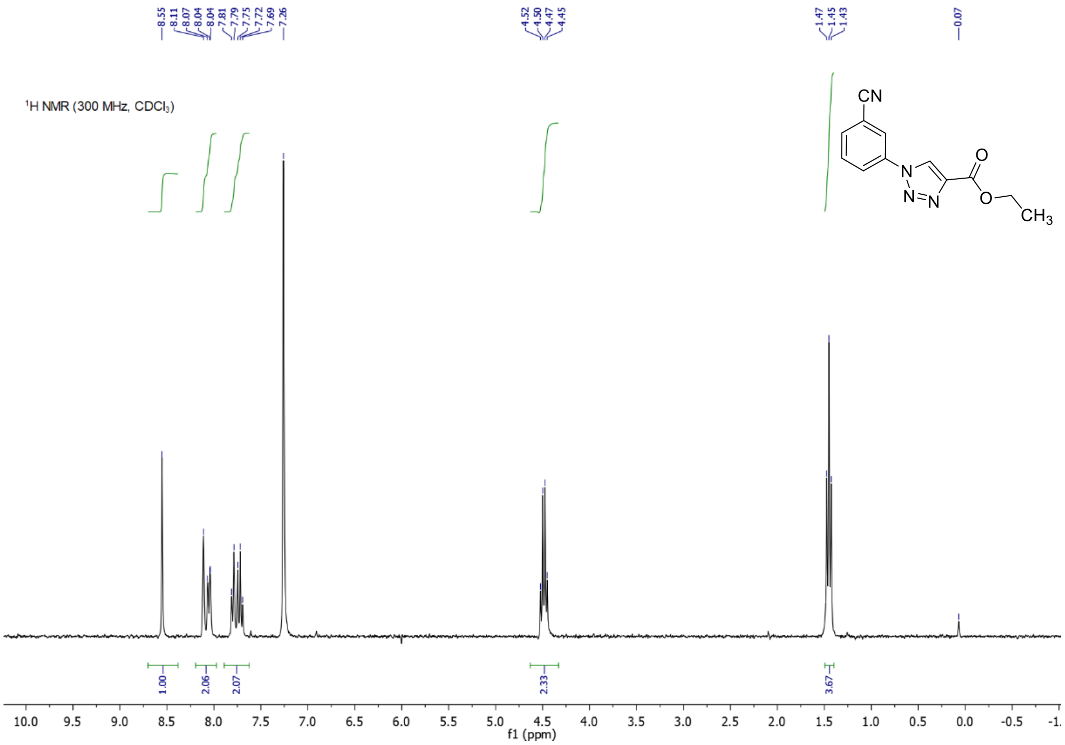

Figure S68. ^{1}H NMR spectrum of **20a**.

35. Ethyl 1-(4-nitrophenyl)-1*H*-1,2,3-triazole-4-carboxylate (20b)

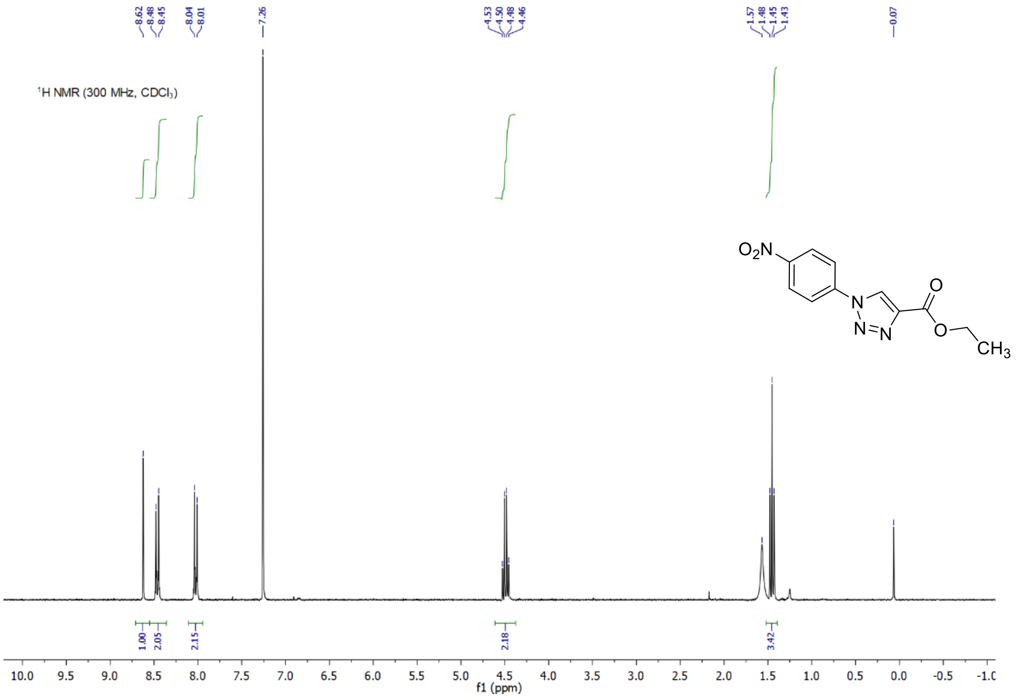

Figure S69. ^{1}H NMR spectrum of **20b**.

36. 5-(3-Cyano-5-(trifluoromethyl)phenyl)furan-2-carboxylic acid (IV)

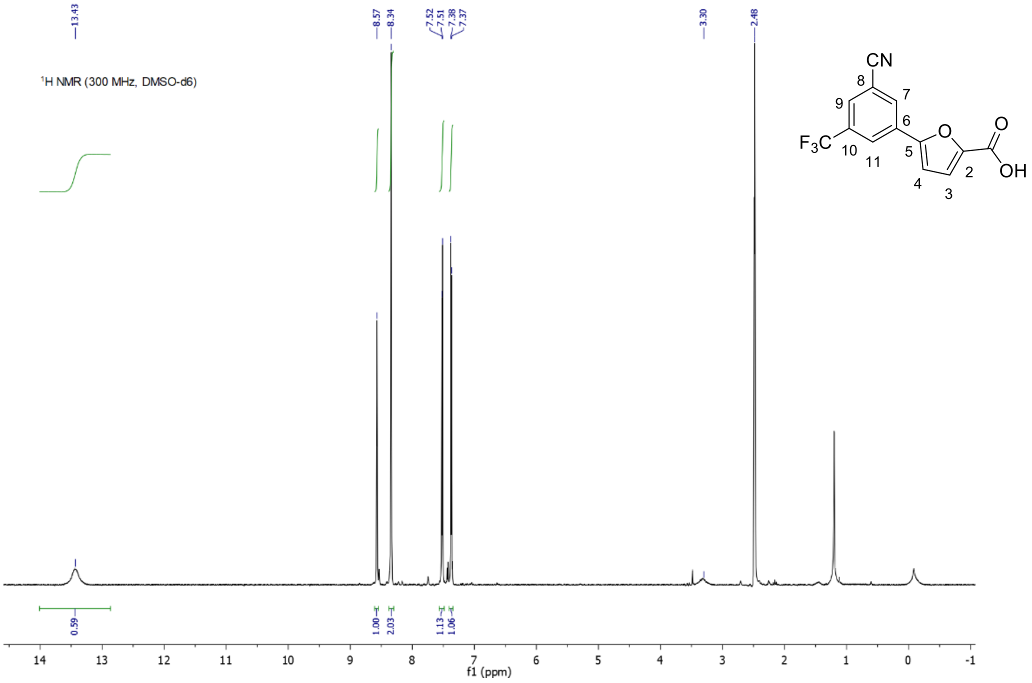

Figure S70. ^{1}H NMR spectrum of **IV**.

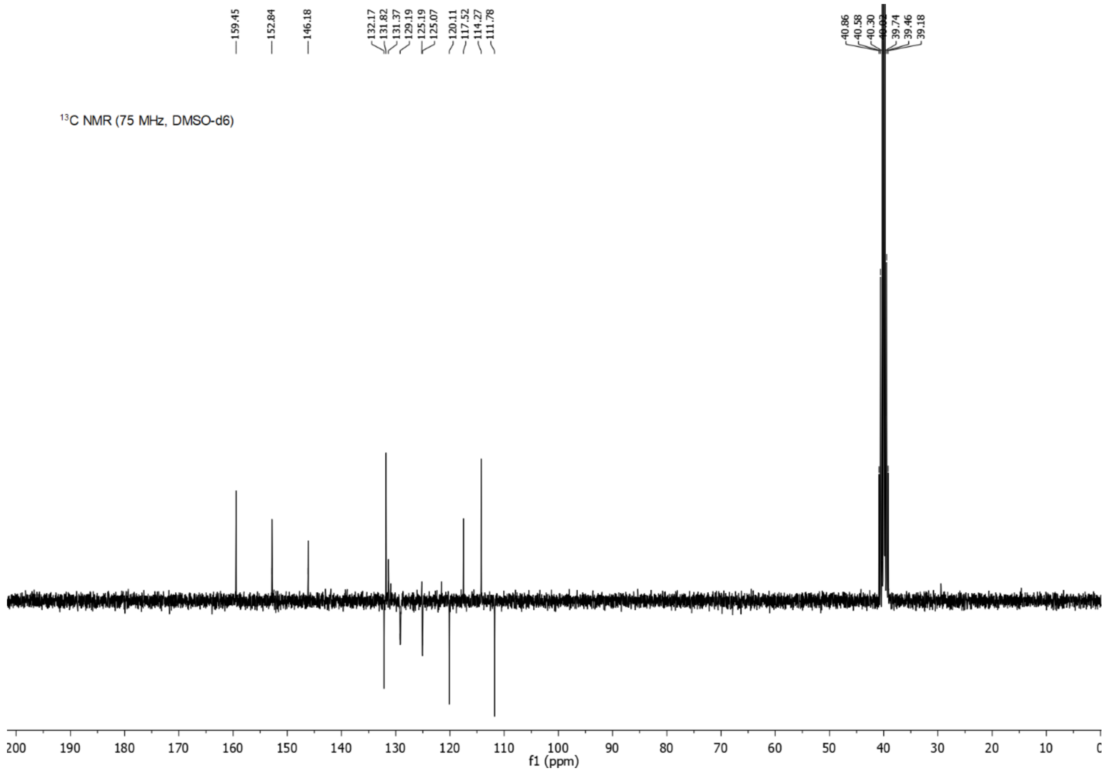

Figure S71. ^{13}C APT NMR spectrum of **IV**.

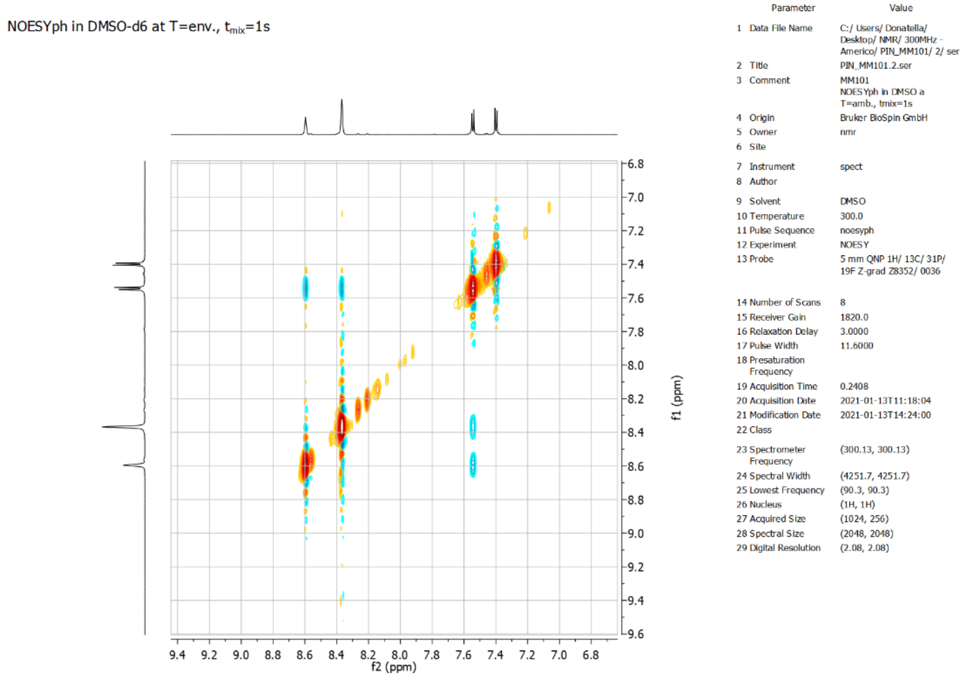

Figure S72. NOESY spectrum of **IV**.

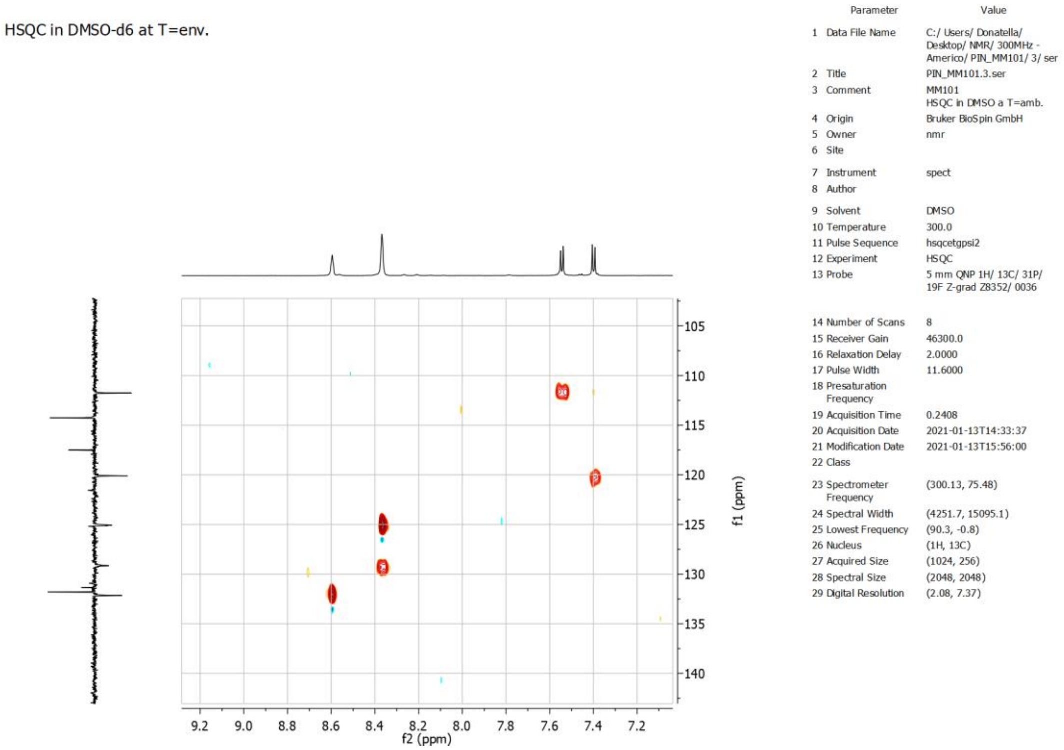

Figure S73. HSQC spectrum of **IV**.

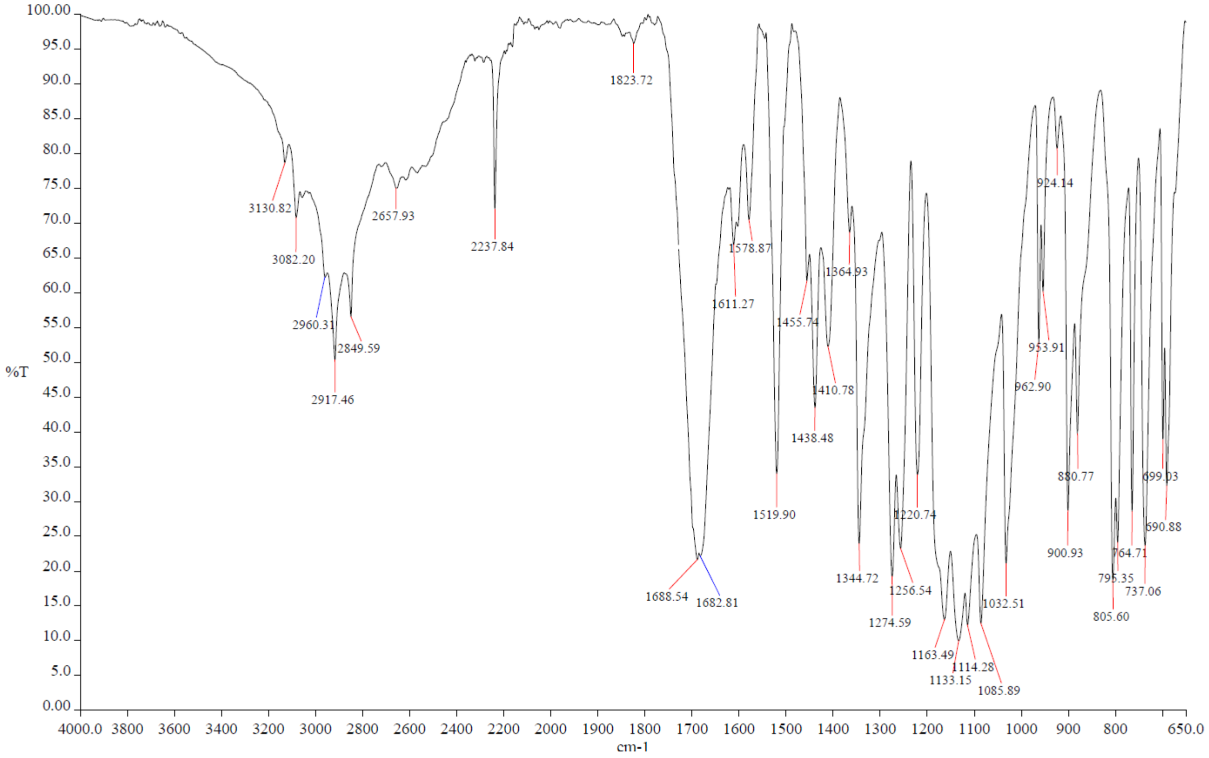

Figure S74. FT-IR spectrum of **IV**.

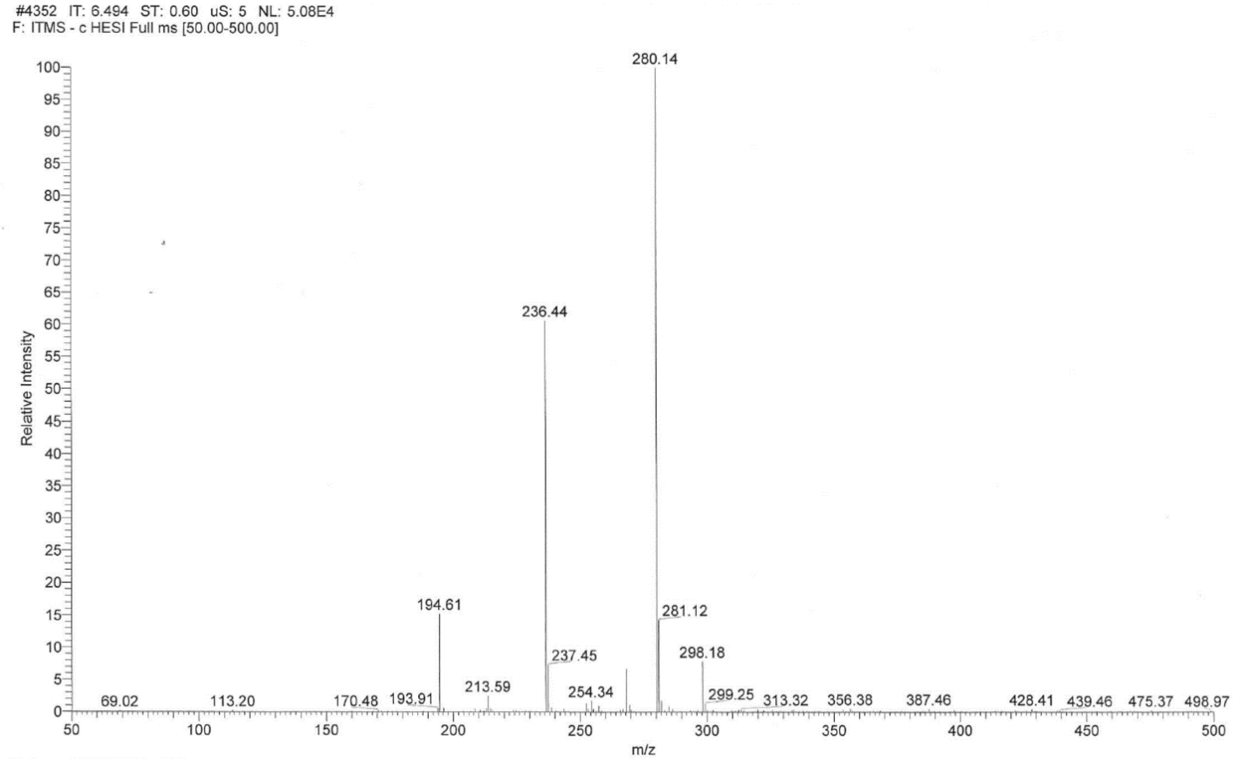

Figure S75. ESI-MS spectrum of **IV**.

37. Methyl 5-(3-cyano-5-(trifluoromethyl)phenyl)furan-2-carboxylate (IV-E)

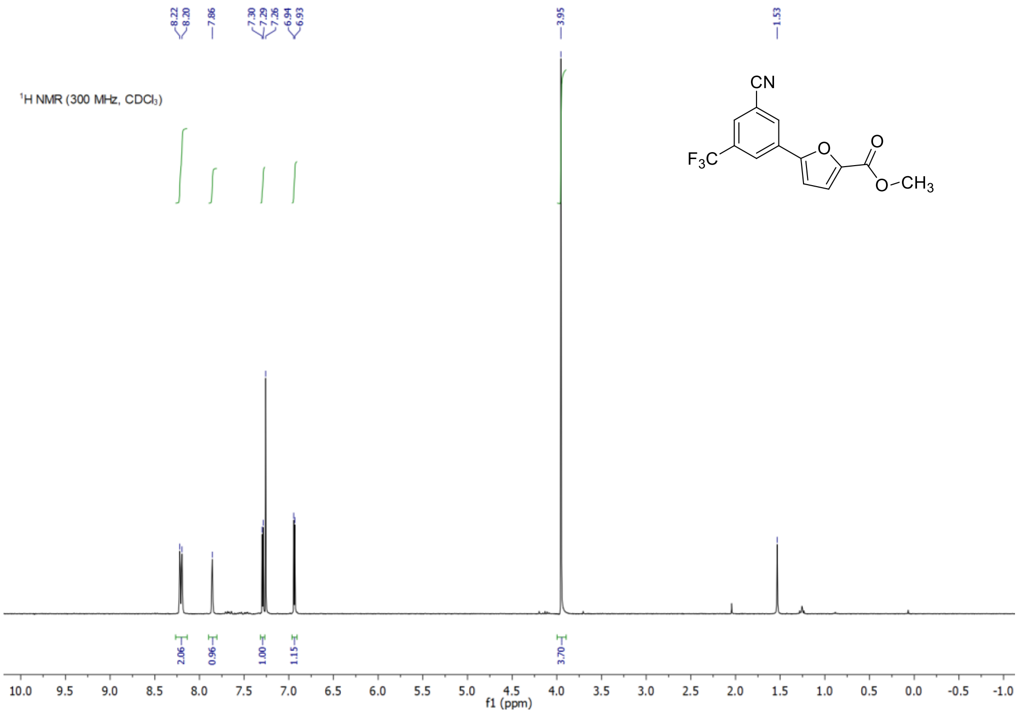

Figure S76. ^{1}H NMR spectrum of **IV-E**.

38. **5-(3,5-Bis(trifluoromethyl)phenyl)furan-2-carboxylic acid (V)**

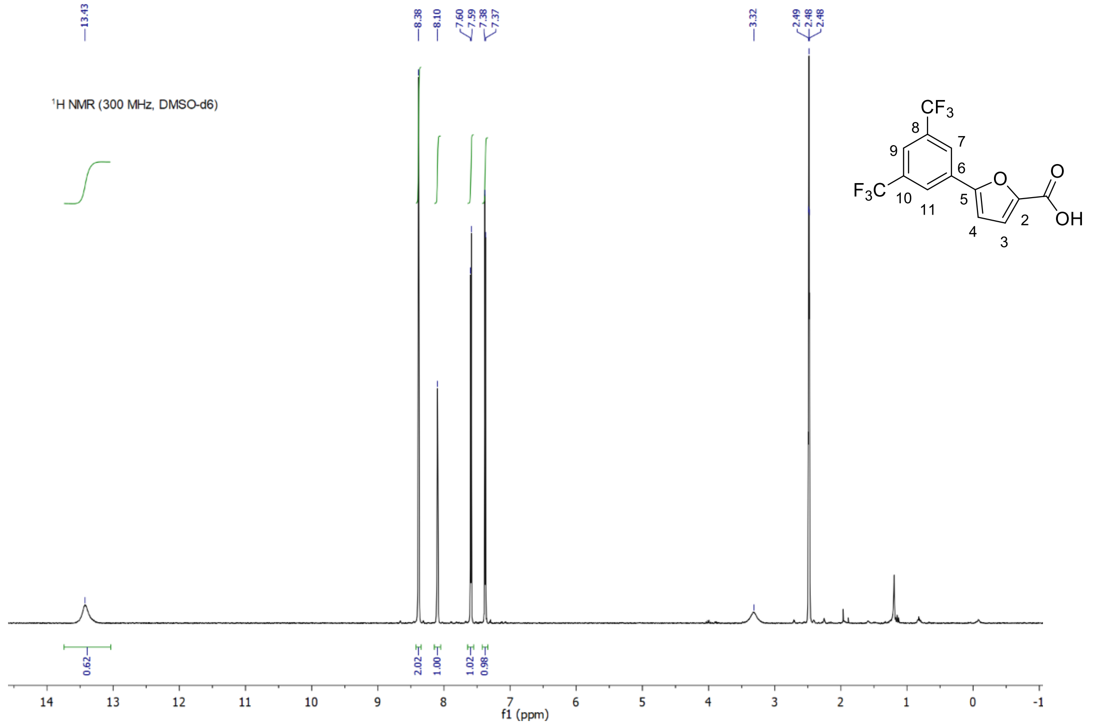

Figure S77. ^{1}H NMR spectrum of **V**.

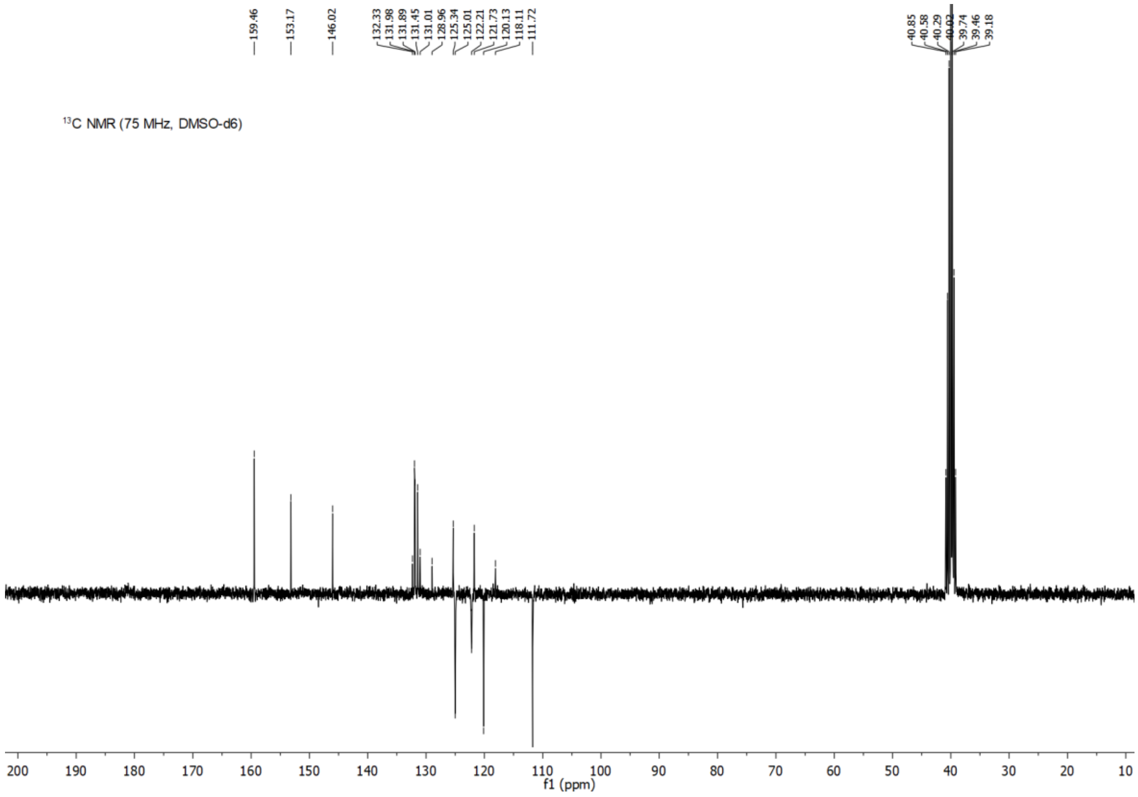

Figure S78. ^{13}C APT NMR spectrum of **V**.

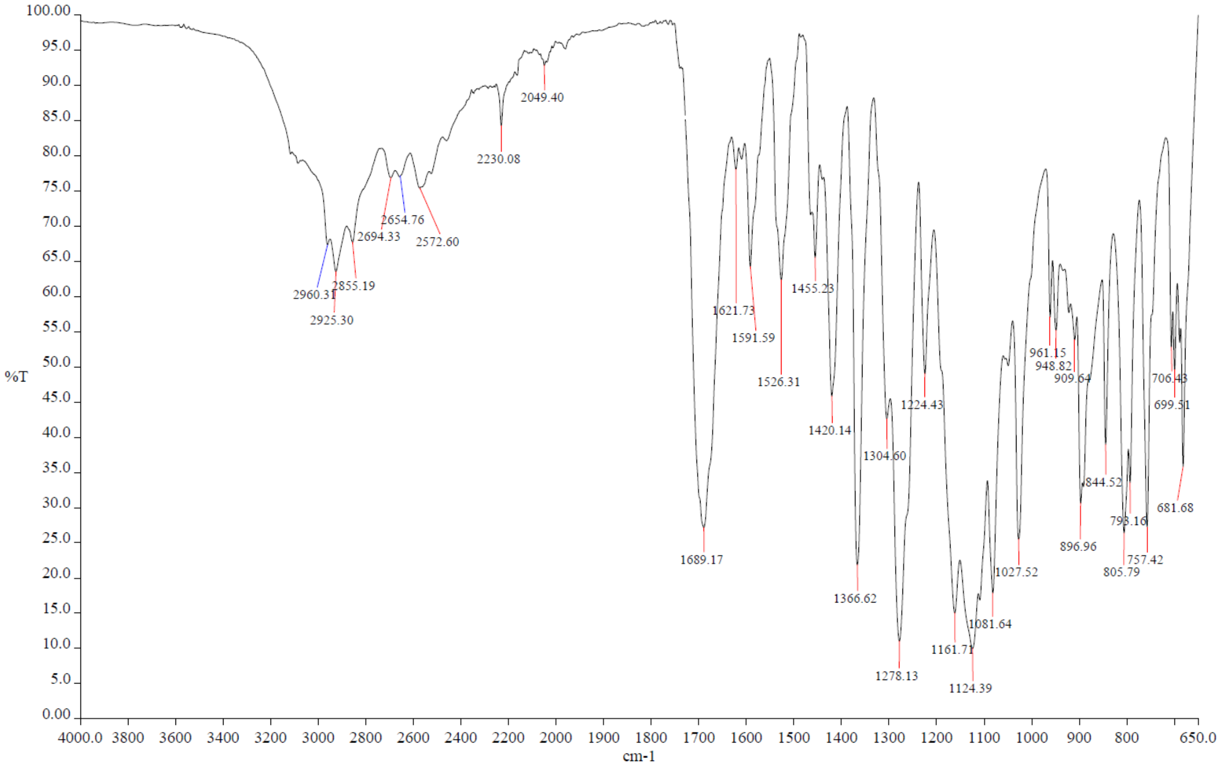

Figure S79. FT-IR spectrum of **V**.

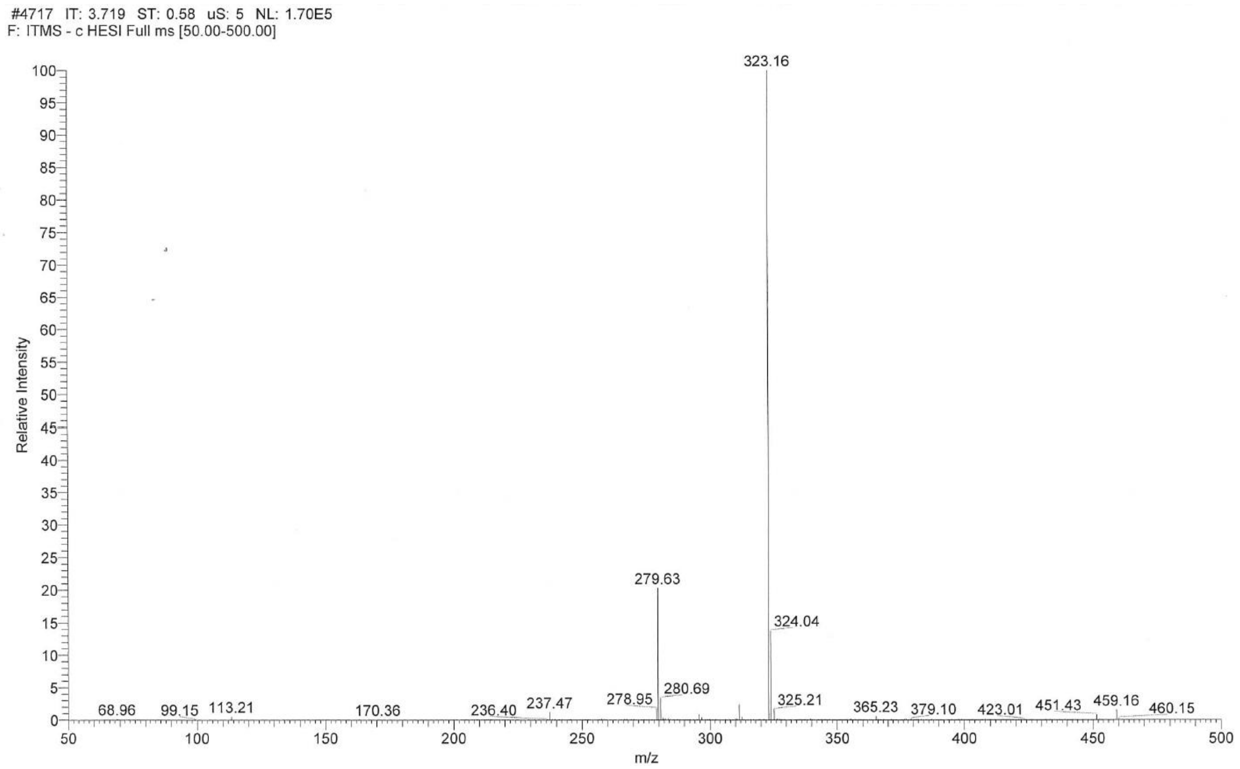

Figure S80. ESI-MS spectrum of **V**.

39. **Methyl 5-(3,5-bis(trifluoromethyl)phenyl)furan-2-carboxylate (V-E)**

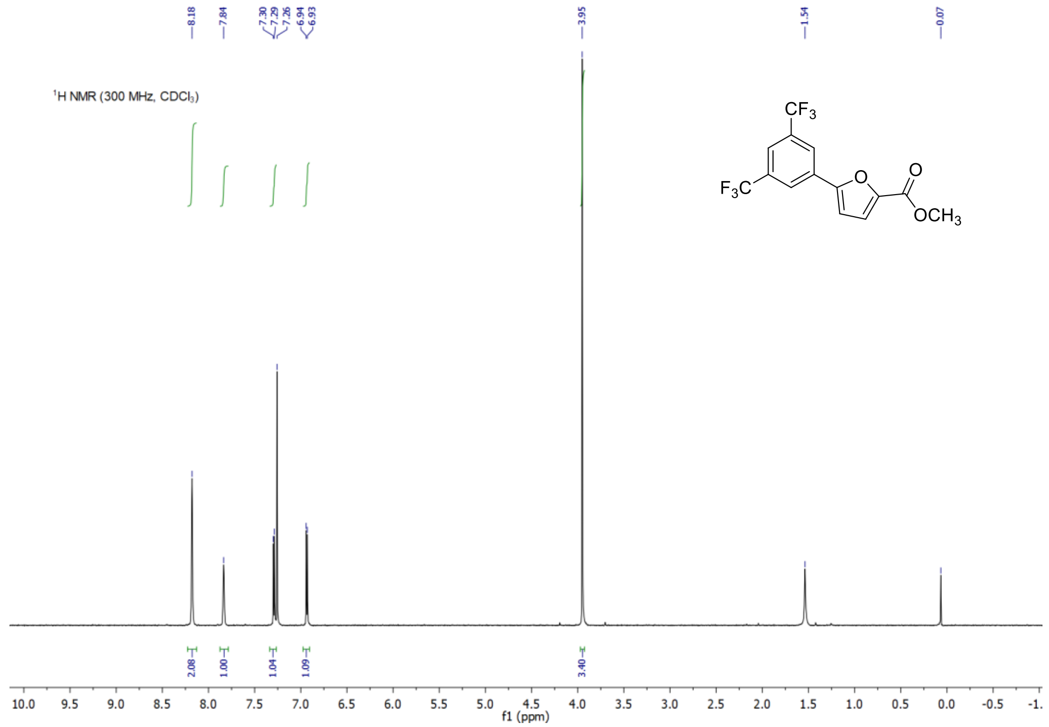

Figure S81. ^{1}H NMR spectrum of **V-E**.

40. **5-(3-Cyano-5-fluorophenyl)furan-2-carboxylic acid (VI)**

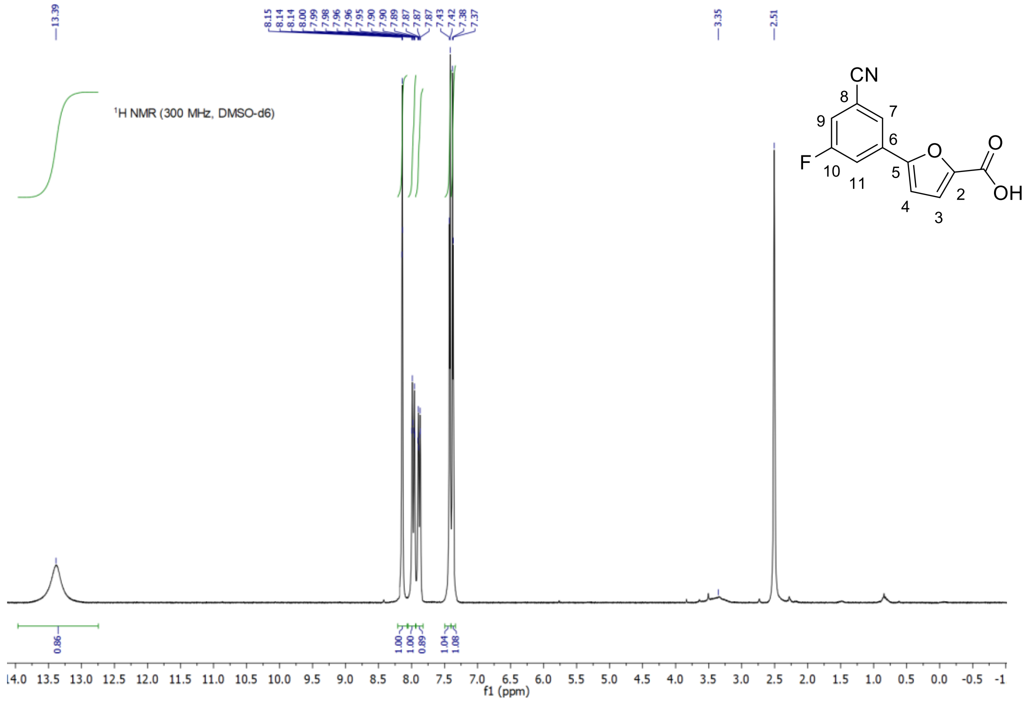

Figure S82. ^{1}H NMR spectrum of **VI**.

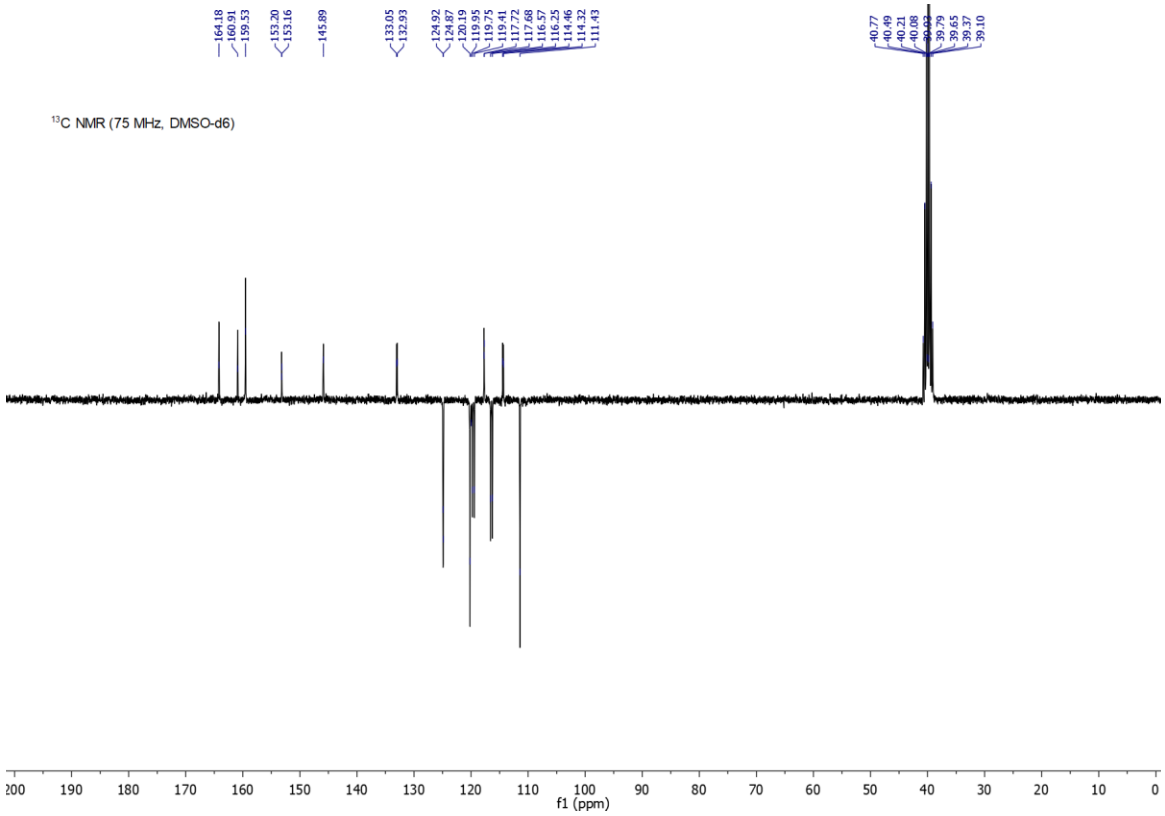

Figure S83. ^{13}C APT NMR spectrum of **VI**.

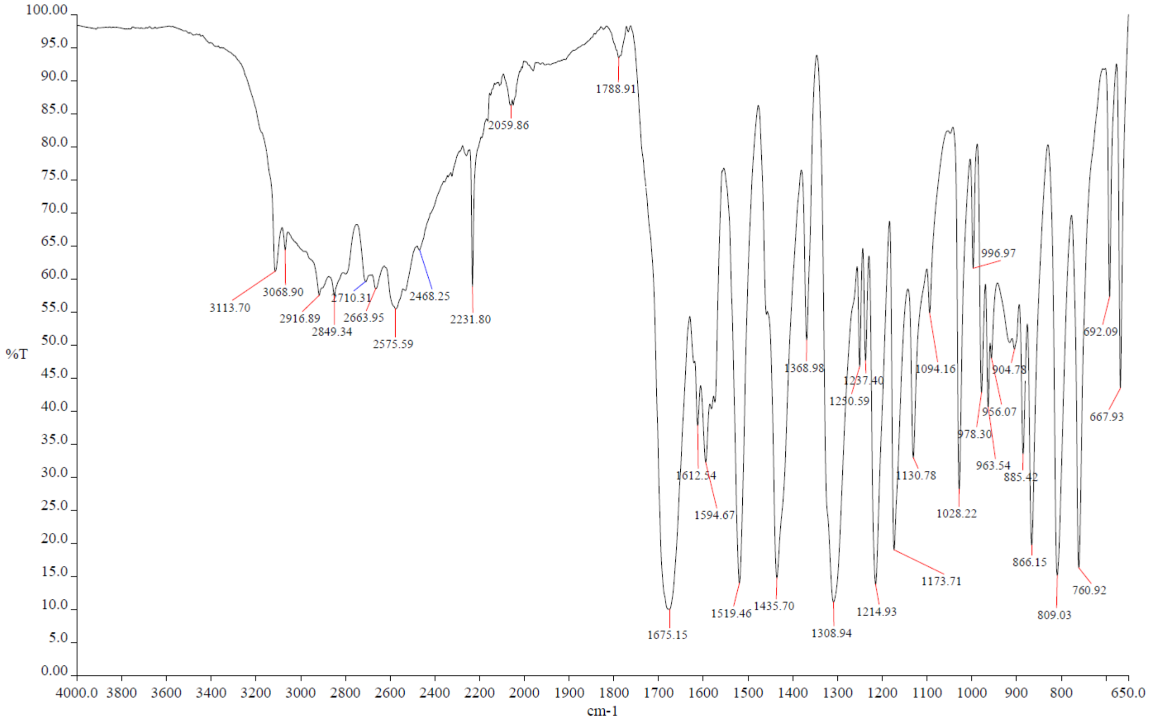

Figure S84. FT-IR spectrum of **VI**.

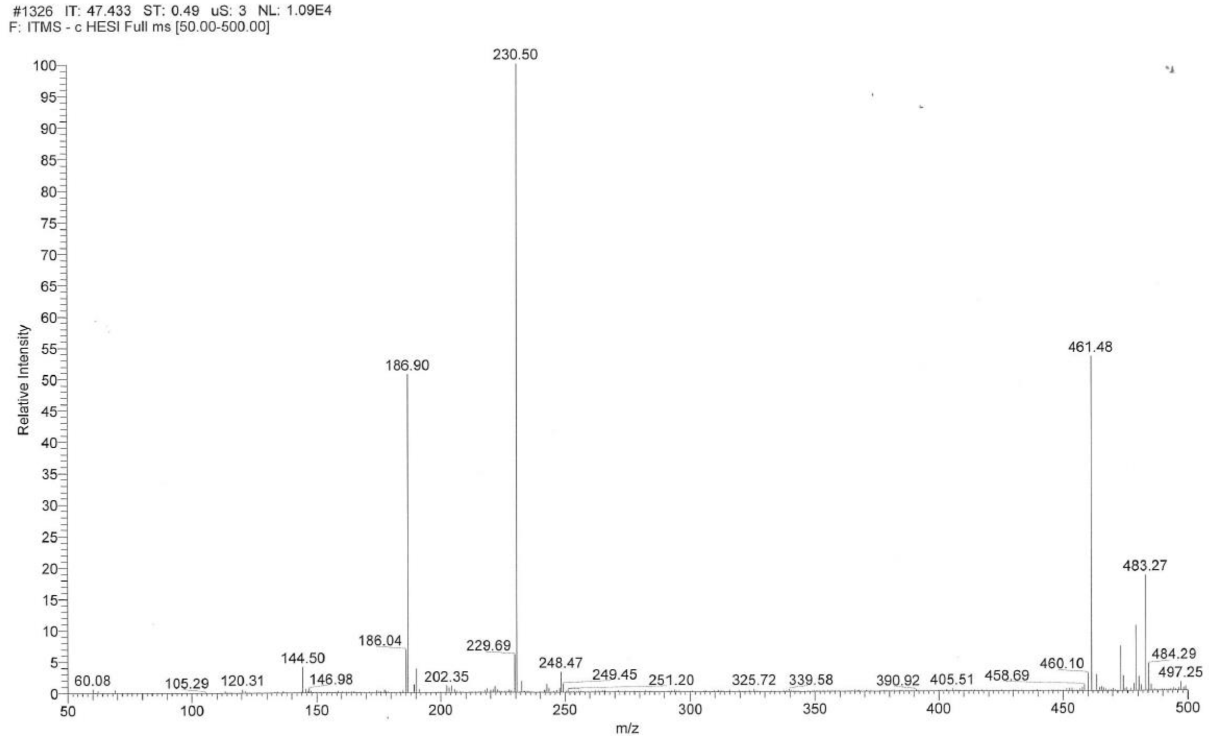

Figure S85. ESI-MS spectrum of **VI**.

41. Methyl 5-(3-cyano-5-fluorophenyl)furan-2-carboxylate (VI-E)

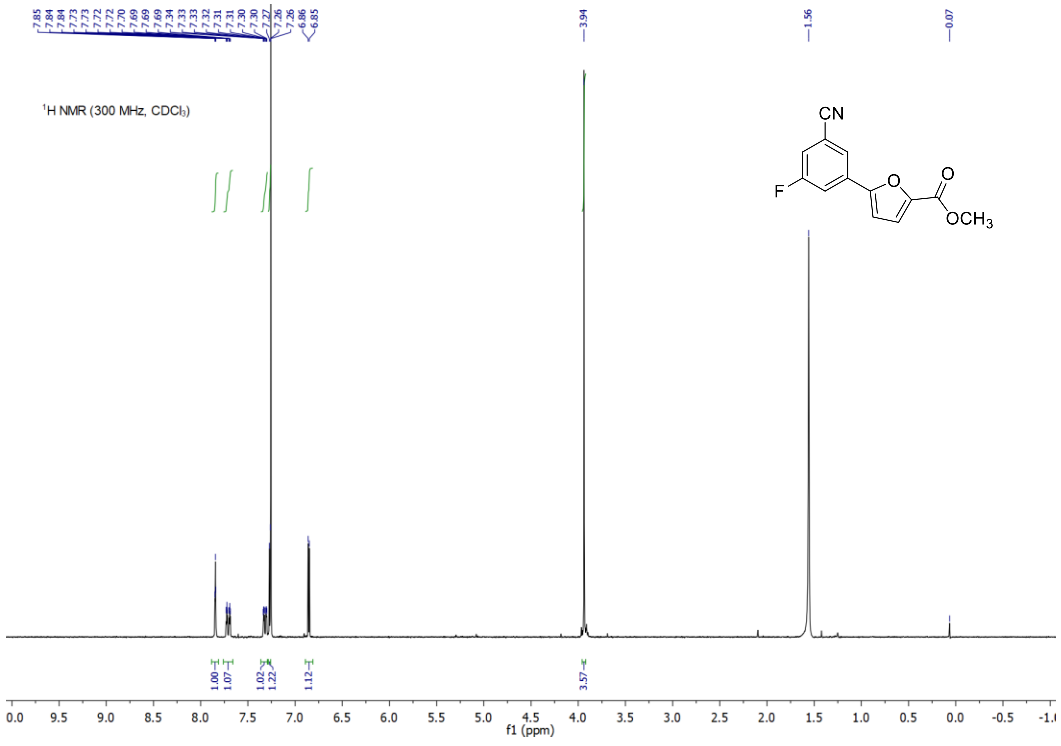

Figure S86. ^{1}H NMR spectrum of **VI-E**.

42. 5-(3-Cyano-5-methoxyphenyl)furan-2-carboxylic acid (VII)

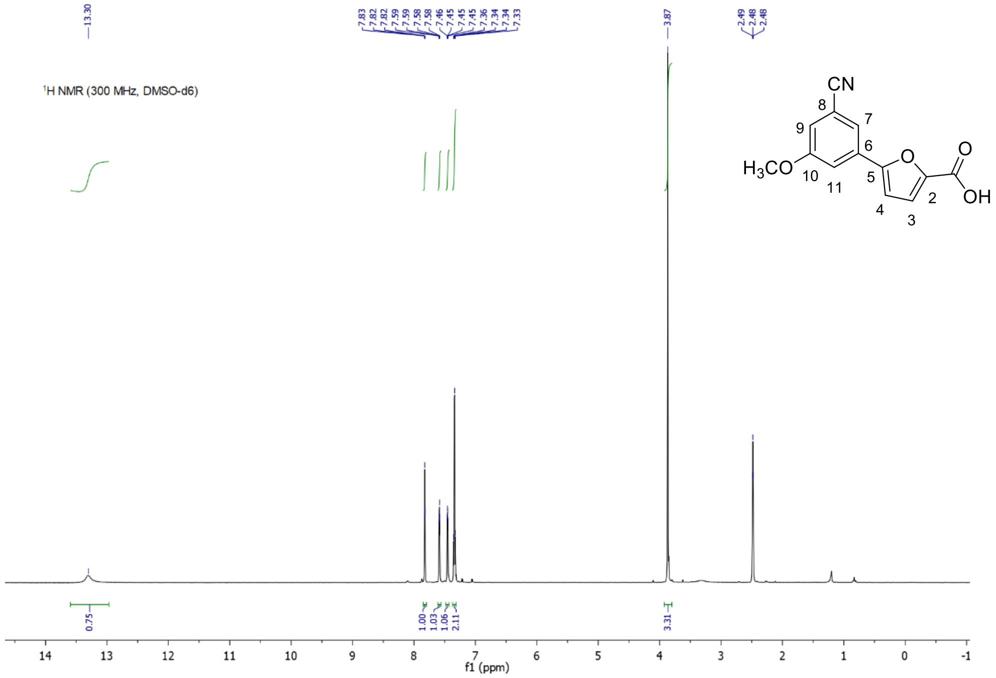

Figure S87. ^{1}H NMR spectrum of **VII**.

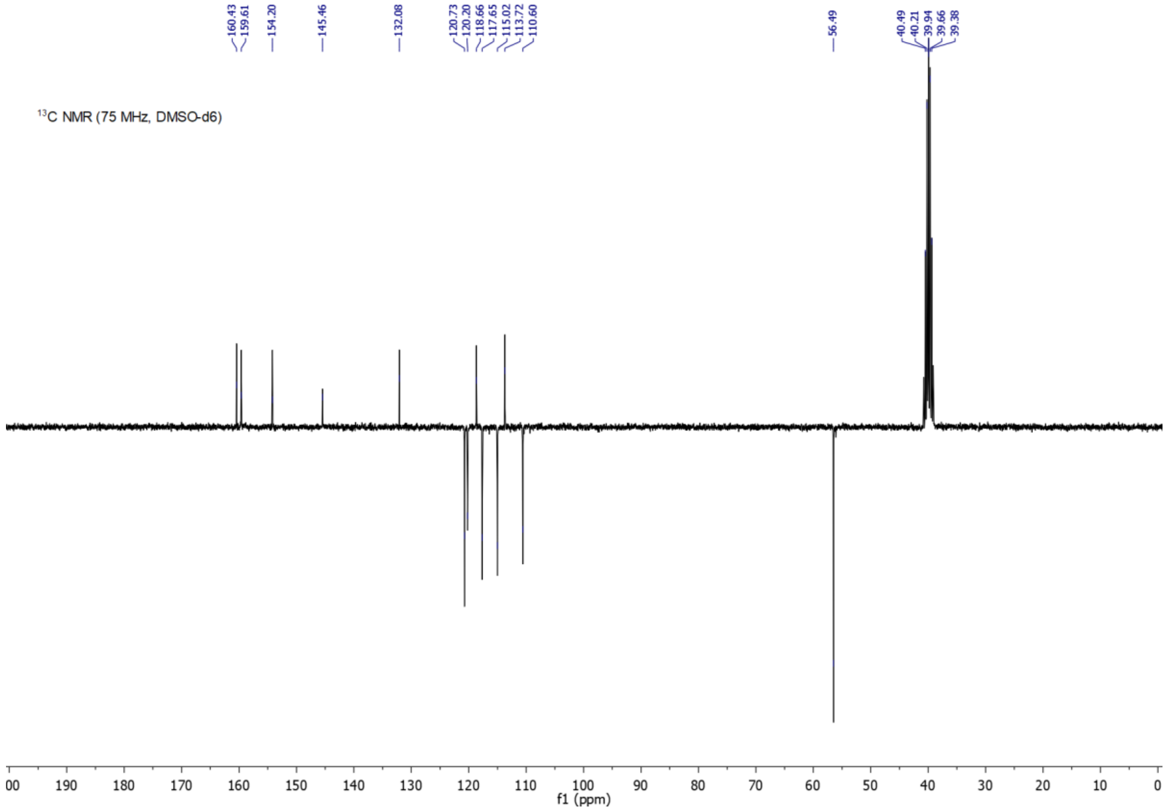

Figure S88. ^{13}C APT NMR spectrum of **VII**.

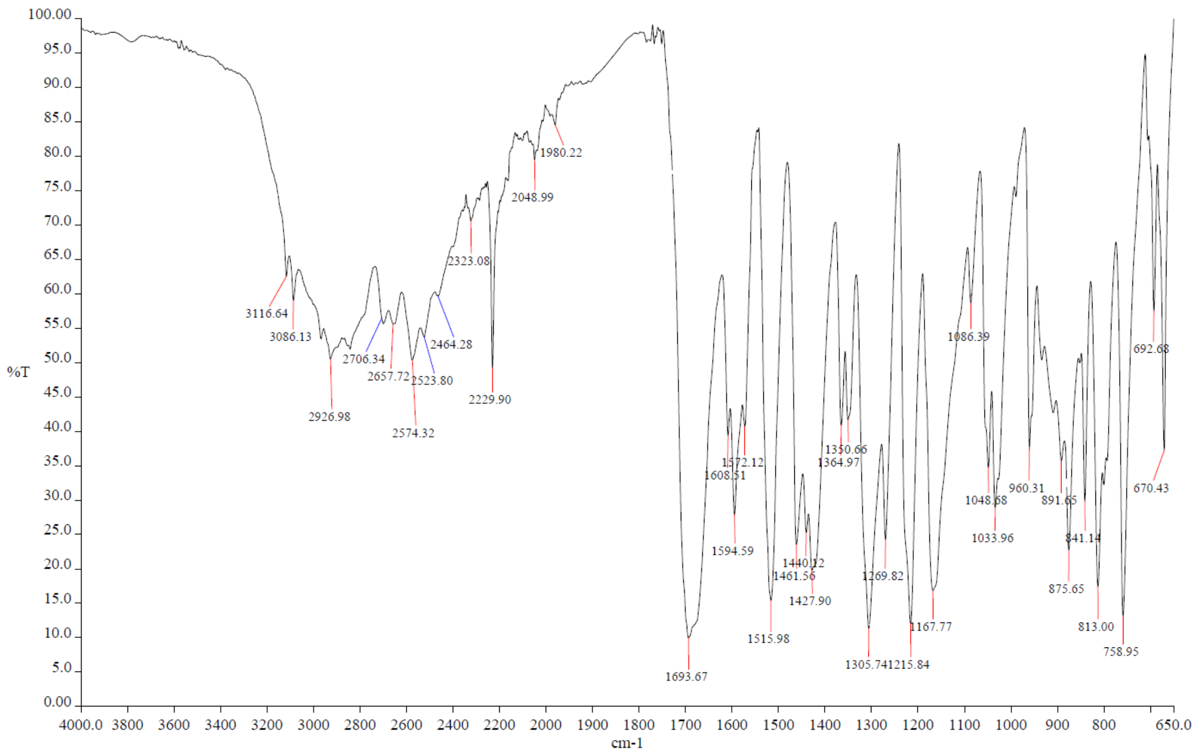

Figure S89. FT-IR spectrum of **VII**.

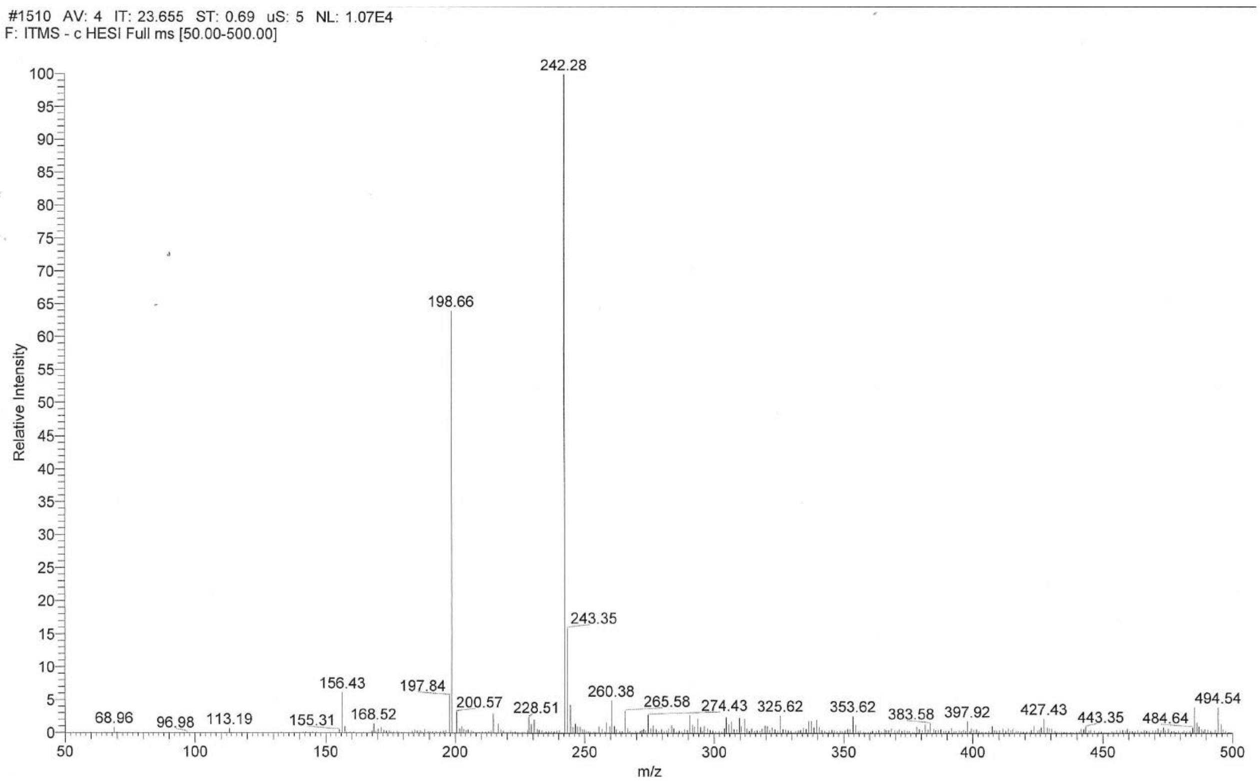

Figure S90. ESI-MS spectrum of **VII**.

43. Methyl 5-(3-cyano-5-methoxyphenyl)furan-2-carboxylate (VII-E)

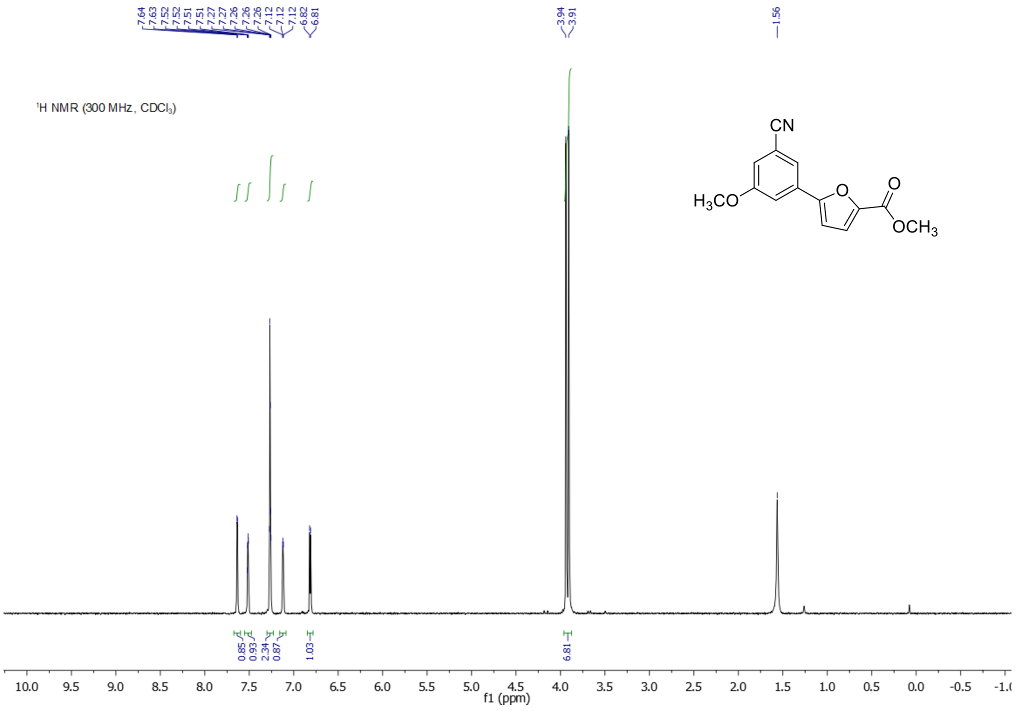

Figure S91. ^{1}H NMR spectrum of **VII-E**.

44. 5-(3-Cyano-5-methylphenyl)furan-2-carboxylic acid (VIII)

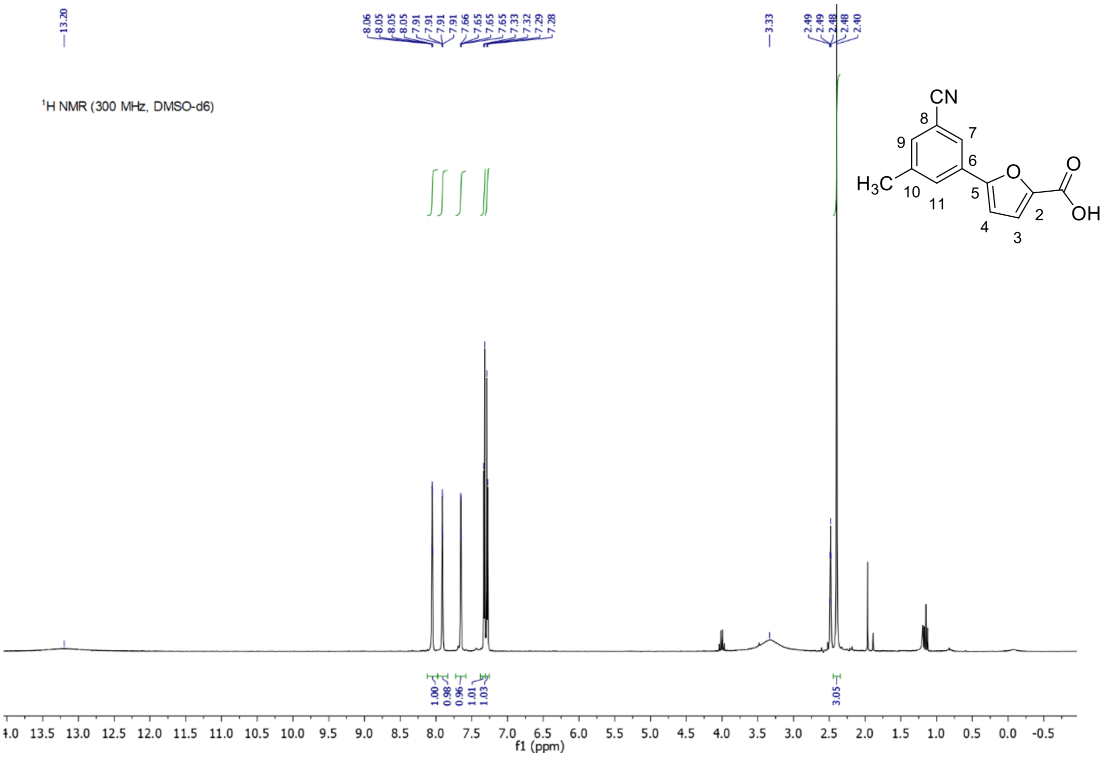

Figure S92. ^{1}H NMR spectrum of **VIII**.

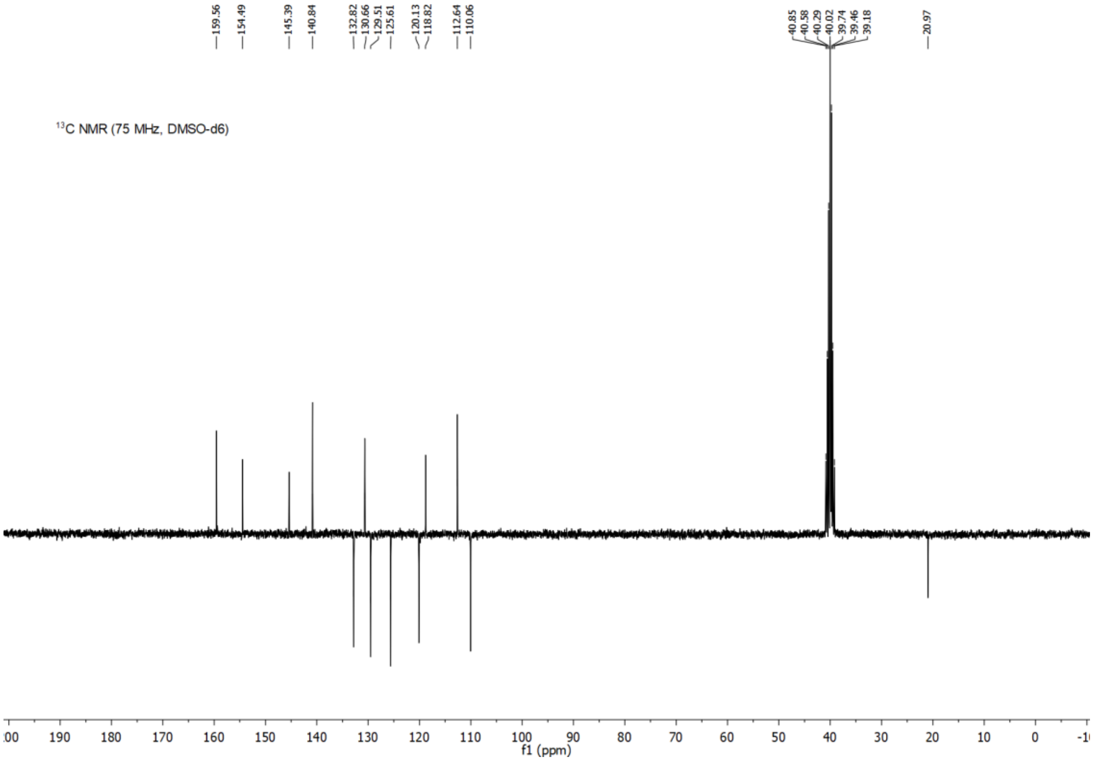

Figure S93. ^{13}C APT NMR spectrum of **VIII**.

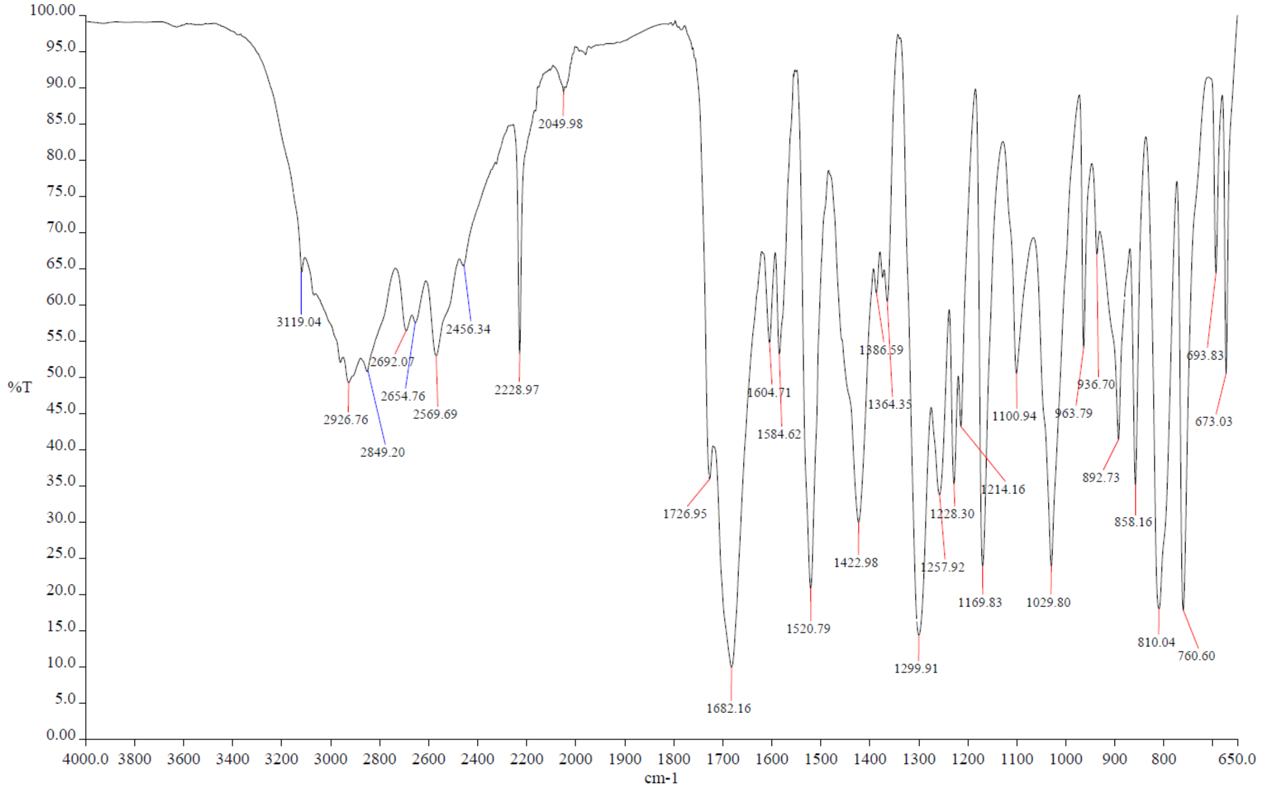

Figure S94. FT-IR spectrum of **VIII**.

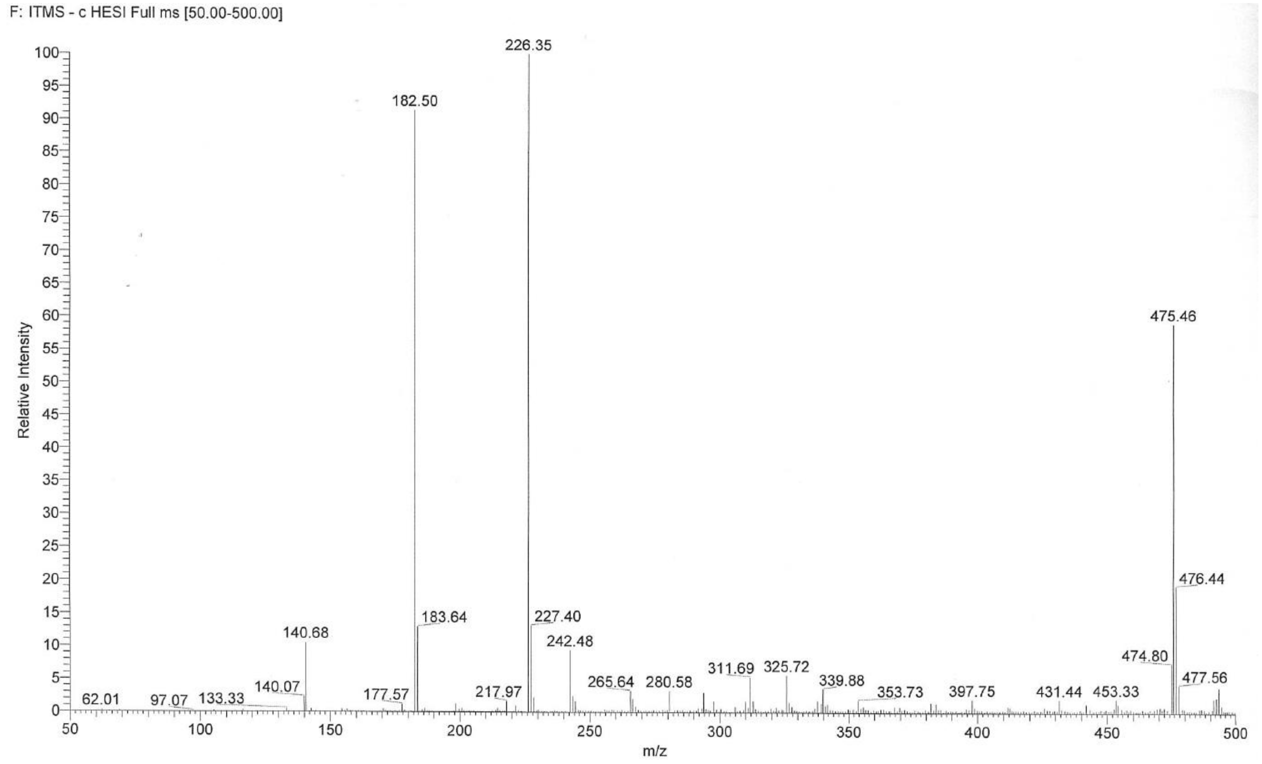

Figure S95. ESI-MS spectrum of **VIII**.

45. Methyl 5-(3-cyano-5-methylphenyl)furan-2-carboxylate (VIII-E)

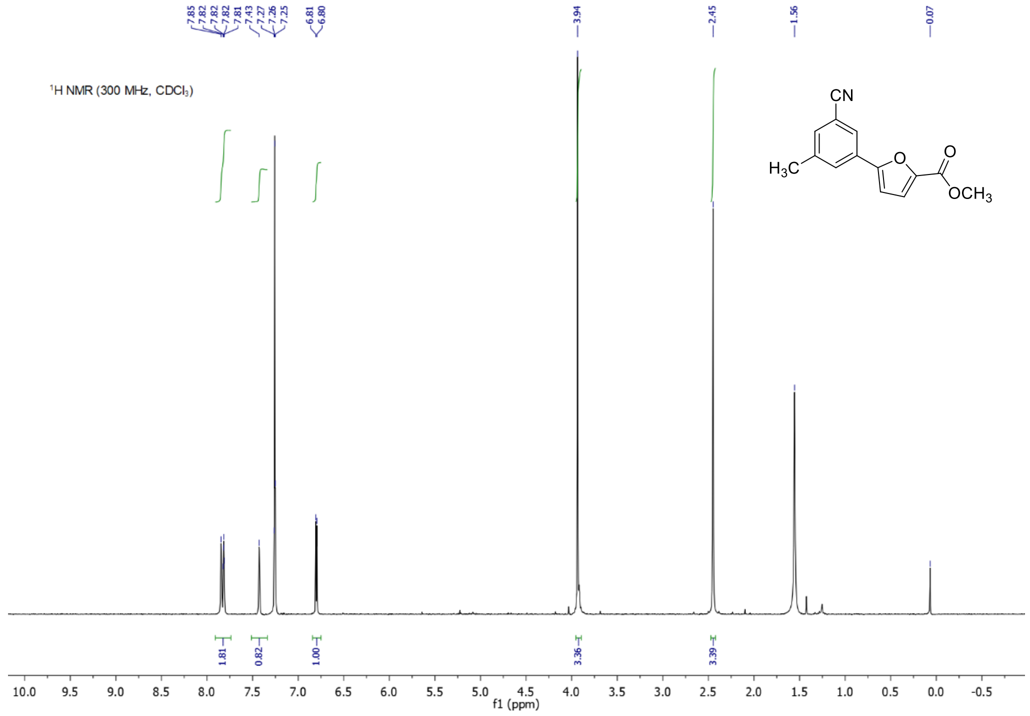

Figure S96. ^{1}H NMR spectrum of **VIII-E**.

46. 5-(3-Cyano-5-hydroxyphenyl)furan-2-carboxylic acid (IX)

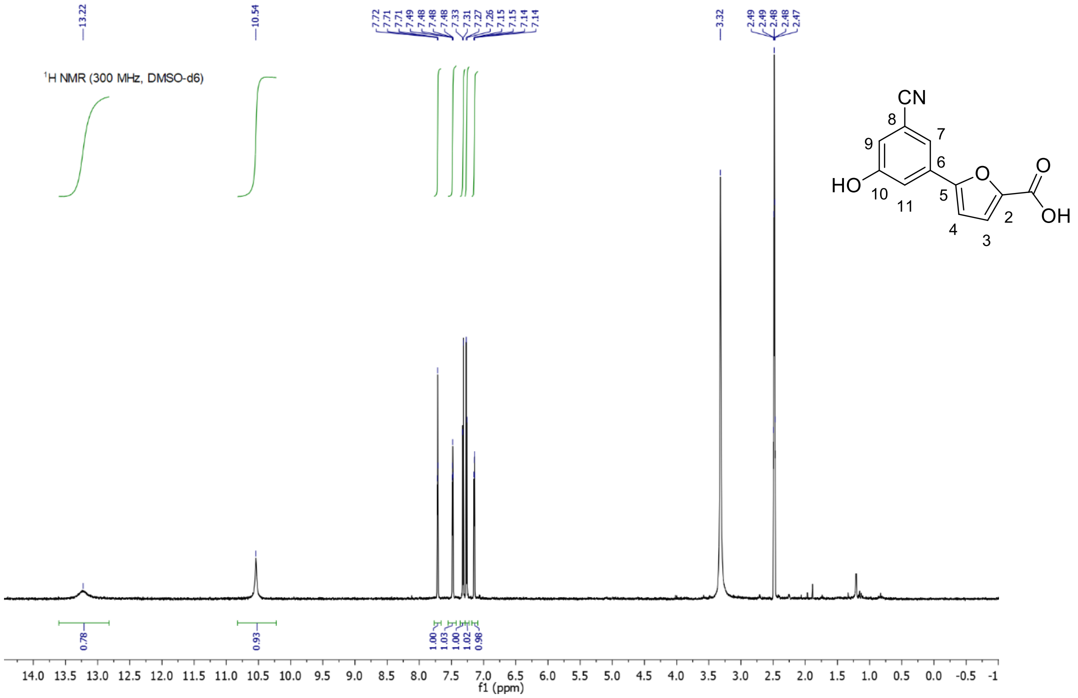

Figure S97. ^{1}H NMR spectrum of **IX**.

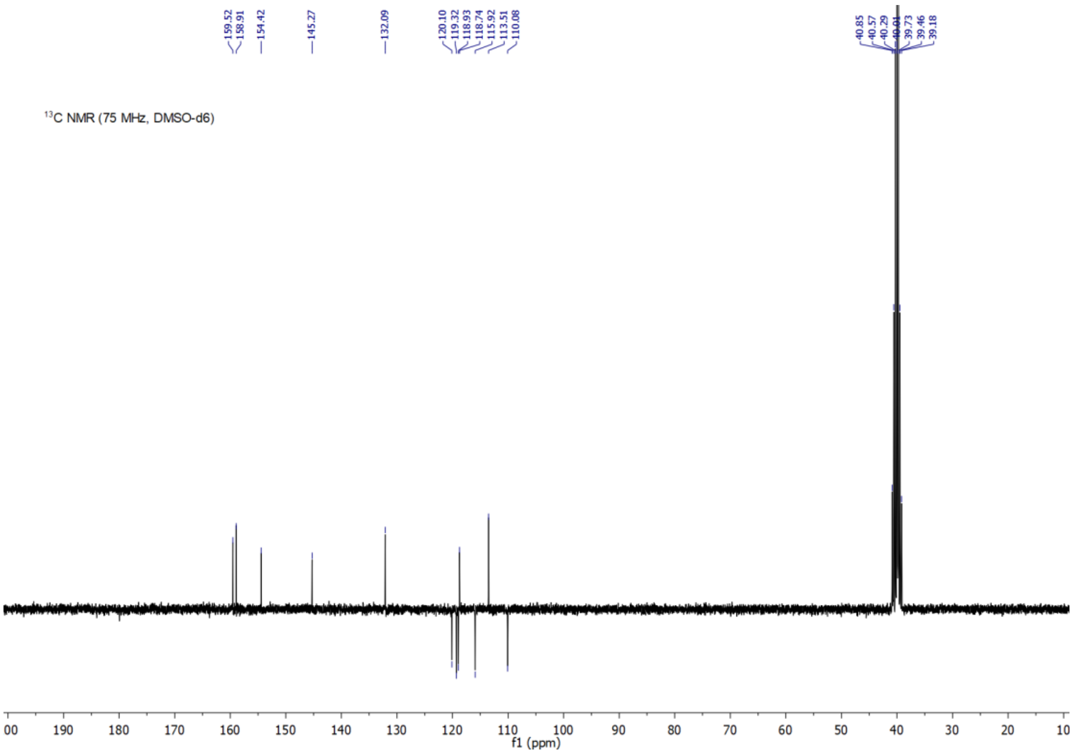

Figure S98. ^{13}C APT NMR spectrum of **IX**.

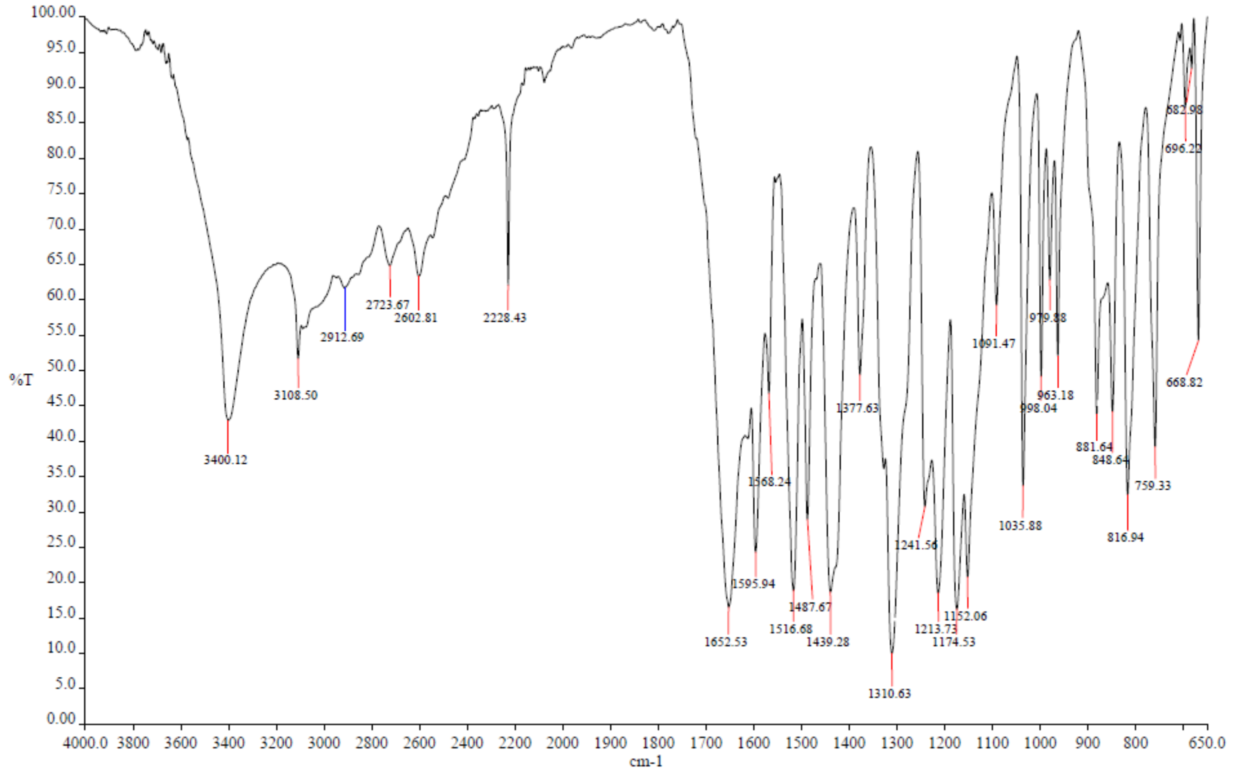

Figure S99. FT-IR spectrum of **IX**.

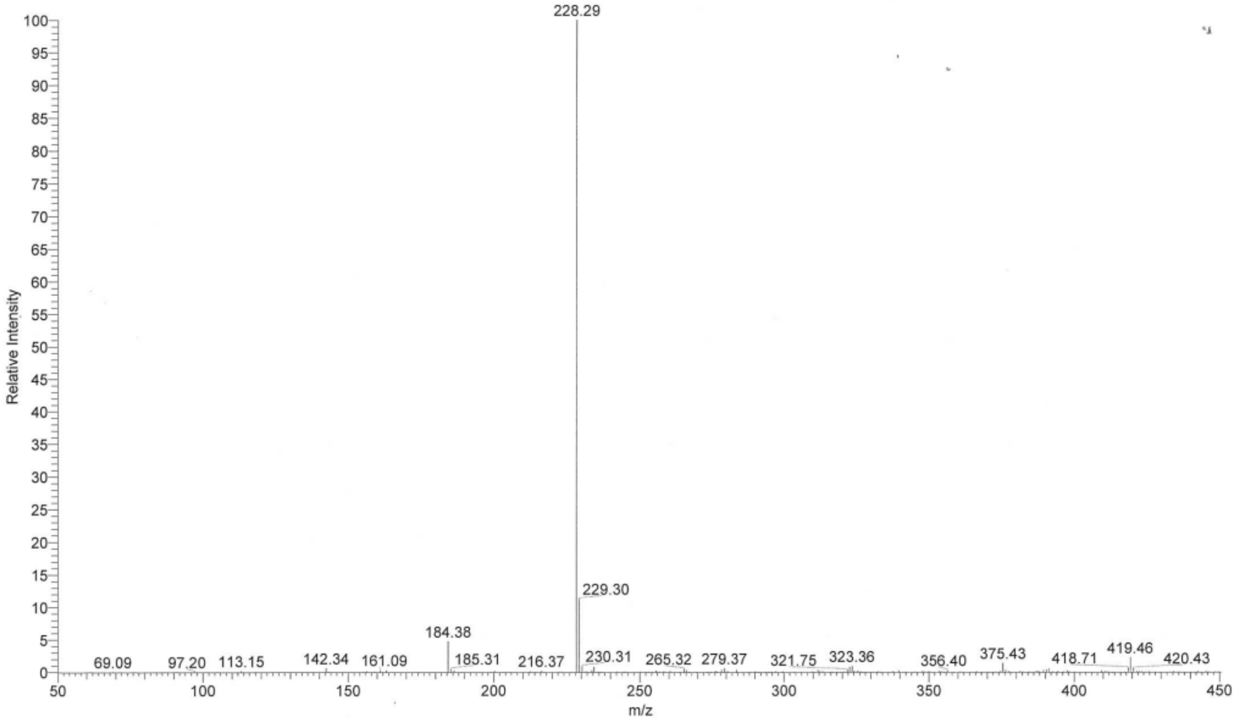

Figure S100. ESI-MS spectrum of **IX**.

47. **Methyl 5-(3-cyano-5-hydroxyphenyl)furan-2-carboxylate (IX-E)**

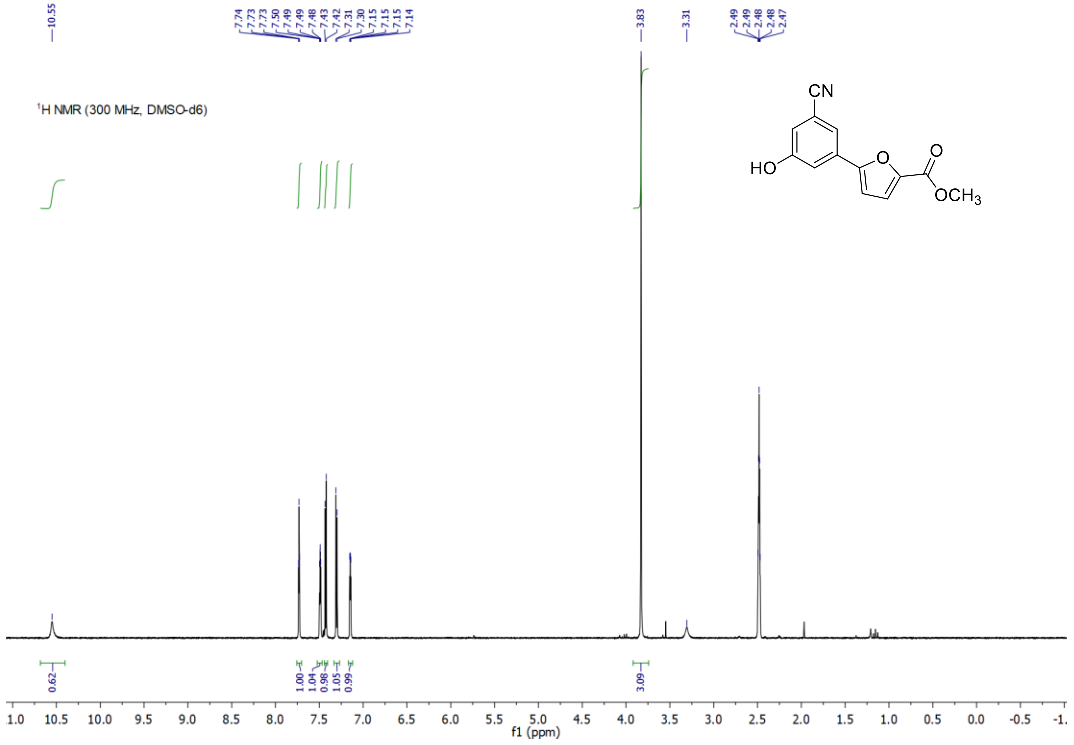

Figure S101. ^{1}H NMR spectrum of **IX-E**.
